# Bridging the health literacy gap: Women and community stakeholders’ perspectives on maternal and child health communication in peri-urban Karachi, Pakistan

**DOI:** 10.1371/journal.pdig.0001632

**Published:** 2026-08-27

**Authors:** Sameeta Kumari, Ayesha Khalid, Hajra Malik, Mahim Maher, Muhammad Imran Nisar, Fyezah Jehan, Zahra Hoodbhoy

**Affiliations:** Department of Paediatrics and Child Health, Aga Khan University Hospital, Karachi, Pakistan; Harvard University, UNITED STATES OF AMERICA

## Abstract

Health literacy significantly influences health outcomes, particularly in Low-and Middle-Income Countries. Communication of maternal and child heath messages occurs via text messages, phone calls, television, newspaper articles, and home visits. An exploratory qualitative study was conducted in four peri-urban sites of Bin Qasim Town, Karachi to explore the perspectives of women and community stakeholders on effective health messaging strategies in peri-urban Karachi, Pakistan. Seven focus group discussions (FGDs) with women of reproductive age, and seven key informant interviews (KIIs) with the community stakeholders were conducted. Women were randomly selected from the surveillance database, while stakeholders were purposively sampled. Audio-recorded FGDs and KIIs were manually transcribed and analysed using thematic analysis. Three major themes emerged: (i) the growing but constrained role of digital platforms, (ii) facilitators and barriers of health message dissemination, and (iii) community-suggested solutions. While digital tools like WhatsApp and TV were popular among smartphone users, barriers included phone access low digital literacy and unreliable electricity. Door-to-door visits by trusted community health workers remained crucial especially for those without little access. Participants recommended hybrid strategies including public announcements, community theater and visual media. To conclude, a hybrid communication approach that integrates digital tools with traditional outreach is critical in peri-urban Karachi. Interventions must prioritize equitable digital access and culturally relevant strategies to bridge health literacy gaps and improve maternal and child health outcomes.

## Author summary

In this study, we wanted to understand how women and community leaders in peri-urban areas of Karachi receive and use important health information, especially about pregnancy and childbirth. We found that although digital tools like mobile phones and social media are becoming more common, many families still face challenges in using them regularly. These include limited access to smartphones, unreliable electricity, lack of time and in some cases, limited decision-making power among women.

One of the key findings was the continued importance of face-to-face interactions in sharing health information in these areas, with community health workers who visited homes, being the most trusted source of information. At the same time, we saw that simple, engaging and visual methods like videos, posters, mosque announcements, and even small theatre performances can help make health information easier to understand and more accessible in these communities.

Overall, our findings show that digital tools can support health communication, but they cannot replace human connection in these settings. We believe that combining technology with community-based approaches is the most practical and meaningful way forward to improve health awareness and outcomes for mothers and children in underserved communities.

## Introduction

Health literacy is an important social determinant of health that serves as the foundation for effective health promotion [1]. The World Health Organization (WHO) defines it as an individual’s ability to obtain, comprehend and apply health information to maintain and improve their own health while also contributing to the well-being of their families and communities [2]. Globally, health literacy has received substantial attention due to its direct impact on health outcomes [3]. According to a recent report from the US Department of Education and the National Centre for Education Statistics, one in four parents have poor health literacy, resulting in issues such as poor child nutrition, medication errors, and higher emergency department visits [4]. This challenge is more pronounced in Low- and Middle-Income Countries (LMICs), where inadequate education, high healthcare costs and various socio-cultural barriers further hinder access to reliable health information [5,6].

Health related information can be disseminated through various mediums including text messages, phone calls, television, newspaper articles, and community group gatherings [7]. The effectiveness and acceptability of these communication channels often depend on factors such as access, trust in the information source, and the extent to which the communication modality aligns with community needs and technological realities [8]. In limited resource settings particularly, community-based approaches, particularly home-to-home visits by community health workers (CHWs), have proven to be highly effective in improving health literacy [9]. A recent study among the rural residents of South Asia and Africa explored the effectiveness of participatory learning and action cycles, in which local facilitators made home visits to educate pregnant women about safe birth practices and newborn care; this resulted in a significant increase in knowledge, confidence, and the acceptability of recommended practices [10].

While these traditional outreach methods remain important, digital health messaging has emerged as a powerful complementary tool for health promotion [11]. A systematic review highlighted that social media plays a significant role in health communication by providing peer, social, and emotional support to the general public, while also facilitating the exchange of health messages [12]. Knop et al. further reported that Mobile Health (mHealth) interventions can improve antenatal care attendance and the timeliness of child immunizations in LMICs, reinforcing the potential of digital tools in maternal and child health [13]. However, a global scoping review of digital health interventions found that most evidence on digital health promotion is disproportionately focused on high-income countries [11]. This imbalance limits the applicability of the findings to LMICs, where differences in healthcare infrastructure and access to digital tools are also more pronounced [14]. As of January 2025, Pakistan Telecommunication Authority reports that mobile cellular subscriptions cover approximately 79.30% of the population, while mobile broadband penetration stands at 57.08%, highlighting a gap in high-speed internet access [15]. Kazi et al. also assessed mobile phone access in both urban and rural regions of Pakistan and found that while mobile phone ownership is high, smartphone penetration remains limited. This indicates that a significant portion of the population still relies on basic phones with limited functionality, posing challenges for digital health interventions [16].

Unlike urban areas, where robust digital and physical infrastructure is developed or rural areas with community health networks stabilized by long standing social ties, the peri-urban communities of Karachi remain caught in a transition gap with rapid, unplanned urbanization, high population density, and a transient, migrant workforce [17]. Therefore, digital penetration remains limited and door-to-door outreach is a vital method for health promotion. However, this method is often expensive, resource intensive, and requires significant time [9,18]. While previous research and United Nations has largely identified structural barriers such as limited phone access, unreliable electricity, and restrictive gender norms, few studies have examined how these factors interact with social trust, community dynamics, and local communication preferences to shape the acceptability and effectiveness of health messaging [19,20]. Given these challenges, there is a need to identify communication channels that align with the community’s current technological realities. This study extends existing literature by conceptually framing peri-urban areas as unique transitional zones with hybrid communication ecosystems that combine human interaction with digital channels.

This aims to address the gap by exploring the perceptions of women and community stakeholders regarding appropriate health communication channels in these areas and identifying the most appropriate and culturally acceptable health communication channels. This will help in enhancing health message dissemination by guiding the development of targeted interventions to bridge healthcare knowledge gaps and improving maternal and neonatal outcomes in the future.

## Materials and methods

### Ethics statement

This study was approved by the Ethics Review Committee of Aga Khan University (AKU) (ERC # 2024-10183-29874).

### Study design and setting

This exploratory qualitative study was conducted from August to December 2024 in four peri-urban sites situated along the coastal belt of Bin Qasim Town, Karachi. These sites were purposively selected as they fall within the catchment area of a maternal and child health surveillance system run by the Department of Paediatrics and Child Health at the Aga Khan University since 2010. This provides an existing line listing system in the communities which could be leveraged to efficiently identify and recruit study participants. Collectively, these sites have a population of approximately 550,000 people, predominantly of Sindhi ethnicity [21].

For most residents, fishing serves as the primary source of income. Importantly, the four sites also share similar socio-demographic and health system characteristics, including population composition, service delivery structures, and exposure to Community Health Worker (CHW) - led maternal and child health interventions, and were therefore considered a relatively homogeneous setting for the purpose of exploring health communication practices.

As all areas are served by PHCs integrated within the surveillance setting, CHWs from the same communities conduct quarterly household visits to document vital events such as pregnancies, births and deaths. They also provide the local residents with preventive and promotive messages about antenatal care, skilled delivery, postnatal care, nutrition and childhood immunization. All data is collected electronically using an Android-based application, which is then securely stored in a cloud database.

### Study participants

This study included two participant groups: women and community stakeholders.

A system-generated list of married women of reproductive age was extracted from the surveillance database. Only those who had resided in one of these catchment areas for at least six months, had at least one child under five years, and were able to communicate in Urdu, were eligible for inclusion. From the final list, 20 women were randomly selected, and then the first 10 eligible participants were approached. If a woman declined, the next eligible individual on the list was contacted.

Community stakeholders, including political leaders, teachers, social activists, and religious figures, were identified using purposive sampling. They were intentionally kept heterogenous to involve different aspects of community health communication and service delivery and capture a broad range of perspectives on health messaging within the study setting. Only those with a minimum of one year of community service in the catchment area were eligible for inclusion. Their identities and credentials were verified by personnel at the PHCs.

### Data collection

Data for this study was collected through focus group discussions (FGDs) with women and Key Informant Interviews (KIIs) with community stakeholders. The FGD and KII guides were developed after an extensive review of relevant literature from LMICs (refer to S1 and S2 Texts) to explore key factors related to health communication in peri-urban slums. The guides captured participants’ demographics and examined sources and channels of health information, including traditional (TV, radio, community leaders) and digital platforms (mobile phones, social media). They explored the perceived effectiveness, frequency, and trustworthiness of these channels, along with barriers to access such as socioeconomic constraints, limited smartphone availability, unreliable electricity, and language challenges. Both guides also addressed cultural and social norms (e.g., gender dynamics, restricted mobility, and unequal access to technology) that influence how health messages are received and understood. Additionally, they investigated the role of community leaders and existing health campaigns, levels of community engagement, priority health concerns for women (such as hygiene and communicable diseases), and recommendations for improving the reach and effectiveness of health communication strategies.

The eligible women and community stakeholders were invited on a pre-decided date, and time at the PHC located at each site. They were identified and approached with the support of Community Health Workers (CHWs), who facilitated initial contact and mobilization within the community. Given their established relationships and trust within the community, CHWs played a key role in identifying eligible participants and coordinating attendance. To minimize potential influence on participation and responses, all FGDs and KIIs were conducted by trained research staff independent of CHWs, and participants were informed that their responses would remain confidential and would not affect their access to healthcare services. The data was collected from 21^st^ August 2024–23^rd^ December, 2024. Informed consent was obtained for both participation in the discussion and audio recording. In particular, participants without formal education were requested for thumb impressions as a means of consent, and an educated witness also signed their consent forms.

The duration of each interview and discussion was 30–45 minutes. Two researchers were always present at the time of interviews to ensure rigor. One researcher conducted the interview, while the other took detailed notes. SK and HM fully transcribed the audio recordings into Romanized Urdu to preserve the original meaning of the transcripts. The transcripts were subsequently translated into English by SK and HM for coding and inclusion in the manuscript. All translations were reviewed by SK, AK and HM to ensure accuracy and minimize potential misinterpretations.

The study team initially aimed to conduct 1–2 FGDs per site with 7–8 women per FGD and 1–2 KIIs per site, based on prior qualitative research norms and the need to capture diverse perspectives across communities [22]. Sample size was guided by the principle of thematic saturation, defined as the point at which no new codes or themes emerged from the data. Saturation was assessed separately for FGDs and KIIs through an iterative process of concurrent data collection and analysis. After each round of interviews, the study team reviewed audio recordings, field notes, and transcripts, and conducted preliminary coding to identify emerging themes. A saturation grid was used to track the appearance of new codes across interviews within each participant group. or until data saturation was achieved in these communities. By the seventh FGD and seventh KII, no substantially new themes or insights were identified, indicating saturation had been reached across both groups. This systematic and parallel assessment across FGDs and KIIs supported the adequacy of the final sample size while ensuring comprehensive coverage of perspectives relevant to the study objectives.

### Data analysis

The six-step method of thematic analysis described by Braun and Clarke guided the analytical process [23]. This method involved familiarizing with the data, generating codes, clustering codes into themes, reviewing and refining the themes, assessing their significance, and finally reporting the findings. Interview transcripts, notes and observations were thematically analysed using an inductive approach by SK, HM and AK. To facilitate concurrent data collection and analysis, transcripts were transcribed and translated into English by SK and HM after each interview and discussion respectively (refer to S3 and S4 Texts). The coding process was carried out using Dedoose software version 9.2.22 by SK, AK and HM and codes and themes were primarily derived from participants’ narratives and recurring patterns identified during analysis. After generating initial open codes from the raw data, codes were subsequently grouped into related categories based on conceptual similarities. These categories were then iteratively refined into broader themes through ongoing comparison across transcripts and discussions among the research team. The data was systematically structured and categorized into themes that aligned with the research objectives, including the growing but constrained role of digital platforms, various facilitators and barriers to effective health message dissemination and community-suggested solutions.

The researchers (SK, AK and HM) iteratively refined the codes and identified additional themes as the analysis progressed. This process involved multiple coders revising the codebook through team consensus, ensuring consistency in coding across all transcripts. To maintain qualitative rigor, transcripts were reviewed multiple times by SK, AK and HM and any discrepancies were resolved collaboratively. Coding disagreements were also discussed in regular analytical meetings until consensus was achieved, and revisions to codes and themes were documented within the evolving codebook and analytic notes to maintain transparency and consistency. After a comprehensive review, a final set of codes was developed, accompanied by relevant data extracts that were organized into broader categories using MS Excel. At the end, senior authors (ZH, IN and FJ) reviewed these themes to ensure accuracy and validity.

### Positionality statement

As a qualitative exploratory study, careful attention was given to issues of identity, positionality, and reflexivity. The research team comprised six individuals with diverse levels of experience in maternal and child health. Interviews were conducted by early-career researchers with medical backgrounds (SK and HM) under the guidance of senior researchers with public health expertise (AK, ZH, IH and FJ), ensuring both ethical integrity and methodological rigor. Throughout data collection and analysis, the researchers engaged in reflexive discussions to critically examine how their medical training, work experience in these communities, prior assumptions, and social positions could influence data interpretation and theme development. These reflections informed analytical decisions by encouraging the team to remain grounded in participants’ narratives and to challenge preconceived interpretations during coding and thematic refinement.

Researcher positioning—often conceptualized in terms of being an “insider” or “outsider” to the study population—was complex in this context. Although the researchers and participants were from the same city, the researchers’ relative material privilege, largely shaped by their class position, created a disconnect from the lived experiences of the marginalized and vulnerable communities under study. Their understanding of the topic was primarily shaped by professional exposure rather than personal experience.

The team’s affiliation with Aga Khan University and, by extension, the PHC, also played a significant role in shaping the research process. This institutional connection fostered trust and facilitated community access, owing to the university’s established presence in the area. However, it also highlighted differences in social, economic, and professional status between the researchers and participants. To mitigate the influence of a predominantly biomedical perspective, deliberate efforts were made to value participants’ voices. Interviews were conducted in a way that recognized participants as experts in their own lives, encouraging open and honest sharing of their experiences and perspectives.

## Results

We approached 220 women of which 55 agreed to participate in the study. We conducted 7 FGDs with women who consented and 7 KIIs with community stakeholders. Table 1 summarizes the demographic information of the 55 women and 7 stakeholders included in the study. The mean age of women was 27.05 years (SD = 5.43), with ages ranging from 19 to 40 years. About 43.6% (n = 24) of the women had no formal education and nearly half of the women (n = 25) owned mobile phones. Among mobile phone owners, 15 (60%) were smartphones users. Of these, 11 (73.3%) had attended some formal schooling while 4 (26.7%) had no formal education.

**Table 1:** Baseline characteristics of the participants enrolled in the study.

Women (n = 55)	n(%)
**Age in years (Mean±S.D**^**a**^)	27.05 ± 5.43
**No. of children under 5 years**	
1	41 (74.5%)
2	10 (18.2%)
3	4 (7.3%)
**Education**	
No formal Education	24 (43.6%)
Primary	10 (18.2%)
Secondary or higher	21 (38.2%)
**Owner of mobile phones**	25 (45.5%)
Basic mobile phone	10 (40%)
Smartphone	15 (60%)
**Community Stakeholders (n = 7)**	**n(%)**
**Age, years; median (range)**	27 (22-60)
**Gender**	
Male	3 (42.9%)
Female	4 (57.1%)
**Education**	
Secondary	1 (14.3%)
Higher Secondary	6 (85.7%)
**Occupation**	
Social Activists	4 (57.1%)
Vaccinator	2 (28.6%)
Teacher	1 (14.3%)
**Community work, years; median (range)**	11 (5-40)

a S.D. = Standard Deviation.

The community stakeholders had a median age of 27 years (range 22–60), and most of them had completed higher secondary education (n = 6, 85.7%). The various occupations among stakeholders included social activists (n = 4, 57.1%), vaccinators (n = 2, 28.6%), and teachers (n = 1,14.3%). They also had extensive experience of working within their community, with a median duration of 11 years (range 5–40 years).

Thematic analysis revealed three major themes from both the participant groups including (i) the growing but constrained role of digital platforms, (ii) facilitators and barriers of effective health message dissemination and (iii) community-suggested solutions. Although presented as distinct themes, considerable overlap existed between facilitators, barriers, and suggested solutions, reflecting the interconnected nature of communication practices within peri-urban communities.

### The growing but constrained role of Digital Platforms

Participants had varying perspectives on the use of digital platforms for accessing health-related information. While some women mentioned barriers to accessibility of mobile phones, smartphone users highlighted smartphones as one of the main digital platforms for acquiring health related information as well as for receiving calls from the PHCs. This suggests an emerging reliance on digital communication among connected households, while simultaneously highlighting unequal access within the community. One participant stated,

*I use it to consult a doctor regarding my child.“* (Woman #2 in FGD 5).

Smartphone users, in addition, reported frequent use of social media applications such as YouTube, Facebook and WhatsApp. A few community leaders also expressed the use of WhatsApp groups for sharing health tips and addressing vaccine hesitancy in the communities. These findings indicate that digital platforms may extend beyond information sharing by also serving as spaces for peer influence and trust-building within social networks. A key informant described,

“*We have our own WhatsApp group, and we post statuses there. My friends are part of the group, and some of them (group members) are even (vaccine) refusers. When they see these messages, they sometimes agree with us.” (KI6).*

Other social media platforms were also acknowledged by women and community stakeholders. In particular, a social activist stated that NGO workers and community members have been creating vlogs to showcase local skills and address health topics,

*“Now we have worked on introducing our educated youth to YouTube and Facebook, where they (NGO workers) create vlogs related to this community and their skills.” (KI1)*

Beyond mobile-based communication, participants also suggested integrating health-related information into news segments on private TV channels during peak viewing times. A respondent stated,

“*Many people watch the news. It would be ideal to have health-related information on the same channel. Husbands turn on the TV in the morning before going to work, so many people would see it. In the evening, news is also watched…especially around 7 or 8 p.m.…The 9 p.m. news is also watched by many.” (Woman #8 in FGD 2).*

### Facilitators of Effective Health Message Dissemination

The use of visual content, such as videos and posters, was frequently mentioned as an effective way to convey health messages in these communities. Women particularly emphasized the need to adapt this content to the linguistic diversity of the locals for better understanding thus reflecting importance of aligning communication modalities with literacy levels and language preferences. A participant explained,

“*Watching videos isn’t difficult; if it’s just written, where’s the enjoyment? Watching is still enjoyable. We can listen to the video while doing work, which makes it easier. Reading requires taking out time.” (Woman #5 in FGD 1).*

One stakeholder stated,

*“You put up posters, and whoever sees them will know, for example, that this vaccine is good and not harmful. Gradually, they (the community) become convinced.” (KI5)*

Furthermore, both women and stakeholders identified that the established trust between CHWs and the local community influences the reach of health messages. While stakeholders mentioned the strong relationships they have built within the community, local women acknowledged the reliability and trust in local health workers. One participant explained,

*“We listen to them (health workers—polio workers, Sindh workers, and CHWs). They come regardless of time and explain things to us, which benefits us.” (Woman #8 in FGD 2).*

### Barriers of effective health message dissemination

Limited access to mobile phones was mentioned as a significant barrier to health promotion by many women in the discussions highlighting how technological inequalities may unintentionally exclude already vulnerable groups from digital health interventions. One woman explained,

*“We don’t have phones, so videos on WhatsApp don’t make sense to us. How would we watch them?” (Woman #5 in FGD 7).*

On the other hand, smartphone users and the stakeholders highlighted power supply issues, as an important barrier to access information provided through phones or television. Additionally, women expressed concerns about their limited time for using mobile phones, as household responsibilities often took priority. Access to technology alone may not ensure engagement with digital health messaging, as structural and gender-related constraints continue to shape media use.

*“We don’t get much time to use them (TV and mobile phone) … We only watch TV or use mobile phones when we have time, and sometimes we don’t have time for days.” (Woman #5 in FGD 3)*

Cultural norms, gender dynamics and limited education also hinder healthcare decision-making. Stakeholders highlighted how men’s opinions often outweigh women’s in household decisions, restricting women’s autonomy despite being aware of the health benefits heard through various communication channels. A vaccinator described this challenge,

*“Men’s opinions hold more weight. For example, when we go for vaccinations, women often say, ‘Ask my husband first, then I’ll proceed.” (KI3)*

A women similarly reflected the dependence on male household members for mobility and healthcare access, emphasizing how care-seeking is often delayed and negotiated around the husband’s availability rather than guided solely by perceived medical need:

*“We wait until evening. We wait for our husbands to come home from work, and then we go to the doctor. He’s the one who takes us—we don’t go alone”(Women #1 in FGD 7).*

### Community-suggested solutions

Given the limited access to modern communication tools and the barriers that hindered their use, door-to-door campaigns have remained crucial for disseminating health messages. Since traditional lifestyles still prevail strongly in the community and not every household has a mobile phone, one participant emphasized the following:

*“A team should come to our homes and share information. Many households have no knowledge of these things.... So, if a team visits, they can explain things properly and get our attention by saying, ‘Look, if you focus on this and do it, things will improve for you.” (Woman #8 in FGD 3).*

Stakeholders also highlighted the unique benefits of face-to-face interactions in building rapport with the community, as a vaccination supervisor explained,

“*The understanding we can achieve through face-to-face interactions can’t be replicated through mobile phones. Face-to-face, we can understand their mindset, and they can understand ours.” (KI6)*

While face-to-face interactions were widely preferred, some participants acknowledged the potential of digital platforms for health message dissemination. This overlap between traditional and digital approaches further emphasizes that participants did not perceive these communication channels as mutually exclusive, but rather as complementary strategies adapted to different contexts and levels of access. A user suggested creating WhatsApp groups for sharing health messages, similar to how she had one for her children’s school, where school notices were shared.

Beyond direct communication, women also acknowledged the role of public announcements in community spaces for conveying health-related messages. As one woman explained,

*“If there’s a vaccination team visiting, the announcement is made in the mosque. Many people hear it and go there.” (Woman #1 in FGD 6).*

Lastly, women and stakeholders identified health camps and theatre performances as effective methods for directly delivering health messages and services to the community. A social mobilizer shared how a theatre performance was used to reach those who struggled to understand traditional messages, explaining,

*“For communities that didn’t understand anything, we did a theatre performance…. It was easier, and it’s a simple way to make people understand. They all watch it with interest, and they understand it as well.” (KI5)*

Overall, there was convergence between women and stakeholders regarding the importance of trusted, face-to-face communication channels, particularly through CHWs as well as recognition of the growing but uneven role of digital platforms. However, women primarily emphasized day-to-day barriers such as limited access to devices, time constraints, and household responsibilities, whereas stakeholders more often highlighted structural constraints and system-level challenges that prohibited digital use.

## Discussion

The study explored the different perspectives of women and community stakeholders on maternal and child health communication in peri-urban Karachi. Findings highlight that while smartphone users accessed health information through social media, many faced barriers like limited phone access, power supply shortages and time constraints in using digital media. Additionally, household responsibilities often took priority, further limiting women’s ability to engage with digital platforms. Stakeholders similarly acknowledged the promise of digital tools but emphasized the continued need for trusted face-to-face interactions led by CHWs. These findings suggest that a hybrid approach, integrating digital strategies with existing door-to-door outreach led by CHWs, may improve health dissemination in similar settings.

Our study reported an increasing use of digital platforms by smartphone users, particularly social media on mobile phones. Consistent with other LMIC studies including Pakistan [9,24], smartphone users in our study accessed information via WhatsApp, Facebook, and YouTube supporting the growing role of social media in health communication. Abrejo et al. further emphasized that digital interventions can help enhance decision-making, reduce dependency on CHWs, and lower travel costs [24]. However, trust in digital health information remains a concern; as shown in India where only 35% of users trusted social media content, reinforcing that digital outreach alone is insufficient [25]. Even though the latter may be a valid concern, this theme of lack of trust in digital content did not appear in our study. This may be due to the lack of education in the majority of the women which would hinder them from identifying mis- or disinfoirmation.

While digital media holds significant potential for improving maternal and child health communication, limited mobile phone ownership and access remain major barriers [26]. Nearly half of the women in the current study did not own a mobile phone, and even among those who did, factors such as electricity shortages, digital literacy, and gender-based restrictions hindered their ability to engage with online health content. These findings mirror research from India’s BIMARU states and rural Nepal, where mHealth interventions have shown promise in improving maternal and child health outcomes but are impeded by socio-economic, infrastructural, and cultural disparities such as poor internet connectivity, lack of electricity and geographic constraints [27,28]. Such infrastructural challenges are commonly seen in South Asian settings which limit use of digital health especially in women. To address these challenges, innovative solutions have been introducedin rural India and Bangladesh, where solar-powered internet solutions have been implemented to ensure reliable connectivity in areas with frequent power outages [28].

Additionally, community stakeholders emphasized their pivotal role in health communication, mentioning trust and familiarity as key factors in effective message dissemination. This is similar to a recent study which reported that stakeholder engagement in health communication enhances the trustworthiness and acceptance of health messages [29]. A study conducted in rural districts of Pakistan also found that local influencers, particularly religious leaders, can add credibility to health messages, while CHWs aware of the local cultural practices can effectively address vaccine hesitancy [30]. Perspectives from SAARC countries further report the important role of CHWs in improving maternal and neonatal healthcare outcomes, as their involvement in antenatal and postnatal care has significantly improved birth outcomes and newborn care practices in the underserved communities [31,32].

Previous research has emphasized the effectiveness of traditional communication methods in disseminating health information, particularly in communities with limited access to digital technologies. A recent qualitative assessment of the Sukh Initiative in Karachi, Pakistan highlighted the effectiveness of door-to-door services and free camps in promoting contraceptive use among married men and women [33]. The current study has similar findings where door-to-door campaigns remain a vital approach for disseminating health messages. Furthermore, public announcements in community spaces play a pivotal role in public health promotion, as expressed by the study participants. A scoping review also identified various health-promoting interventions conducted within mosques, emphasizing their effectiveness in fostering positive health behaviours and addressing communication barriers within Muslim communities [34]. This highlights the continued need for in-person communication as trusted stakeholders and front line care providers remain pivotal sources of health information in marginalized communities.

Various organizations such as WHO and CDC suggests the use of interactive strategies to enhance understanding and engagement for health communication and behaviour change interventions [35,36]. Participatory communication approaches, which involve engaging communities through interpersonal interactions, using visual content and multi-channel media, have been shown to cultivate community interest and active participation in health-related matters [37]. A rapid review on participatory health interventions across various public health domains emphasized the benefits of integrating digital formats, demonstrating their role in fostering greater community engagement [38]. Additionally, participatory communication strategies like community theatre, particularly in young adults, have proven to be effective in promoting well-being and raising awareness of critical healthcare issues [39]. Our findings resonate with these studies, reinforcing the continued relevance of traditional communication methods and emphasizing the need for hybrid strategies that integrate both conventional and digital approaches to enhance health message dissemination and reach.

A major strength of our study is the inclusion of both message recipients and disseminators, offering a comprehensive overview of communication dynamics. Regular and detailed debriefings during data collection also enhanced reflexivity and analytical rigour. However, several limitations should be considered. Of the 220 women approached, only 55 participated, introducing the possibility of selection bias. Women who agreed to participate may have been more engaged with health services, more available, or more comfortable discussing health-related topics, while those who declined may represent more marginalized or harder-to-reach groups with differing perspectives. This non-participation may have led to an overrepresentation of relatively health-aware individuals, potentially shaping the findings toward more favorable perceptions of existing communication channels. Furthermore, as our focus was in maternal and child health messages, we included only women with at least one child under five; this targeted inclusion may not fully capture the preferences of older women and men, who often play an important role in household decision-making resulting in a constrained understanding of intra-household communication dynamics. Additionally, our study was conducted in a specific region of Pakistan, potentially limiting the generalizability of findings to other areas or countries with different socio-cultural contexts. Participant responses may have been influenced by social desirability bias, particularly in discussions related to health behaviors and practices, and may also have been shaped by the involvement of CHWs in participant recruitment, although they were not involved in the actual FGD or KII. Responses may further have been shaped by the researchers’ institutional affiliation, which could have affected participants’ willingness to respond openly.

## Conclusion

In conclusion, while digital platforms present opportunities for expanding maternal and child health communication, they must complement, not replace face-to-face methods. A hybrid communications strategy grounded in local realities and community trust is crucial in bridging health literacy gaps and improving maternal and child health outcomes in underserved areas. Moving forward, future research should aim to evaluate the effectiveness of specific hybrid communication models across diverse settings, particularly in LMICs, and explore how digital tools can be adapted to varying levels of literacy and technology access.

This study emphasizes the importance of investing in integrated communication approaches that combine digital tools with community-based outreach, and training frontline health workers in digital facilitation. Policy makers need to prioritize these targeted interventions to enhance engagement, build trust, and ultimately contribute to more inclusive and effective maternal and child health systems in underresourced settings.

## Supporting information

S1 Text This contains the guide used for FGDs conducted with women. (DOCX)

S2 Text This contains the guide used for KIIs conducted with community stakeholders. (DOCX)

S3 Text This contains the translations of all FGDs conducted as part of this study. (DOCX)

S4 Text This contains the translations of all KIIs conducted as part of this study. (DOCX)
