# Supplementary material for: Bridging the health literacy gap: Women and community stakeholders’ perspectives on maternal and child health communication in peri-urban Karachi, Pakistan
Source: PLOS Digit Health. 2026 Aug 27;5(8):e0001632. doi: 10.1371/journal.pdig.0001632 (PMC13521370; doi:10.1371/journal.pdig.0001632)
Supplement: Supplementary file 1 [file pdig.0001632.s001.docx]

**Title: Bridging the Health Literacy Gap: Women and Community Stakeholders’ Perspectives on Maternal and Child Health Communication in Peri-urban Karachi, Pakistan**

**Guide for Focus Group Discussions with Women**

Salam everyone, I am Sameeta Kumari, working as a Research Associate at the Aga Khan University Hospital. Welcome to our group discussion. Today, my team is here to talk about how health information spreads in your community. Specifically, we are interested in exploring how you get health information in this community. Through our discussion surrounding different methods for getting health-related information, we hope to have an idea that will help us in sharing health-related messages in the future. We hope that everyone will feel comfortable sharing their experiences freely. We may at times interrupt to keep sure we have enough time to cover all of our questions. If any of our questions are unclear, please let us know and we can explain. We will record our discussions. You can stop me at any point if you have any questions or if there's something you'd like to clarify.

Data Collection Date_______________________

Start time/End time___________________________

FGD Location _______________________________

Number of Participants________________________

Facilitator___________________________________

Notetaker___________________________________

| **Participant Number** | **Age**  **(Years)** | **No. of children under the age of 5 years** | **Personal Mobile Phone (Yes/No)** |
| --- | --- | --- | --- |
| **1** |  |  |  |
| **2** |  |  |  |
| **3** |  |  |  |
| **4** |  |  |  |
| **5** |  |  |  |
| **6** |  |  |  |
| **7** |  |  |  |
| **8** |  |  |  |

Icebreaker Question:

Before we dive into our discussion, I’d love to hear about how you usually spend your time here in the community. What are your hobbies? What activities do you enjoy in your free time, whether it’s spending time with your family, engaging in embroidery handwork, watching television, listening to radio or anything else that brings you joy?

Warm-up Question:

How important do you think is health in your community? Do you think keeping yourself and your surroundings clean helps reduce the spread of common diseases?

Main Questions:

1) Can you outline some of the common sources you rely on to receive general information in your community? What about health-related information specifically in your community?

Probe: Which sources (TV, Newspaper, Radio, cale, WhatsApp, FB, TikTok, others), Frequency of using these sources for health-related information, why do you trust these sources?

2) Can you describe what has been the best method to spread health-related messages or information in your community?

Probe: Health Campaigns, Nearby Health Clinics, Community Health Workers, Social media platforms

3) Can you explain the difficulties and problems you faced when accessing health-related information or messages? If yes, can you provide examples of specific difficulties?

Probe: Lack of smartphones, Unreliable Electricity, Socioeconomic Constraints, Language Barrier

4) Can you tell us the different digital platforms women use for accessing health information in your area? Probe: Mobile phones, TV, Radio

What are their benefits? Are there any challenges associated with these digital mediums?

Probe: How frequently do they use? If you can’t use them, why is that the case?

5) Can you explain any cultural and social norms in your community that stop you from receiving and understanding health-related messages?

Probe: Male dominance, One mobile phone per House, Discrepancies in Mobile phone ownership and lack of woman mobility.

6) Do you think community leaders have any role in spreading health-related messages? Can you share examples?

Probe: Any successful community lead initiatives

7) Are there any specific health topics or issues you feel are particularly important for women in peri-urban slums? If so, what are they and why?

Probe: Cleanliness, Communicable diseases

8) Is there anything else you would like to share about health information that we have not covered so far?
