# Supplementary material for: Bridging the health literacy gap: Women and community stakeholders’ perspectives on maternal and child health communication in peri-urban Karachi, Pakistan
Source: PLOS Digit Health. 2026 Aug 27;5(8):e0001632. doi: 10.1371/journal.pdig.0001632 (PMC13521370; doi:10.1371/journal.pdig.0001632)
Supplement: Supplementary file 2 [file pdig.0001632.s002.docx]

**Title: Bridging the Health Literacy Gap: Women and Community Stakeholders’ Perspectives on Maternal and Child Health Communication in Peri-urban Karachi, Pakistan**

**Guide for Key Informant Interviews with Community Stakeholders**

Demographics:

1. What is your age?
2. Gender
3. What is the highest degree or level of education you have completed?
4. What is your current employment status?
5. What is your current role in the community?
6. How long have you been involved in community service?

Main Questions:

1. What do you think about the health of people in the community?
2. Can you explain the role of community leaders in spreading health-related messages within peri-urban slums of Karachi?
3. What are some of the main problems you face when trying to communicate health messages effectively in your community?

Probe: Socio-economic constraints, Transport issues, Lack of smartphones

4. Which methods have you found to be the most useful in spreading health-related messages within peri-urban slums?

Probe: Health Campaigns, Social media platforms

5. Are there any specific channels or platforms that you prefer or rely on for sharing health information with the community members?

Probe: Radio, TV

6. How much do the community people take part in health-related activities and programs within your community?
7. Are there any specific cultural or social norms within your community? How do they influence the way health messages are received and understood?

Probe: Male dominance, one mobile phone per house, discrepancies in mobile phone ownership

8. What improvements or additional resources do you think are needed to increase the reach of health-related messages in your community?
9. Is there anything else you would like to share about health information that we have not covered so far?
