# Supplementary material for: Bridging the health literacy gap: Women and community stakeholders’ perspectives on maternal and child health communication in peri-urban Karachi, Pakistan
Source: PLOS Digit Health. 2026 Aug 27;5(8):e0001632. doi: 10.1371/journal.pdig.0001632 (PMC13521370; doi:10.1371/journal.pdig.0001632)
Supplement: Supplementary file 3 [file pdig.0001632.s003.docx]

**FGD 1**

**Interviewer:** Before we begin our main discussion, we’d like to ask how you all generally spend your time at home and in the community. What are your usual activities? Tasks around the house, cooking, etc.

Like sewing, watching TV, hand embroidery work.

**R1:** Watching TV, household chores, sewing.

**R2**: I enjoy watching TV.

**Interviewer:** Does anyone do something different?

**R3:** We look at our phones, do sewing, pray.

**R4**: Reading the Quran.

**Interviewer:** Alright, now can you tell us how important you think health is?

**Everyone:** Health is a huge necessity; it’s very important for children and mothers.

**Interviewer:** What else is essential here related to health?

**R3:** The treatment of children is the most important, but since our children are older, their treatment can’t be done here, which causes us a lot of worry and difficulty.

**R5:** They only treat pregnancy issues in women here, but don’t address other health problems. We wish they would treat those issues too, for which we have to travel far. We have to go very frequently, and the travel costs to the hospital are very high.

**R3:** My son also has a condition, and due to financial issues, we can’t go anywhere for his treatment. He’s now over 5 years old.

**R1:** I have four daughters. I haven’t been able to conceive again since one of my babies passed away a year ago. I took so many medications, some of them very expensive, but still haven’t been able to get pregnant. The treatment costs are high, and I have to go to the city, which adds more expenses. I would like to request that a solution for this kind of treatment be made available here.

**Interviewer:** So what are your thoughts on health? Who do you ask for advice?

**Everyone:** We generally ask our elders. Whatever they tell us, we listen to. That’s the only way; we trust the information we get from our elders.

**Interviewer:** Besides that, do you watch anything related to health on TV?

**R3:** We don’t really use TV for that. We rely on our elders because they have experience, so we follow their advice.

**Interviewer:** Does anyone else have a different approach?

**R3:** Initially, we go with the treatments suggested by our elders. If that doesn’t work, then, as a last resort, we go to a doctor.

**Interviewer:** Since most of you have mobile phones, do you use YouTube or other apps?

**R3:** We mostly watch YouTube; everyone watches YouTube.

**R2:** I also use Instagram, and some use Facebook as well.

**Interviewer:** When you use these apps, do you find any health-related information?

**R6:** Yes, if you like or subscribe to certain content, it appears. Both YouTube and TikTok show health-related videos, which we sometimes share. For example, information about alternatives to medicines, like cashews, nuts, and almonds. I mainly watch things that boost strength and health or are beneficial for my own issues.

**R5:** I mostly look up remedies for hair problems. Do you search for these on TikTok or YouTube?

**Interviewer:** In your opinion, what would be the best way to spread health-related messages to reach you all effectively? Could it be through TV, mobile, or something else? Since social media is widely used, how can health messages best reach you?

**R6:** Most of us use mobile phones. Nowadays, everyone has one.

**Interviewer:** Is there a specific app you use? Like Instagram, Facebook, WhatsApp, or TikTok?

**R5:** Yes, there’s a WhatsApp group for my daughter’s school, where we get school-related notices. You could create a similar WhatsApp group and share messages through it.

**Interviewer:** Can you explain the challenges and issues faced in accessing health-related messages or information? So, for example, how many households have mobile phones and internet availability? Who here has a mobile phone?

**R7:** Those who have a touchscreen mobile usually have internet access. If someone doesn’t have home internet, they get packages for it.

**Interviewer:** Is this a barrier for you at home? Or, do you experience any issues with low signal that affects communication?

**R7:** Sometimes it does happen; there are times when there’s no internet signal, and we can’t communicate.

**Interviewer:** If I were to send you a message, which networks or signals work best in this area?

**R7:** Jazz and Zong signals work better here, depending on the location of the towers.

**Interviewer:** How often do you experience power load-shedding at home?

**R8:** It’s quite frequent, and it’s a big problem. If you want to send a message on time without delays, there’s often an interruption.

**Interviewer:** How much of an interruption does it cause?

**R8:** Yes, it’s a real issue. Power is out for three hours at a time. It goes off at 10 a.m., comes back at 12:30 p.m., and so on.

**Interviewer:** Does this interrupt your use of mobile phones?

**R8:** Yes, we’re unable to charge our mobile phones during those times.

**R1:** It sounds like TV has become almost obsolete these days.

**Interviewer:** Do any of you have TVs at home?

**R8:** We don’t have a TV at home. We don’t have a touchscreen mobile either, just a basic one.

**Interviewer:** If we wanted to send you a message, how could we do it? Could we call your husband?

**R8:** He also only has a basic mobile phone. At home, we only have a basic mobile, no WhatsApp. The children don’t attend school either.

**Interviewer:** So you could communicate by phone calls on a basic mobile?

**R8:** Yes, we can talk on the basic mobile.

**Interviewer:** If we were to organize a session here, where those without mobile access could come, we could share information, like preventing diseases or infections or maintaining hygiene. Would you be able to attend such a session?

**R5:** Yes, we could come here if there was a session for those who don’t have access to these resources.

**Interviewer:** We want to find the best way to deliver our message to them. So, I’d like to ask your opinion.

**R5:** When field workers visit our homes, we benefit a lot from them.

**Interviewer:** Can you tell me more about them?

**R5:** They’re very good. Whenever they come, they call us and are very helpful, doing any work we ask of them.

**Interviewer:** I see, what makes them so helpful?

**R5:** If we call them in times of need, a vehicle comes too. Previously, they used to send a car for pickup and drop-off, but now they only provide drop-off. It costs around 300-400 rupees to get here.

**Interviewer:** Can you share if women in your area use different digital platforms to gather information? You mentioned TV and Instagram—what benefits do you see in using these?

**R2**: For the children, if I need help understanding something new, I search on the internet or check Instagram. I then understand how to make it, and it becomes easier. Previously, if we needed to send a message, we’d have to travel. That was in the old days. Now, we just call using a package, and we can communicate over long distances. If there’s a message, they just tell us over the phone.

**Interviewer:** So, is the phone the most convenient way for you? Is it better to speak on the phone than to visit in person? How many times a day do you use your phone?

**R3:** Two to three times a day, for YouTube, Instagram, and so on.

**Interviewer:** What do you usually watch?

**R3:** Mostly dramas.

**Interviewer:** Has anyone told you about blogs on YouTube in your community?

**R4:** Yes, the kids watch those. They’re the ones making vlogs and telling us about things. There’s someone named Sha Miran who talks about how they live by the sea and how they spend their time.

**Interviewer:** If there were a similar channel, would you watch it?

**R4:** Yes, we would watch the channel.

**Interviewer:** Are there any rules in your community that everyone must follow?

**R4:** Yes, it’s the husband who leads. He’s the head of the family, so we follow his direction.

**Interviewer:** Are all of your husbands fishermen?

**R4:** No, no, they go a little farther from Rehri Goth to work daily on wages. There used to be companies, but they closed down.

**Interviewer:** Who among you works?

**R5:** I go to work at a hotel for daily labor.

**The rest:** We stay at home.

**Interviewer:** Besides that, what role do community leaders play in health-related messages? For example, if there’s an announcement in a big mosque or an NGO leader in your area?

**R5:** Like Abdullah Rohani or Shafi Mahmood Hussain.

**Interviewer:** Who are these people?

**R5:** They’re the elders of this area. There’s a committee here, and they are the leaders. If anything goes wrong, people tell them.

**Interviewer:** Do they make decisions?

**R5:** Yes, they make decisions. If there are any issues with girls, people inform them, and they listen and decide.

**R3:** In our area, Daal Abad, we listen to our "Bhai Sahib" a lot. He’s the elder of our village.

**Interviewer:** So, he acts like a community leader?

**R3:** Yes.

**Interviewer:** I wanted to ask, you mentioned community leaders—what is their role in your area? Has any organization helped you, or helped anyone else?

**R4:** Yes, they help with household issues. For example, if there’s a problem in the house, they help with it.

**Interviewer:** Is there any other example? Can someone else give an example?

**R2 and R6:** That doesn’t happen in our area or neighborhood.

**Interviewer:** Okay, so does each family handle its issues individually?

**R2 and R6:** Yes. In our area, there is a committee, but it’s a Pathan committee. They don’t solve issues, and they don’t get involved in these matters. If there’s a conflict or household issue, they stay out of it.

**Interviewer:** So if there’s a committee, would it be able to deliver a health-related message to you?

**R2:** No, they just have a committee for the sake of it, but there’s no benefit from it.

**Interviewer:** Got it. How many people use mobile text messages?

**R3:** Only for talking to those who don’t have WhatsApp.

**Interviewer:** Is there a specific time when you all use mobile phones more frequently?

**R2:** At night, because the children are asleep, so one can use the phone comfortably.

**Interviewer:** And what kind of message format do you prefer? Do you like videos or written messages?

**R2:** I prefer written messages.

**Interviewer:** Do you prefer reading in Sindhi, Urdu, or English?

**R3:** English, I don’t like Urdu.

**R4:** No, if it’s in Urdu, it’s easier to understand. Many people prefer watching videos in Urdu. No one really reads English.

**Interviewer:** Do you also speak Sindhi?

**R4:** Yes, yes. But we understand Urdu better, and we can read Urdu.

**Interviewer:** Do you find it difficult to open videos to watch? If anyone finds it difficult to watch a video, can they read written content?

**R5:** Watching videos isn’t difficult; if it’s just written, where’s the enjoyment? Watching is still enjoyable. We can listen to the video while doing work, which makes it easier. Reading requires taking out time.

**Interviewer:** What do you think about sharing health-related messages on a poster here?

**R6:** That’s also a good way, I would like it.

**Interviewer:** Alright, what about sharing information on other topics here? For example, pregnancy is one topic. Are there other issues as well? Any major issues you think are important and should be shared on posters, messages, or videos?

**R2:** There are many issues, like not being able to have children or miscarriages.

**Interviewer:** We want to ask about cleanliness and children’s diseases.

**R1:** People here have little awareness about cleanliness. There’s a major issue of cleanliness here. There are some people who are very clean and understand its importance.

**Interviewer:** If we target this, maybe there could be some awareness in people. And how can we improve this to help people understand the importance of health?

**R2:** The cleaner the environment children grow up in, the healthier they’ll be. They’ll avoid diseases, and other illnesses in the household will be prevented.

**R4:** In this area, there are many people who understand, and there are also many who don’t value health at all.

**Interviewer:** So, ladies, regarding cleanliness, what would you suggest we share with others?

**R4:** Cleanliness, not eating dirt, washing hands, keeping the streets clean, children washing their hands before eating, maintaining cleanliness. Besides this, children should also be kept clean. The streets, children, and houses should all be clean, and the food should be good, so the children’s health will improve.

**Interviewer:** Would you like to share anything else?

**R5:** Like, I want to say that my son is eight years old, and he has some kind of allergy issue. He was fine until he was three. After three years, he developed an allergy, which started in his eyes and has now gone inside. He has a cough, and he’s affected by dust and rain. Rain is natural, but dust—I can’t protect him from that. I try hard to keep the house free from dust. I take care of his cleanliness a lot and tell him to be careful with where he puts his things. But even with all this, his chest hasn’t cleared for the past month and a half. I would really like it if there was some medication or solution to deal with his allergy. I’ve noticed two to three other kids in our neighborhood have started having similar issues.

**Interviewer:** Do you think it would be helpful for us to discuss this with you? To explain what might be causing the allergy, why his chest isn’t clearing up, and what steps you could take?

**R5:** Yes, I’ve tried everything, but as long as the medication is being used, it’s fine. When I stop it, I tell the doctor, and they say it’s just how it is, that there’s no cure. I want a solution, especially as more children in our area are experiencing this. I’ve seen at least two or three other kids with this issue. There might be some problem in the air—maybe moisture or something from the buses, or just a general lack of cleanliness in the area.

**Interviewer:** Have you ever sought treatment from the elderly or traditional midwives (dāis) in your community?

**R4:** We’ve never gotten treatment from midwives; we only go to doctors. All the children were born at home. We mainly listen to my mother-in-law; she runs the household according to her decisions. Midwives don’t have much of a role here.

**R3:** We only go to midwives for occasional check-ups; otherwise, we trust doctors more than midwives.

**Interviewer:** If you wanted to talk to someone wise in the community who understands local issues, who would you turn to? For example, if the mosque's imam or someone with community knowledge advised you, who would that be?

**R2:** We mostly just listen to my in-laws and my husband.

**Interviewer:** If we speak to them, would they discuss this with you?

**R2:** My husband definitely would. The suggestion you gave is also good. If you have any educational videos that attract children and are beneficial for their health, you could watch those videos.

**R5:** I feel that both TV and mobile phones can be both beneficial and harmful for children. Excessive use of TV and mobile phones isn’t good for them. But if you want to watch educational videos, you could watch those too.

**R6:** We prefer voice messages on WhatsApp, you can use those as well. Voice messages are often easier and more convenient to listen to, and one can listen to them whenever you want.

**Interviewer:** If your children go to school or madrasa, could you also talk about this there?

**R6:** Yes. If there are any awareness programs from the madrasa, watching those could also be beneficial. If you think any local organization or group could help, you could also speak to them.

**Interviewer:** Are there people here who cook food and sell it? Do you know any of them, or are you connected to them?

**R7:** No.

**Interviewer:** Thank you all very much.

**FGD 2**

Assalam-o-Alaikum, I am Sameeta, and this is Hajrah. We are both from AKU. As mentioned earlier, I will repeat this for the recording. Today, with your permission, we will talk about health-related topics. Specifically, how health messages can be delivered to your community and the people around you. What methods are used—do you use mobile phones, watch TV, or mostly interact with each other? We will discuss all of this. We will also record this conversation so that we can analyze it later.

Shall we start? Do you have any questions before we begin?

No, just start.

**Interviewer:** What do you all like doing at home?

**R1:** I like cleaning.

**R2:** I like ironing, organizing children’s clothes, and resting. I also clean the house.

**Interviewer:** Besides this, does anyone enjoy watching TV or sewing?

**R3:** I like sewing.

**Interviewer:** In addition, how important do you think health is?

**R4:** It is very important. For example, I don’t let my kids eat junk food from outside because it makes them sick, and we have to go to the hospital.

**R5:** Health is important, but no matter how much you restrict children, they still eat juice, chips, and such.

**Interviewer:** Would anyone else like to share something about health?

That’s all.

**Interviewer:** In your opinion, how much does keeping yourself and your surroundings clean reduce the spread of common illnesses? Do you feel that cleanliness helps reduce diseases?

**R3:** Cleanliness is essential. It’s obvious that the more cleanliness we maintain, the fewer diseases will spread. I even cut my child’s nails—she’s my only one—and I pay a lot of attention to hygiene.

**Interviewer:** Besides this, does anyone else want to share how much you think maintaining cleanliness reduces illnesses?

**R2:** I pay attention to cleanliness at home. It’s evident that the more we focus on it, the fewer diseases will spread.

**R6:** The cleaner we keep things, the less dirt will spread. There will be fewer germs and mosquitoes because the environment here is such that mosquitoes are abundant. For example, when it rains, water collects on the roads, and cleanliness isn’t maintained. Even if you keep children at home, they eventually go outside to get something. There’s so much dust in the air that it causes breathing problems.

**Interviewer:** Now, let’s move on to our main question: can you name common sources in your community that people trust for general information, especially regarding health-related information?

**R8:** When my daughter wakes up in the morning, the first thing I do is wash her face, then give her breakfast. After that, I take her outside because kids can be persistent. Then I keep her at home or visit her cousin’s or aunt’s house, because sometimes you feel restless staying in one place.

**Interviewer:** My question is, out of options like TV, newspapers, radio, or mobile phones, which one do you use the most?

**R8:** My daughter watches poems on a mobile phone at my younger sister’s house, but I don’t use a mobile phone myself. I don’t have one. We have a TV at home.

**Interviewer:** Apart from this, can anyone else share what you use to receive messages, so we can know the best way to reach you?

**R7:** I don’t use a mobile phone. The kids watch TV and play at home. I have a basic phone with a Zong SIM card.

**R3:** I have a Warid SIM card.

**Everyone else** had Zong SIM cards.

**Interviewer:** If a message were sent to your basic phone, would you read it?

**R1:** I have both a basic and a smartphone.

**Interviewer:** Okay, now tell me, who among you uses WhatsApp?

**R1:** I use WhatsApp.

**R3:** My sister has it.

**R6:** I also use it.

**R7:** I also have WhatsApp. If a message is sent on WhatsApp, we do look at it.

**Interviewer:** Apart from this, platforms like TikTok, YouTube, and Facebook—out of these, which ones do you use the most?

**R4:** TikTok is used the most. Kids also use TikTok more; they mostly watch reels. I watch it less because it makes my eyes watery, and the doctor has advised against it.

**Interviewer:** Okay, can anyone else share how we should contact you?

**R5:** There’s only one phone at home, and my husband has it. It has a Telenor SIM card.

**Interviewer:** And what about you? What do you have at home?

**R2:** I have a basic phone, and others in the family have a smartphone. We also have a TV.

**Interviewer:** If a message needs to be delivered, what do people mostly use?

**R2:** I use my phone sparingly. My family uses touch (smartphones), so it’s better to send messages on touch phones. We also have a TV, but we don’t let the kids use it because it’s not good for them. It’s better to contact my husband and send him the message.

**Interviewer:** Can you all share the best way to spread health-related messages or information in your community? If we want to deliver a health-related message to you, what would be the most effective method?

**R1:** On WhatsApp, people check it first.

**R6:** Any media platform is fine, as long as it’s online.

**Interviewer:** Which online platform, WhatsApp or YouTube?

**R6:** YouTube or WhatsApp. For example, if a reel is sent showing how to care for children or maintain cleanliness around you, people will follow it.

**Interviewer:** So, if we send a video or link on WhatsApp, will people follow it?

**R6:** Those who are inclined to follow it will do so, while others might not.

**Interviewer:** If we ask, should we send videos or messages on WhatsApp, which one do people in your community prefer to follow?

**R6:** Video, because it’s easier to understand. Not everyone reads messages.

**Interviewer:** And about the language of the video, what should it be?

**R1:** It should be in multiple languages—Urdu, Sindhi, etc. Since most people here speak Urdu and Sindhi, Urdu speakers might not understand Sindhi, but Sindhi speakers can understand Urdu.

**R8:** Videos are better because they convey the message through conversation, like explaining how to take care of children and maintain cleanliness. But even if we maintain cleanliness, it’s not the same around us.

**Interviewer:** If we ask those who don’t have smartphones or only basic phones, how can we get the message across to them? Should we organize health campaigns or have women visit homes to share the message?

**R5:** All my brothers have smartphones and are always around. If there’s a video, they can show it to me. I’ll ask them to let me watch it.

**Interviewer:** If we ask whether health campaigns, setting up camps, or visiting homes individually would work, which of these methods would be suitable?

**R5:** Yes, visiting homes and sharing the message is a good option. If they put in the effort, we will listen to them.

**R7:** They should visit homes; that’s the best option. In my opinion, some people have phones while others don’t. If you organize a camp, some women might be busy with household chores and won’t be able to go. It’s better to go house to house.

**Interviewer:** We’ve heard that visiting homes sometimes upsets people. How does your community react to this?

**R7:** Only a few people are like that, very few. For example, when we asked some ladies to join, they refused because their elders didn’t allow them. They weren’t sure why we were taking them somewhere. Some people are strict, but it’s less common these days. My husband allowed me to go.

**Interviewer:** In your homes, do your husbands have the final say?

**Mostly:** Yes, the husbands do.

**R1:** Hamari

**Interviewer:** And now, what jobs do your husbands do?

**R4:** They go to work—at a gardening company. Some drive rickshaws, and they’re the ones in charge.

**R2:** They go fishing—household decisions are mostly made by the family members.

**Interviewer:** Can you explain a bit about the challenges you face in receiving health-related information or messages? What issues arise when trying to deliver health information to you?

**R3:** The men in the house don’t understand or like it. They don’t get why someone has come or what they want to share.

**R1:** If there are camps, not all women can leave their homes at the same time.

**Interviewer:** Is language a barrier?

**R1**: Everyone understands Urdu, including Pathans and Sindhis. However, Urdu speakers don’t understand Punjabi or Balochi.

**Interviewer:** How bad is the electricity shortage in your area?

**R3:** It’s very severe.

**Interviewer:** If we were to say that health messages should be delivered through TV, at what time is electricity typically available in your area, and do you watch TV?

**R8:** We don’t have a TV at home; there’s a mobile phone with my younger sister.

**R3:** My husband watches news on BOL every morning at 7 a.m. We also use USBs, but the best method is if someone comes home to explain any message.

**R5:** In our house, the news is on every morning as well. They watch BOL, and I catch a little bit of it. By 7:30 a.m., he leaves for work.

**R6:** We have both TV and mobile phones, but I don’t watch TV.

**Interviewer:** How many of you have your own phones?

R6 has her own phone.

R1 has her own phone.

R7 has a basic phone.

Two women’s husbands have basic phones.

One person’s sister has a phone.

**Interviewer:** Can you tell us which digital platforms women in your area use to get health-related information? You’ve mentioned TV and mobile. Do you or others watch YouTube, for instance? On YouTube, do you watch anything specific?

**R2:** People watch news on YouTube. Husbands watch cricket.

**R4:** TikTok is also used. My husband makes videos with our daughter, and they have their own channel.

**R1:** Most people watch TV. Mobile phones are not allowed in most households, and only a few people are permitted to use them. The best method is through TV because every woman and child can watch it, and everyone has access. There should be a dedicated channel focused on health-related topics, where you can share information.

**Interviewer:** Is there any local channel that is commonly watched? We've heard of one called "Sindhi."

**R1:** It’s not called "Sindhi." There’s a channel named Saman, which we watch.

**Interviewer:** How many people watch TV?

**R8:** Many people watch the news. It would be ideal to have health-related information on the same channel. Husbands turn on the TV in the morning before going to work, so many people would see it. In the evening, news is also watched. Morning and evening are the times when news is primarily on.

**R8, R4, and R1** said that TV is commonly used in homes, and providing information through channels would be the best method.

**R3:** There’s no TV at home, so we use mobile phones instead.

**Interviewer:** What time do you usually watch the news?

**R8:** Morning and evening—especially around 7 or 8 p.m., when husbands and fathers come home. The 9 p.m. news is also watched by many.

**Interviewer:** For those who don’t use TV or mobile phones, how can messages reach them?

**R4:** By going to their homes.

**Interviewer:** What about organizing camps?

**R4:** That’s possible.

**R8:** No, not everyone gets permission to go to camps.

**R2:** I can attend since my husband doesn’t object.

**R5:** If we talk about challenges, women often inquire about their child’s health. For example, if a husband’s job or income is insufficient, they expect something to be provided for health purposes because expenses are very high.

**R1:** It’s not just about information. You need to give something for free along with it. That’s when people will follow through because incomes are so low nowadays. If someone pays rent and covers household expenses, the 20,000–25,000 salary is completely used up. That’s why not everyone can afford things.

**Interviewer:** Can you tell me about the role of community leaders in spreading health-related messages? For example, social workers or those running NGOs—do they play an important role?

**R4:** My mother-in-law is a polio supervisor. If there’s ever an issue, she comes by, goes door to door, and keeps me informed about everything in the neighborhood—what medicines to take, which diseases are spreading, what vaccines to get, how to manage children’s diarrhea, etc.

**R8:** Polio workers come, Aga Khan workers also visit, and Sindh workers come as well.

**Interviewer:** Do you listen to them?

**R8:** Yes, we listen to them. They come regardless of time and explain things to us, which benefits us. But there are some people who don’t listen. They drive them away and spread rumors like “polio workers give wrong medicine,” etc. In our neighborhood, the workers are local. We know them—they live here and work in institutions, so we don’t drive them away.

**R7:** There have been thefts too. People have come asking for water and then stolen money, so people are cautious. I only allow people I know to enter. This kind of theft has happened twice.

**Interviewer:** What do you think about the role of community leaders?

**R3:** Polio workers visit our house.

**Interviewer:** If they were given a message to share, would they inform you?

**R3:** Yes, they definitely would.

**Interviewer:** Are there any specific health issues or topics you think should be discussed or highlighted for women with children under five? For example, posters, camps, or home visits?

**R4:** The most important topic is children’s nutrition. Considering how they are growing, what should we be feeding them? For example, I’m giving my child porridge, but now they’re refusing to eat it. What should I give instead? Sometimes, in the morning, I wonder what to feed the children. Whenever doctors come to the house or the center, I always ask this question.

**Interviewer:** What do you think is an important message? Is there a specific topic that should be discussed, and how should messages be delivered?

**R1:** How to manage at home and care for a child. We are often worried about what to feed children and how to manage them. What is right and what is wrong? Many mothers lack this knowledge. New mothers, in particular, know nothing—they are like a blank slate and sometimes make mistakes with their children. We should at least know what to feed and how to care for them.

**R7:** We mostly ask our elders, and we do what they suggest.

**Interviewer:** Who are the elders in your household?

**R4:** My mother.

**R3:** My mother-in-law.

**R1 and R6:** We live separately but occasionally talk to our mothers-in-law on the phone, and we listen to their advice.

**R5:** My in-laws have passed away. My husband drives a Suzuki, so when he goes to work, all the household responsibilities fall on me. I manage everything on my own.

**R7:** When my daughter gets sick and I bring her here with a fever, they usually give Panadol or some syrup. Earlier, when there was weakness, they used to give tonic for children, but now there’s nothing like that available. Previously, there was even a transportation service. For example, I had an appointment on the 2nd of the month, but I couldn’t go because my husband’s job was on hold, and the rickshaw fare is 500 rupees each way. We didn’t have enough money, so my husband said to wait until his salary arrived. It became a big issue.

**R8:** Sometimes, when alone, I wonder who to go with. When husbands take leave, there’s job insecurity.

**R1:** Here, assistance is provided to some people but not to everyone. For example, only those with a “yellow file” receive help. Now, even that has reduced, and most assistance is limited to pregnant women.

**Interviewer:** Are there other important topics, such as pregnancy or other issues concerning women?

**R3:** Using fruits should be encouraged as it improves health.

**R6:** Access to clean water is crucial for cleanliness. Nowadays, water is highly contaminated. It’s important to emphasize that water should be clean, boiled, and safe to drink. For example, we boil water for the children, but we don’t drink it ourselves. If we fall sick, the children who stay with us also get sick. After all, how long can a person rely on filtered water? Filters don’t last for so long, and then we have to buy a new one.

**Interviewer:** My final question—do you want to share anything else related to health that we haven’t asked about yet?

**R4:** The issues are general, like back pain during pregnancy. I experienced it recently, but it wasn’t an emergency, so I didn’t go for treatment. What should we do about such issues?

**R5:** Look at the rising costs of living. Women only think about giving their children good food while they just eat enough to fill their stomachs. Nowadays, even 500 rupees has no value.

**Interviewer:** Alright, thank you all very much for taking the time to speak with us.

**FGD 3**

Assalam-o-Alaikum, I am Sameeta, and this is Hajrah. We are from AKU. As we explained earlier, I will repeat for recording purposes. Today, we will have a conversation with your permission, focusing on health-related topics. This will include discussing how to effectively deliver health messages to your community and the people around you. We will also record this conversation so that we can analyze it later.

Shall we begin? Do you have any questions before we start?

For example, we might talk about child hygiene or how to maintain a clean environment around them.

In your community, if we wanted to deliver a health message—like introducing a new vaccine for children, explaining its benefits, or suggesting what kind of food children should eat—how should we convey such messages to you effectively?

This discussion will help us understand and communicate better.

Please mention your name when you speak. Your voice will be recorded, but there will be no video.

**Interviewer:** What do you do in your free time?

**R7**: Pray (Namaz).

**R4:** Recite the Quran.

**R3:** Read books.

**Interviewer:** What kind of books do you read, R3?

**R3:** Islamic books.

**Interviewer:** How important do you think health is in your community? In your opinion, if you and those around you maintain cleanliness, how much can it reduce the spread of diseases?

**R1:** Some people clean their homes but neglect the cleanliness of their surroundings, such as streets. They just focus on their houses and throw trash in front of their streets, which is wrong. People should keep their homes clean but also maintain the cleanliness of their surroundings. Trash shouldn’t be thrown around carelessly; it should be disposed of properly in one place instead of being scattered. So, the house will remain clean, the surroundings will stay clean, and diseases will spread less.

**R3:** Yes, cleanliness significantly reduces mosquitoes and flies. In places where there is cleanliness, you won’t find flies or mosquitoes. Like she mentioned, cleanliness should be such that both the house and the surroundings are clean. It shouldn’t be that you clean your house and then throw trash outside. Throwing trash outside pollutes the environment, and the flies and mosquitoes from outside enter our rooms through windows and doors, making children very sick.

**R4:** Children always fall ill in dirty environments. The dirtier the surroundings, the sicker children remain. Cleanliness is half of faith and very important.

**Interviewer:** Do you use any common resources in your community that you trust to provide reliable health-related information?

**R2:** Community health workers come to our homes.

**Interviewer:** What do they talk about?

**R2:** They share health-related information. For example, during their visits, they discuss health topics, including family planning.

**Interviewer:** How often do they visit?

**R2:** Every three months.
Every 15–20 days.

**R1:** Health workers who have been coming for years are very familiar to us—some have been visiting for 15 years. There's a level of closeness and trust, almost like family or friends. We can easily share things with them, and they listen to us as much as we listen to them. It’s a friendly and comfortable environment.

**R8:** Aga Khan health workers also visit, and we listen to their advice too.

**Interviewer:** Apart from this, do you use TV, newspapers, radio, or apps like TikTok for health information?

**R3:** I watch TV, usually in the mornings, on Geo Kahani.

**Interviewer:** Does Geo Kahani broadcast anything related to health?

**R3:** No.

**R5:** We mostly use WhatsApp. Apps like TikTok are spreading indecency and shouldn’t exist. There should be limits to how these platforms are used. Instead, they are showing inappropriate content, which is wrong. Kids use mobile phones, watch these things, and it spoils the environment. Children also get influenced, and the atmosphere becomes worse.

**Interviewer:** So, on WhatsApp, what do you prefer: written messages, voice messages, or phone calls?

**R5:** Voice messages are the best option.

**Interviewer:** What about others?

**R2:** In this community, there are mostly Pashtun families. Many of them don’t have mobile phones, so we need to go door-to-door to talk to them. If we visit their homes or hospitals, their entire family will listen to the message together. Afterward, people in the neighborhood will spread the message by saying, "Such and such a team came to our house, and they shared this message with us. Now we should follow this advice." So, it’s better to conduct counseling door-to-door instead of relying solely on mobile phones.

**Interviewer:** What do you think is the best way to reach you? For example, if we want to deliver a message, how should we do it?

**R8:** A team should come to our homes and share information. Many households have no knowledge of these things. They are simple people living traditional lives. They don’t know about cleanliness or the impact of the garbage spreading around them. The environment is deteriorating. So, if a team visits, they can explain things properly and get our attention by saying, ", if you focus on this and do it, things will improve for you."

**Interviewer:** How many of you have your own phones?

**R1:** I have one.
**R4:** I have one.
**R5 and R7:** There’s a small phone at home, but it just stays there.

**Interviewer:** Apart from this, considering things like healthcare clinics, campaigns, or social media platforms, which method do you think is the most effective?

**R8:** Campaigns are the best way because they reach everyone. However, door-to-door awareness is important too. Not everyone can attend a camp. At home, people can listen to the complete information, but at a camp, they may only hear part of it. For instance, if I hear something at a camp and later share it with a friend, they will learn it second-hand. Camps are beneficial, but not everyone attends, and sometimes the full message isn’t conveyed. Door-to-door campaigns ensure people hear the whole message and can share it further.

**R2:** Door-to-door communication seems most appropriate; that’s the best method.

**Interviewer:** Can you explain the difficulties or challenges you face in accessing health-related information or messages?

**R8:** Yes, absolutely. When we are sick, we go to the doctor.

**Interviewer:** Regarding activities or health-related messages, what kinds of issues arise? For example, among lady health workers, do you face challenges communicating or delivering messages, such as language barriers?

**R8:** For instance, if someone speaks Pashto, they may not understand Urdu. Similarly, if someone speaks Sindhi, they won’t understand Pashto. In such cases, there should be someone in the environment who speaks their language, making it easier for them to understand.

**R3:** The Aga Khan representatives who come to us are really good. They share and explain everything thoroughly, including details about health camps. What makes them even better is that they provide transportation services. They pick us up in their vehicles and drop us back, which is very convenient. For example, if someone needs help during delivery, we can call them, and they respond immediately. Their services are the best in this area.

**R7:** I hold gatherings by going door-to-door.

**Interviewer:** So if we provide information this way, do you think the message will reach people?

**R7:** Yes, absolutely, it will reach them.

**R8:** For example, if there are camps, not everyone can attend them. The Aga Khan facility can provide us with more support. As for the internet, some people use it while others don’t. Sometimes there’s electricity, and sometimes there isn’t. Not everyone has an internet package. However, direct communication makes the message clearer, and people realize it is important.

**Interviewer:** Can you tell us which digital platforms women in your area use to access health information? For instance, mobile phones or TV? What is most commonly available in every household for health purposes?

**R8:** Most people don’t use mobile phones for health information.

**R5:** They don’t use them much. But since I’m quite weak, I do search on mobile or the internet for information, even for children.

**Interviewer:** Which app do you use for this?

**R5:** I use YouTube mostly. Other people use this as well.

**Interviewer:** Besides that, is there anything else people use?

**R5:** A little bit, yes—like TV or mobile phones. These provide information as well. Additionally, the Aga Khan team visits households every 10–15 days. They inform us, administer vaccines to children, and respond on time. This method is much better.

**Interviewer:** Does anyone here use the radio?

**R3:** No.

**Interviewer:** Alright. For those who mentioned digital platforms, TV, and mobile phones, what are their benefits?

**R5:** They do have benefits, but we don’t get much time to use them. When someone visits us, it’s important to listen to them because they have taken time out of their day to come. We only use TV or mobile phones when we have time, and sometimes we don’t have time for days.

**Interviewer:** How frequently do you use your phone?

**R5:** Very rarely, usually only at night. I’m busy all day.

**Interviewer:** The next question is about any social or cultural norms that make it difficult to convey health-related messages.

**R8:** For example, in male-dominated households, where there is only one phone, it creates challenges. If there’s one phone in a big family, the men—like the father or brothers—have it. The messages don’t reach the women. That’s why door-to-door counseling is more effective. This way, the message can be clearly conveyed and understood.

**Interviewer:** Some people mentioned that you need to take permission from the male head of the household before sharing a message. Is that true?

**R8:** Yes, that’s true. Around here, people say, “First talk to our father-in-law or husband, get their permission, and then you can enter the house.” Many people have this mindset.

**Interviewer:** Anything else?

**R3:** Some people say that the head of their household is their father-in-law, and until he makes a decision, they cannot move forward. This is something present in the community.

**Interviewer:** Do you think health is affected by this? For example, if the male head of the household refuses something related to health?

**R3:** Yes, we can counsel them by explaining that this is related to the health of children and mothers.

**Interviewer:** Anything else you would like to share?

**R7:** Seeking permission is essential. Otherwise, many issues arise later, like, “Why did you go out without asking for permission?” They don’t realize that behind all this, our children’s health is deteriorating. But we insist and tell them, “Look, our children are getting sick. These people are coming for our safety and working hard for us, so it’s necessary for them to come, and for us to accompany them.”

**Interviewer:** With all these challenges, how do you think we can help you? For example, if we try to deliver messages, what can we do to convince the male heads of households? Should we set up camps and invite them as well?

**R8:** No, they won’t come, and then the women won’t come either. The easiest method is door-to-door counseling; that will work better.

**R1:** They tell us, “Go ahead. We’ve entrusted the children to you; you women handle it yourselves.” The mothers understand because they are directly involved. If the father were home, he might understand, but he leaves in the morning and comes back at night. What will he comprehend? So, we say that if there is a need, we’ll inform the husband. My husband secretly gave me permission because he knows our children are often sick, I have health issues, and that’s why the children are unwell too. For instance, my child has a fever, I am breastfeeding, and now the child also gets sick. I sought his permission to come here, but the rest of the family—brothers-in-law, sisters-in-law, and mother-in-law—takes time to agree.

**Interviewer:** Do you all think community leaders play a role in spreading health-related messages?

**R3:** Yes, they do. That’s why our society has been able to survive so well. For many years now, the Aga Khan ladies have been visiting us, educating us about everything, and resolving many health issues related to our children. Whether it’s administering vaccines or anything else, they come to our homes, inform us, and because of their visits, even our mother-in-law, who had very traditional views, has changed significantly.

**Interviewer:** Apart from this, would anyone else like to share their thoughts on the role of community leaders?

**R7:** It’s all functioning because of them. If community leaders didn’t engage with people, society would regress. The community is proactive, which is why work related to health is progressing.

**R3:** In my home, it’s mostly my mother-in-law who has authority. If she says I can go somewhere, then I go; otherwise, I don’t.

**Interviewer:** Apart from the AKU (Aga Khan University) team, who else are the community leaders here?

**R2:** Lady Health Workers (LHWs) come here, apart from the Aga Khan team. These are the only two groups that provide us with information.

**Interviewer:** Is there anyone else, like religious leaders, such as a mosque imam, whose guidance people prefer to listen to?

**R8:** People do like to listen to them, like the mosque imam, but they don’t visit homes. It’s mainly the AKU team and LHWs who reach out to us at home.

**R7:** On Fridays, during the sermon, the imam delivers a speech that provides religious knowledge. Of course, we already know about our religion since we follow Islam, but on Fridays, they give us important messages and teachings. However, the Aga Khan team and health workers are unique because they come immediately when we call them for help. They care for our children’s health and provide us with many facilities and valuable information, which we’ve primarily gained from the Aga Khan services.

**Interviewer:** Are there any specific health topics or issues you feel are particularly important for women living in underprivileged areas? If so, what are they? For example, related to children’s nutrition or other matters?

**R4:** Lumps in the body—people don’t know about cancer, so awareness should be raised about it. For example, if a daughter hides something from her mother, she should be told not to hide it and to share it with her. She can also tell a doctor. Information about this should be provided. Similarly, there should be awareness about menstruation. Young girls don’t have much understanding of this. They should be taught how to change a pad and provided with proper information. Many people face problems like infections, so they should be educated on how to prevent such issues.

**Interviewer:** Anything else?

**R2:** Regarding menstruation, awareness should be raised, for example, when ladies visit, their mothers should be informed about the importance of hygiene. Not maintaining hygiene can cause infections and make girls ill. Such topics should be discussed, and information should be provided. When ladies come and sit together to listen, they enjoy listening and are more receptive.

**R1:** If such discussions are held among women, the message will spread widely and be beneficial. For example, you should first ask me questions. When I come to you, you should ask about my health, and I will respond. Similarly, I will listen to you, and you will listen to me. In this way, we will exchange questions and answers, which will benefit both sides. I will learn from you, and when women feel heard, they become happy and more open to communication.

**Interviewer:** Currently, our focus is to spread messages digitally or through other mediums. Can you suggest how we can convey such messages effectively?

**R3:** No, these messages don’t reach women effectively. Besides, there’s a prevalent issue nowadays where pads are discarded openly, which creates an unhygienic environment. Children passing by don’t know what these are and might unknowingly play with them. There should be awareness about maintaining cleanliness and proper disposal of such things.

**Interviewer:** Do you think people won’t understand such messages through digital mediums?

**R8:** People don’t pay attention to such messages and tend to ignore them. Face-to-face communication works better, as it allows for proper understanding and effective counseling.

**R2:** Face-to-face interactions make things clearer and counseling more effective.

**R6:** If I have any issues, I make excuses to my mother and visit Aga Khan (health services).

**Interviewer:** Is there anything else about health information that you feel we haven’t discussed but should be addressed?

**R7:** Yes, about the first set of vaccines. These initial vaccinations are very good for children. However, many mothers refuse them because of concerns about their children experiencing pain or fever. They should be counseled that this is normal and these vaccines are not harmful. Most refusals come from Pathan families, who say, *“My child was in a lot of pain and got very sick.”* Awareness about the importance of these vaccines is needed.

**Interviewer:** Would anyone else like to add something?

**R5:** For breastfeeding mothers, especially those who’ve had C-sections, there should be special focus. After surgery, women become very weak. Although women with normal deliveries recover better, those who undergo surgery struggle more. For instance, lifting the child causes back pain. There should be initiatives to address this.

**R3:** Information on increasing breast milk should also be shared. Many women here quickly switch their babies to formula whenever they cry a little. Awareness on how to increase milk production should be provided.

**Interviewer:** Understood... Thank you all very much.

**FGD 4**

**Interviewer:** Assalam-o-Alaikum, I’m Sameeta, and this is Hajrah. We are from AKU.

As mentioned earlier, let me reiterate: with your permission, we will have a conversation today. It will focus on health and how health messages can be delivered effectively within your community and to the people around you. Shall we begin? Do you have any questions before we start?

**Participants:** No.

**Interviewer:** First of all, please tell me what you like to do in your free time. How do you spend your leisure time?

**R2:** I do stitching.

**R7:** Stitching.

**R5:** Stitching.

**Interviewer:** Apart from that, do you use phones or watch TV?

**Mostly:** No.

**Interviewer:** My next question is, in your opinion, how important is health within your community? Do you think keeping yourself and your surroundings clean can reduce the spread of common illnesses? Please share how significant health is for you. How important do you think cleanliness is in maintaining hygiene, and what role does it play in your surroundings? Do you believe that by maintaining cleanliness, diseases can be reduced?

**R7:** It’s very important. Diseases are reduced. I keep my home clean.

**Interviewer:** Can you name some common resources within your community that help you maintain good health? For instance, how do you receive important announcements or health-related information?

**R5:** Aga Khan Centre.

**Interviewer:** Who provides this information?

**R5:** They give medicine for children who have a fever, and I take the same medicine from there. We have to come here to get the medicine.

**Interviewer:** Are there any other sources nearby?

**R1:** We get medicine from the government hospital as well; we have to go there.

**Interviewer:** When you’re at home, how do you find out what to feed your children or how to take care of yourself? Is there any method?

**R1:** Yes, yes, we manage. Whenever someone gets sick, we clean our house more often, bring medicine, go to the hospital, and get the required treatment.

**Interviewer:** Do you ever use your mobile or TV at home to gain information?

**Everyone:** No.

**Interviewer:** Alright, how many of you have phones?

**R8:** We have basic keypad phones.

**Interviewer:** What do you use them for?

**R8:** To talk to brothers, sisters, and relatives.

**Interviewer:** Okay, can you describe the best way to spread health-related messages or information in your community? Since you don’t use phones much or digital platforms, what would be the most effective way?

**R8:** Health comes from praying, reading the Quran, and connecting with Allah. Good health also comes from eating and drinking properly.

**Interviewer:** So, if we need to send you a message, how should we do it?

**R8:** Messages could work.

**Interviewer:** Do you have personal phone?

**R8:** Yes.

**Interviewer:** What SIM cards are mostly used?

**R8:** Zong is the most commonly used.

**Interviewer:** What about others? As doctors, we learn new things in our field. How do you think we could convey that information to you? For example, we use the internet to educate people on what should be done and how to do it. How would you prefer to learn about such things?

**R7:** We watch TV, vlogs, and YouTube.

**Interviewer:** Are there any local vloggers from this area?

**Participants:** No.

**Interviewer:** When the people from Aga Khan Centre come to your house, can you tell us about them? Do they help you? Is their method good or not?
**R5:** It's a good method. They take the children with them, give them vaccines right there, and provide information. They tell us to keep everything clean and feed the children properly.
**Interviewer:** When they say these things, do they reach you?
Yes, they deliver the message. Whoever needs help, they come and inform us. They tell us to stay healthy and visit the hospital if needed. Sometimes, they come once a month or every two months.

**Interviewer**: And others, can you share what kind of help you receive?
**R2:** They take us in a vehicle, get the children vaccinated, and then drop us back home. It’s a very convenient service. They also remind us to take care of hygiene and cleanliness.

**Interviewer:** Can you elaborate on any challenges or difficulties you face in accessing health-related information or messages? What difficulties do you face regarding health information? If you have a question, what is the problem? Do you feel comfortable asking about health-related concerns? Do you face any issues asking someone about them?

For example, at home, if you don’t feel like asking, what kind of problems do you encounter? Do you need to call someone? Is there an issue like not getting a signal or not having a phone? Or perhaps there’s no transportation to reach the community center?
What kinds of problems like this do you face? Please share.

**R2:** Yes, if the children are sick, we take them ourselves.

**Interviewer:** That’s fine, but what difficulties do you encounter?
**R1:** When the children are sick, we go, get the medicine, and come back.

**Interviewer:** Is transportation easily available here?
**R1:** No, there’s no vehicle available to go to the hospital. We go to the government hospital, and we have to walk there. At the community center here, medicines are available for children aged 4 to 5 years, but not for older children. The government hospital is far from our house—it takes about half an hour to reach. It’s under the Sindh government, and we walk there.

**Interviewer:** Do you face any issues understanding the language of the Lady Health Workers (LHWs)?
**R1:** No, we understand Urdu, and there’s no problem with the language.

**Interviewer:** Some of you mentioned that you use TV and phones frequently, but do you face electricity issues?
**R7:** Yes, we do. The electricity comes for two hours and then goes off for three hours. Because of this, we sometimes can’t receive messages.

**Interviewer:** What about the rest of the people?
**R3:** Whenever it’s necessary, someone comes and informs us. But sometimes, we also get information through TV or phones.

**Interviewer:** Alright, my last question is: What digital platforms do you use to get health-related information? Do you use phones or WhatsApp, etc.?
**R7:** Yes, we use phones a lot, even the children have them.

**Interviewer:** Do the rest of the people have phones, or do their husbands have phones?

**R1:** No, they don’t.

**Interviewer**: Do you use TV or radio?
**R2:** No, we don’t. We’re not interested in them.

**Interviewer:** What are the benefits and challenges of mobile phones and TVs?
**R8:** Mobile phones are very helpful. When we face difficulties, they help us find solutions.

**Interviewer:** How many times a day do you use a mobile phone?
**R8:** I use it at least twice a day—my older sister calls in the morning, and I use it again later in the day.

**Interviewer:** Do you have internet access?
**R1:** We don’t have touch-screen phones or Wi-Fi. In our household, only three people have phones, and they are the property owners.

**Interviewer:** Can you mention any cultural or social issues in your community that make it difficult for you to understand health-related messages? Are there any community rules or principles that create barriers?
**R8:** No, there are no such issues.

**Interviewer:** For example, some people we talked to said they don't get vaccinated because their elders forbid it.
**R2:** Both of my children have been vaccinated.

**Interviewer:** Are there any community rules here that people have to follow, like doing what others say?
**R7:** I got two vaccines for my child, but then I stopped.

**Interviewer:** Why did you stop?
**R7:** The child got a fever for 2-3 days after the vaccines, so I didn’t get more.

**Interviewer:** In households where men dominate decision-making, can this create challenges in delivering health messages effectively?
**R6:** No—men usually say, "It’s your own matter."

**Interviewer: In your homes, who has more authority, men or women?**
**Mostly:** Both.

**Interviewer: Do you think community leaders play an important role in delivering health-related messages? For example, in mosques, some people listen to the Maulana (religious leader). There are also elders, midwives, or other influential figures whom people trust and follow. Is there someone like this in your community?**

**R4:** Zakriya.

**Interviewer: What does he do?**
**R4:** He’s a counselor.

**Interviewer: What does he help you with?**
**R1:** He works on issues like clearing sewer drains and arranging cleanups.

**Interviewer: Anyone else?**
**R5:** In our area, there’s also a *Jamot* (local worker) who ensures cleanliness.

**Interviewer: If a message is sent to them, can they pass it on to you?**
**R5:** No, they won’t pass it on again. They are very busy and don’t follow up.

**Interviewer: So, they help resolve issues but don’t deliver messages?**
**R1:** Yes, they help when needed, like with water problems.

**Interviewer: You go to their office whenever there’s work to be done?**
**R1:** Yes.

**Interviewer: Okay, my next question is: Are there any specific health topics or issues that you feel are particularly important for women, ones that should be communicated to you or discussed with you?** **Is there anything about children or yourself that you feel you need more information on? Are there women’s health issues you feel are not being addressed or communicated to you? Would you prefer these topics to be shown to you on a screen or through other means? Is there anything missing when it comes to health-related communication?**

**R3:** No, it’s fine. Things happen as they do, and it works.

**Interviewer: So, you don’t feel there’s a problem with health messages not reaching you?**
**R3:** The people who come to our homes tell us, and we trust what they say.

**Interviewer: Are there any specific topics or areas related to health that you feel should be communicated to you, like child health or vaccinations?**
**When the women who visit us discuss health, is there anything they don’t talk about?**

**R6:** No, we ask them whatever we need to, and they explain everything.

**Interviewer: What kind of important topics do they talk to you about?**
**R6:** They guide us about children’s health, our own health, and treatments.

**Interviewer: Anything else? Any other important topic they discuss?**
**R5:** They tell us about everything—what’s right and what’s not.

**Interviewer: Do you feel this is helpful?**
**R5:** Yes, they tell everyone.

**Interviewer: Apart from this, is there any other health-related issue that I haven’t asked about or any topic you’d like to mention?**
**R1:** Health mostly comes down to eating well, waking up on time, and sleeping on time.

**Interviewer: Would anyone else like to share anything on this topic?**
**Everyone:** No.

**Thank you so much.**

**FGD 5**

**Assalam-o-Alaikum. I am Sameeta, and this is Hajrah. We are both from AKU.**
As I mentioned earlier, for the purpose of this recording, I will repeat myself. With your permission, we will have a conversation today, which will focus on health-related matters. Specifically, how health messages can be communicated within your community and among those around you. Shall we begin? Do you have any questions before we start?

**R:** No.

**Interviewer:** Alright, first, I’d like to ask what you do in your free time? What do you usually do at home? Please share.

**R3:** I work and take care of the children.

**Interviewer:** I see. And what about you? What do you like to do in your free time at home?

**R:** I just sit and relax.

**Interviewer:** Does anyone do anything else, like stitching or something similar?

**R2:** I do stitching sometimes when I have free time. I sew my own clothes.

**Interviewer:** That’s good. And you?

**R6:** I also do stitching.

**Interviewer:** What do you all think about how important health is in your community? When it comes to cleanliness, health, and overall well-being, would you say you keep your surroundings clean? Does this help reduce common illnesses? Could you share your thoughts on this?

**R3:** I clean the entire house, keep the children clean, and also maintain my own cleanliness.

**Interviewer:** And how important do you think these things are for health?

**R3:** I mean, if I keep everything clean and tidy, it will keep illnesses away.

**Interviewer:** That’s right. Would anyone else like to add something? What do you usually do at home?

**R5:** I clean as well, and of course, it makes a difference in reducing illnesses in the surroundings.

**Interviewer:** Does anyone else have something to share?

**R7:** Cleanliness is half of faith. It is something we are supposed to maintain. By keeping things clean, it naturally impacts everything, including the prevalence of illnesses.

**Interviewer:** Alright. Apart from this, are there common sources of information in your community that you use, especially related to health? If you need to know something or ask about a health-related matter, where do you go? What is your usual way of finding out?

**R:** I don’t understand what you mean.

**Interviewer:** If you have health-related questions, what do you usually do? Where do you get answers from?

**R:** We consult a doctor.

**Interviewer:** And where do you usually go?

**R2:** To the doctor. There’s a private clinic, a family clinic, and we go there. We ask them for advice, whether it’s about food or anything else.

**R:** Yes, you consult a doctor.

**Interviewer:** Alright. Could you tell me how far the private clinic is from your home?

**R2:** It’s not very far from our home. It’s quite nearby.

**Interviewer:** Nearby, as in you can go there by bike? You can’t walk there?

**R2:** Yes, we go by bike, not on foot.

**Interviewer:** And how much do they charge there?

**R2:** The charges are quite high since it’s a private clinic.

**Interviewer:** But you still prefer to go to the doctor?

**R2:** Yes, we prefer to consult the doctor.

**Interviewer:** And anyone else? Do you go to someone else for advice?

**R4:** We ask my mother-in-law. She lives with us and gives advice based on her experience.

**Interviewer:** Apart from that, do you use any other sources like the radio, newspaper, TV, or WhatsApp?

**R3:** No, we don’t use those.

**Interviewer:** Alright, you mentioned earlier that you have a phone. What do you usually do on it?

**R5:** I just play games, nothing else.

**Interviewer:** Besides games, do you use it for anything else, like WhatsApp, Facebook, or TikTok?

**R5:** I watch TikTok and talk to my parents and sister on the phone.

**Interviewer:** And if you have health-related questions, especially about your children, whom do you ask?

**R5:** I ask my mother-in-law or my own mother.

**Interviewer:** Do you live with your mother-in-law?

**R5:** Yes.

**Interviewer:** And do you use your mobile phone to look up health-related information on WhatsApp, YouTube, or anything like that?

**R5:** No, not much. I don’t use my mobile for health-related purposes.

**Interviewer:** Alright, and you mentioned earlier that you use WhatsApp, right?

**R2:** Yes, I use WhatsApp. I also read digests on phone and watch YouTube.

**Interviewer:** What do you watch on YouTube?

**R2:** Mostly cooking-related content.

**Interviewer:** Do you ever watch anything about health on YouTube?

**R2:** Sometimes, especially about children—what’s beneficial for them and what’s not.

**Interviewer:** And what about you? Whom do you consult if you have a health-related question?

**R:** I usually figure it out myself because I don’t have a mother-in-law or father-in-law anymore.

**Interviewer:** So it’s just your husband and your children?

**R:** Yes.

**Interviewer:** Who makes the household decisions, then?

**R:** My husband manages everything, but I look after the children. If they’re sick, I take them to the doctor.

**Interviewer:** How many of you have a TV at home?

**R:** I have one.

**R:** I have one too. (Two Rs mentioned having a TV.)

**Interviewer:** What do you all watch on TV?

**R1:** We don’t have a TV in our room. It’s in my father-in-law’s room, so they are the ones who watch it. The kids, like my sister-in-law’s children, watch cartoons and such.

**R5:** At our house, only the small children watch TV. I don’t watch it myself because I’m usually busy with work and don’t have the time.

**Interviewer:** Alright, R1, since your mother-in-law has passed away, who do you consult or discuss things with regarding your children?

**R5:** I discuss these matters with my sister-in-law.

**Interviewer:** If I were to ask how health-related messages or information could be shared in your community—whether about cleanliness, children’s health, vaccinations, or maternal health—what do you think is the best way to reach you?

**R3:** You could call us to the clinic to inform us, or you could come directly to our homes and tell us.

**Interviewer:** You mentioned that someone comes to your house. Does that happen regularly?

**R3:** Yes, someone does visit.

**Interviewer:** And do you feel that these are both good ways to convey information?

**R3:** Yes, they are.

**Interviewer:** Between the two, which do you think is the best method?

**R3:** If you come to our homes, that’s very convenient because we don’t always have the time. If you don’t come, we are left with no choice but to go to the clinic ourselves.

**Interviewer:** How often does a worker visit your area?

**R3:** Usually once a week, though sometimes it’s once a month.

**Interviewer:** What do you ask them, and what do they tell you?

**R3:** They mostly visit when someone is pregnant. Otherwise, their visits are less frequent.

**Interviewer:** Is there anything else you’d like to share? What method do you find most effective for health-related messages?

**R3:** I’m not sure.

**Interviewer:** The purpose of this conversation is to understand how we can deliver health messages to your community. For example, if there’s new information about children’s health, how can we ensure it reaches you? Should we use phones, TV, home visits, or posters? Which method would you prefer?

**R6:** Visiting homes to inform us is a great option since we don’t have much free time.

**R4:** We don’t have a TV at home. My husband has a small phone, and we have a small tape recorder that the children or my father-in-law use to listen to things. My father-in-law is unwell; he has heart disease and has had a stroke, so he can’t walk. My husband also has mobility issues because of polio. We all live together, including my brothers-in-law and sisters-in-law.

**Interviewer:** Does your husband ever get calls from the center on his phone?

**R4:** Yes, we do receive calls from the center occasionally on my husband’s phone. They inquire about the health of both the mother and the child.

**Interviewer:** Does a team visit your home as well?

**R4:** Yes, they do. Two women come and ask about the children’s health, how they are doing, and whether they are eating properly. If there’s an issue, I also take the children to the center because they provide good treatment there.

**Interviewer:** Apart from this, does anyone else come?

**R4:** Yes, the polio vaccination worker also comes. I’ve ensured all my children receive the drops.

**Interviewer:** Can you tell me how often they come?

**R3:** Usually, they come once a month.

**Interviewer:** Others, please share how often the Aga Khan Center staff visit your homes. Do other women also come?

**R3:** Yes, the polio workers come to our area about once a month. Additionally, some other women visit to check the weight of newborns.

**Interviewer:** If you have questions for them, especially regarding children’s health, do you find their visits helpful?

**R4:** Yes, they advise us about maintaining cleanliness for the children, and we follow their guidance.

**Interviewer:** Could you elaborate on any difficulties or challenges you face in accessing health-related messages? For instance, if you want to ask something but cannot, what are the issues that prevent you from asking?

**R:** I don’t understand.

**Interviewer:** Challenges such as not having a phone or electricity—what kind of problems do you encounter that prevent you from taking your child to the doctor or asking questions?

**R7:** Yes, sometimes it happens, especially when I’m alone. I have to manage both the household chores and the child, so it becomes difficult.

**Interviewer:** When this happens, how do you manage the child’s care? Whom do you consult for advice on how to take care of them?

**R7:** I ask my sisters.

**Interviewer:** Where are they, and what do they do?

**R7:** They’re at home. But if the child’s condition still doesn’t improve, I take them to the doctor.

**Interviewer:** How far is the doctor from your community?

**R7:** It’s a bit far—if we go on foot, it’s about a 20-25 minute walk.

**Interviewer:** Apart from this, do you face any issues understanding the language of the women who visit your homes? For example, if they speak Urdu, do you have difficulty understanding them?

*(No response)*

**Interviewer:** Can you tell me which languages are spoken in your community?

**R2:** Sindhi, Punjabi, and quite a bit of Urdu.

**R:** Most people here understand Urdu.

**R4:** Yes, for instance, my mother-in-law, sister-in-law, and husband mostly understand Urdu, so the women speak with them, and they explain things to me.

**R4:** I don’t go to the doctor alone; I always go with my husband.

**R5:** I also don’t understand much Urdu, so my sister-in-law handles the conversation since she understands better.

**Interviewer:** Apart from this, how often do you get electricity in your areas?

**R5:** It comes and goes frequently. It’s available for an hour, and then it’s gone for 2-3 hours.

**R6:** In our area too, it’s available for an hour and then gone for 2 hours.

**R3:** In our area, it follows a schedule. It’s available for 1.5 hours and goes out 3-4 times a day.

**Interviewer:** Those who said they have a TV at home, what time do they usually watch it, and what do they prefer to watch?

**R2:** I don’t like TV; I prefer my mobile phone. Only the family members, like my in-laws, watch it. The children watch cartoons, and my father-in-law, since he’s religious, listens to spiritual talks.

**Interviewer:** What time do they mostly watch it?

**R2:** My father-in-law watches it in the morning and evening.

**Interviewer:** And you don’t watch it?

**R2:** No, I don’t.

**Interviewer:** You mentioned you use your phone. What do you like to do on your phone?

**R2:** Yes, I read magazines on my phone, and sometimes I use it to consult a doctor regarding my child.

**Interviewer:** Do you use any apps?

**R2:** Yes, I use different apps—TikTok and YouTube too.

**Interviewer:** Mostly TikTok? What do you watch there—anything health-related or mostly entertainment?

**R2:** No, there’s health-related content too. You can watch whatever you’re interested in.

**Interviewer:** Do you think it’s a good way to convey messages?

**R2:** Yes, yes, TikTok is a very good medium.

**Interviewer:** Are you part of any WhatsApp groups that you’ve joined?

**R2:** I used to be, but not anymore. I left them myself.

**Interviewer:** For those who said they have phones, what are your thoughts about using phones as a way to convey information? For instance, sending a video or a message?

**R5:** I don’t use my phone much, but my children watch videos on it. It keeps them quiet, but I don’t use it frequently myself.

**Interviewer:** Do the center workers call you?

**R5:** Yes, they do call. I’m the one who usually answers the calls, mostly in the morning, and I take those calls.

**Interviewer:** Can you explain any societal or cultural norms in your community that create challenges in receiving health-related messages? For example, in some areas, men have more authority, or women don’t have access to mobile phones, and only their husbands do. How do you manage communication in such situations?

**R3:** If we don’t have a phone, it becomes a problem. For instance, in case of an emergency, we have to borrow a phone from someone to make a call. Otherwise, we don’t have access to a phone. If my child is unwell, I ask someone to make the call for me. Having a phone is essential.

**Interviewer:** If your child is sick, who makes the decision about what to do?

**R3:** My husband makes the decisions. Wherever we need to go or whatever needs to be done, it’s done with his approval.

**R6:** In my house, my mother decides whether the child needs to go to the doctor or not.

**Interviewer:** What do your husbands do for work?

**R5 and R6:** They work as boat operators.

**R:** One of them works as a peon in an office.

**R2:** My husband works in a company.

**R7:** Mine does labor work.

**Interviewer:** So, in other homes, who makes the decisions?

**R:** The husband usually makes the decisions.

**R1:** My sister-in-law comes over, or I go to her.

**R2:** I mostly make the decisions myself. I don’t neglect my children and don’t listen to anyone, not even my husband. I make my own decisions because we have to ensure our children’s safety ourselves.

**Interviewer:** Does anyone else want to share? Is it a joint decision, or does one person usually take the lead? And as some of you mentioned, if your husbands are away working on boats, who makes the decisions at home?

**R5:** Then I consult my mother-in-law.

**Interviewer:** In your opinion, do community leaders play any role in spreading health-related messages? For instance, mosque imams, polio workers, or Aga Khan lady health workers—do they help convey important messages? Or is there someone in your community, like a senior or elder, whose advice everyone listens to?

**R2:** No, there’s no one like that. Everyone manages things on their own. My father-in-law is someone I listen to. In our area, there are people affiliated with political parties, and my father-in-law listens to them more than others.

**Interviewer:** What kind of information do they provide?

**R2:** If we need information about something or if there’s an issue, we consult them for details.

**Interviewer:** What kind of issues do you seek information about?

**R2:** For example, if there’s a wedding to arrange or a household problem arises, we seek their advice. However, when it comes to children’s hygiene, I handle it myself. In our household, we manage things independently.

**Interviewer:** For instance, if the mosque imam says something, do people in your community listen to him? What are your thoughts on this?

**R5:** We would listen to him, but such a situation hasn’t occurred yet.

**Interviewer:** We’re trying to understand if we ask the mosque imam to spread a message in the community, would he do it, and would people listen to him?

**R5:** We don’t have an idea about this, but if he makes an announcement, the message would likely reach our homes.

**Interviewer:** Are there any other ways to convey messages? For instance, by showing videos or putting up posters. What are your thoughts on these methods?

**R:** We don’t have phones, so videos on whatsapp don’t make sense to us. How would we watch them?

**Interviewer:** If workers come and show you a video, would you watch it?

**R:** This hasn’t happened before, but if it does, we would watch.

**Interviewer:** Do they hold camps in your area?

**R3:** Yes, there was a camp in our area. They came to administer injections to children.

**Interviewer:** What kind of injections were these?

**R3:** They didn’t explain much. They just said these were protective vaccines, and they would be given in the arm. It was the Aga Khan team that came and administered them.

**Interviewer:** Who informed you about this camp?

**R3:** They set it up near our home and called us from there, saying it was good for children’s health. So, we took our children. I was visiting my parents’ home at the time, and my in-laws told me about the camp. They also mentioned polio drops, as well as other protective vaccines.

**Interviewer:** Do you have any programs in your area?

**R:** No, nothing like that happens here.

**Interviewer:** Have you ever heard of a program where people are invited to gather and are given information?

**R:** No, nothing like that has been organized here.

**Interviewer:** Are there any specific health-related topics or issues that you feel you don’t know much about? For example, children’s hygiene or women’s health issues, where you feel you need more information to help us convey messages to your homes?

**R3:** Topics related to children and women—if you could share information, that would be helpful.

**Interviewer:** Is there any specific topic related to women’s health you’d like to know more about?

**R3:** For example, spacing between pregnancies or the use of certain capsules. Information about that would be useful.

**Interviewer:** Would anyone else like to add something?

**R2:** Please provide information about spacing between pregnancies. My in-laws don’t allow it, but I want to practice it. If we get this information, it will help us understand better.

**Interviewer:** Lastly, for those who live with their mother-in-law or sister-in-law and follow their guidance, do you have any meetings or gatherings in your area where you discuss such matters?

**R3:** No, we live in separate rooms. It’s just me and my sister-in-law, as our mother-in-law and father-in-law are no longer with us. We each stay in our own rooms.

**R:** I also stay alone with my children, and there are no meetings or gatherings.

**Interviewer:** Okay, thank you all very much.

**FGD 6**

**Assalam-o-Alaikum, my name is Sameeta, and this is Hajra. We are from AKU. As mentioned earlier, I’ll repeat for clarity: today, with your permission, we will have a discussion focused on health-related topics. Specifically, we want to understand how health messages can be communicated effectively to your community and those around you. We will also record this conversation to analyze it later.**

**Please also share your names so we know who shared which thoughts, okay? And please try to ensure everyone contributes something so that we get participation from everyone. Shall we begin? Do you have any questions before we start?**
**Everyone:** No.

**Interviewer: Alright, first, can you tell us what you do in your free time?**

**R6:** Most of the time, I look after the kids and manage household chores.
**R2:** I sew clothes, do household work, and stay busy with one thing or another.
**Interviewer: Is there anyone who engages in any other kind of activity?**

**R1:** If I get free time at night, I finish my tasks. Sometimes, I sit and make flowers using paper or computer paper—I create bouquets and frames. If someone requests something, I make it for them. For events like Milad, I prepare small flowers and other items.

**Interviewer: According to all of you, how important is health?**
**Everyone:** We don’t understand.
**Interviewer: Okay, let me ask this way: when it comes to health, how significant do you think it is?** **For example, do most people get their children vaccinated? How many people avoid letting their children play in the dirt because of the risk of diseases?**
**We’re trying to understand how aware people are about health or how much awareness needs to be created.**

**R2:** Health is very important. I don’t even let my daughter Noor Fatima sit on the floor. When I’m making bread, I keep checking to ensure she doesn’t pick up anything harmful. If I see her chewing on something, I always check her mouth with my finger to ensure it isn’t something dangerous, like dirt or stones. The children in the house play together, but I’m constantly cautious to ensure they don’t eat anything harmful.

**Interviewer: So, tell me, do the people in your area maintain cleanliness like you, or do they let their children play in the dirt?**
**R2:** No, most people let their children play in the dirt, but I don’t allow it. Who knows, they might eat something bad or develop an allergy. Many times, my child gets rashes on her face, and they start oozing fluid. Because of this, I don’t send her outside.

**Interviewer: Does anyone else want to share something about health?**

**R7:** That’s about it. People do let their children play outside, but I try my best to ensure my child plays less in the dirt.

**Interviewer: Okay, if you need any information or details in your community, how does it reach you? How is the message conveyed to you?
Mostly:** The message doesn’t really reach us.

**Interviewer: No, I mean, if there’s a message that needs to be communicated, like “this should be done,” or “this medicine is good,” or “vaccinations should be administered,” how does that message reach you? Does someone come to your door to inform you, or how does it happen?**
**R7:** Women from the Aga Khan Centre come and inform us about what needs to be done.

**Interviewer: What do they tell you?**
**R7:** They mainly talk about health, children’s diet, and vaccinations.

**Interviewer: How often do they visit?**
**R7:** Every 3–4 months.

**Interviewer: Besides the Aga Khan LHWs, does anyone else come?**
**R8:** No, only they come. Apart from them, some women come to give children polio drops.

**Interviewer: Would anyone else like to share something?**
**R5:** Yes, they come to our area, the ones who administer vaccinations and such.

**Interviewer: If you ask them any health-related questions, do they answer?
R4:** Yes, they do.

**Interviewer: Apart from this, if you want to get information, how do you do it? Do you use WhatsApp, TV, radio, or any other platform?**
**R6:** Yes, I watch YouTube on my phone, but I rarely watch TV. On YouTube, I see content about cleanliness and teach those things to my children. I also listen to what my mother-in-law advises.
**R1:** I don’t use mobile phones or TV. I just follow the advice of elders and use my own judgment because my mother is a doctor, and my grandfather was also an MBBS doctor. My mother shares a lot of information about cleanliness, so I follow her guidance.

**Interviewer: You mentioned the phone—do you not have one, or do you just not prefer using it?**
**R2:** I had a phone, but I sold it and didn’t buy another one.

**Interviewer: Who has a phone in your household? Does your husband have one?**
Almost everyone has a phone in their homes. One person doesn’t. One has their own phone, and for others, their husbands have phones.
**R3:** My brother has a mobile phone.
**Two had keypad phones, and the others in the households had touch-screen phones. One person doesn’t have one.**

**Interviewer: Like R6 mentioned using TikTok, does anyone else use TikTok or Facebook?**

**R5:** My husband uses Facebook, but I don’t. He also uses TikTok, but we don’t watch YouTube or similar platforms.

**Interviewer: If there were health-related videos, would you watch them?
R5:** Yes, definitely.

**Interviewer: How many of you have TVs at home?**
**R1:** We have an LED TV.

**Interviewer: Do you watch it?
R1:** Mostly, my father-in-law sits and watches it. Occasionally, we watch it for a little while, but often we don’t have free time due to work.

**Interviewer: Apart from R1, does anyone else have a TV at home?**
No one else has a TV.

**Interviewer: Okay, if asked, what would you say is the best way to share messages or information with the community? How can health awareness be spread effectively in your community?**

**Interviewer: Do you think phone messages can help?
R6:** Yes, they can.

**Interviewer: What kind of messages? Videos or text?**
**R6:** Both are fine.

**Interviewer: Should the messages be in Urdu or another language?**
**R6:** Both Urdu and English are fine.

**Interviewer: Can anyone else share their thoughts? What’s the best way to deliver health-related messages? For example, is it better to send people directly to houses, like lady health workers, or to set up camps?**
**R4:** We don’t have a phone at home. We just follow what my mother advises about cleanliness. She also gives instructions for children. Whatever is necessary, she tells us.
**R1:** I don’t have a phone, but my sisters do. If something important comes up, I ask them.

**Interviewer: Do you think this is helpful?
R1:** Yes, I follow their advice. If there’s another issue, I search online or consult my sisters. If I had a phone, I’d look things up myself.

**Interviewer: Are there any health campaigns in your area? Do they help?** F**or example, are there camps for polio vaccinations or vehicles for vaccination drives?
Everyone:** No, nothing like that happens.

**Interviewer: If there were, would you like that?
Everyone:** Yes, we would.

**Interviewer: Are there any such initiatives in other neighborhoods? Any health camps or similar activities?
R3:** No, no one comes except the Aga Khan vans.

**Interviewer: So, tell us, if someone falls ill in your household, what do you do? Do you go to a hospital? Where do you go?
R3:** We go to a nearby clinic.

**Interviewer: Do you have any number you can call or message in case of need?
R5:** Yes, we have an AKU number. I contact them if needed.

**Interviewer: Apart from that, are there people in your area who influence others, like influencers, social mobilizers, or the mosque imam?**

**R8:** No, we mostly just go to the hospital. But if someone’s health deteriorates a lot, we sometimes resort to traditional remedies because we feel it could be a spiritual issue.

**Interviewer: Besides children’s health, there are many other things people want to learn about. With phones now available, many people search online for answers. We’re trying to understand how we can send messages—whether from Aga Khan or elsewhere—about new developments or important practices. What’s the best way to get this information to you?
R1:** It would be better if the message comes to our home or someone visits, because there’s always an issue with mobile phones—they are not always available.

**R8:** Yes, visiting homes is better since most people don’t have mobile phones.

**Interviewer: Apart from this, is there any other important topic you would like to know about? When the Aga Khan team visits, what issues do you usually ask them about?
R4:** Mostly about children’s issues like coughs and fevers.

**Interviewer: Can you share other ways to deliver messages? For example, you all have mosques in your areas—are any health-related announcements made there?
R1:** Yes, if there’s a vaccination team visiting, the announcement is made in the mosque. Many people hear it and go there.

**Interviewer: Apart from that, have there ever been health sessions in your area where people were gathered and taught how to take care of their health?
R5:** No, we haven’t had such sessions. If they happen, it would be good. People would become aware and take it seriously. But when someone visits homes to explain, it has a greater impact.

**Interviewer: Are there any health campaigns in your area?
R5:** No, there are none.

**Interviewer: For those with smartphones and access to WhatsApp, if messages were sent there, do you think it would be helpful?**
Yes, it would be helpful.

**Interviewer: What is more effective—voice messages or videos?
R8:** I don’t use a SIM card, but anything on WhatsApp would be helpful. There are already family groups for sharing messages.

**Interviewer: In your household, when it comes to health-related decisions, whose advice is followed more—men or women?
R1:** Men’s advice is mostly followed. Whether it’s about children’s health or taking them to the hospital, we rely on the men to make decisions.

**Interviewer: What about other households—whose opinions are prioritized there?**

**R2:** In my house, I make most of the decisions. When my child is sick, I go wherever I feel necessary.
**R4:** In our house, my mother makes the decisions. My husband left, and my father passed away, so I follow what my mother says.

**R2:** We usually discuss things with each other, and we follow the advice that seems most sensible.

**Interviewer: Do you think community leaders, like mosque clerics or polio workers, play an important role in delivering health messages to the people?
R8:** No, they don’t say much. Polio workers just come, give the drops, and leave. Similarly, the Aga Khan staff comes, gives the vaccines, and leaves. Once, when my child had a fever, I asked them for syrup, and they said they’d provide it but never did.

**Interviewer: Do you think it would help if those who provide medicines or vaccines also shared messages with you?
R8:** Yes, it would definitely help. If they share something, it would be for our benefit, not to harm us.

**Interviewer: What about community leaders or elders in your area—what role do they play in delivering health information and messages?
R7:** In our area, women come to visit my mother, and they also bring medicines for family planning. But when we discuss things, the usual advice is just to go see a doctor.

**Interviewer: Can you tell us about the challenges you face in accessing good doctors?
R6:** The main issue is affordability. Those who can afford it go, and those who can’t don’t. With the current inflation, many times we can’t go at all.

**Interviewer: Do you face issues in getting to the clinic?
R5:** No, we just walk there since it’s nearby. The main issue is money. If we have the money, we go.

**Interviewer: Are the clinics private or government-run?
R3:** There are both. We have private clinics and also government ones, like Jinnah Hospital. We go there too.

**Interviewer: If messages were to be delivered by Lady Health Workers (LHWs), Aga Khan staff, or anyone else, what topics should they focus on?
R3:** Children’s hygiene, what to feed children, and women’s health issues.
**R4:** There should also be discussions about family planning injections and menstrual health after those injections.

**R1:** Child health and nutrition—what to feed children.

**Interviewer: Have the LHWs who visit your homes ever shown you videos?
R1:** Yes, they’ve shown videos. Last time, they showed one about breastfeeding. It was very helpful. They explained how to hold weak babies close to the chest, and the video was very beneficial.

**Interviewer: Did you share the information from that video with your neighbors?
R1:** Yes, I told my neighbors how to take care of their children.

**Interviewer: Thank you all very much for taking the time to share your insights!**

**FGD 7**

**Interviewer:** What do you do in your free time at home?
**R1:** I lie down after doing some chores. There’s a little work, and once the housework is done, that’s it.
**Interviewer:** Like sewing?
**R1:** No, nothing like sewing.
**R2:** I make embroidery work. After finishing household chores, I do embroidery and attach beads on shirts.
**Interviewer:** Do you sell those or keep them for yourself?
**R2:** No, no, just for myself.
**R3:** I do the same—beadwork. I wake up early, send my husband off to work, and then start working on bead designs. I earn money from it.
**Interviewer:** So, do you sell this work?
**R3:** No, I get it from others and then make it.
**R4:** I offer five daily prayers and read the Quran.
**R5:** I also do housework, take care of (points to the child), and do nothing else because my husband doesn’t let me work.

**Interviewer:** Alright, in your opinion, how important is health in the community? For example, cleanliness, prevention of diseases in children, or keeping the surroundings clean—does it help reduce the spread of common diseases? What do you think about this?
**R4:** Yes, of course, it helps. Why not? Cleaning, sweeping, mopping, getting the kids ready—it’s all part of our daily routine. It’s necessary. Bathing the kids daily helps prevent illnesses, right?
**Interviewer:** Okay, you mentioned the surroundings. When children go out to play, how careful are you about their cleanliness at that time?
**R4:** I make them wear clean shoes. Their grandmother sweeps the street before letting them out, otherwise, I don’t let them go outside.
**R5:** My child is very naughty, but I still take great care of him. I protect him from the cold, and every day I make him wear clean clothes. But along with cleanliness, attention to diet is also necessary. If the diet is good, the child will stay healthy. I pay a lot of attention to his food as well.
**R6:** I do housework, cook homemade food, and clean the house. Cleanliness is beneficial. I wash their faces, change their clothes, and feed them regularly.

**Interviewer:** If there’s general information to be shared in your community, like a camp is being organized, or a clinic has some information about a new vaccine, how does that information reach you? Can you tell us about the common methods used in your community to communicate with one another or to spread a message to the people around you? How do you usually get to know about such things?
“Silence”
**Interviewer:** For example, if you need to pass on some information, how do you do it? We’re asking because if we have something important to share with you, we want to know the best way to reach you. What are the different ways? Some people suggest organizing a camp, others suggest door-to-door visits, or calling people on mobile phones. What do you think is the best way?
“No response”
**Interviewer:** Does anyone come to your house to deliver messages?
**R7:** Yes.
**Interviewer:** Do workers come?
**R7:** A doctor comes—Dr. Fatima.
**Interviewer:** What does she inform you about?
**R7:** About anything related to children.
**Interviewer:** How do you call her? Or does she come on her own?
**R7:** She comes on her own, once or twice a week, to check on illnesses and everything. She lives in our neighborhood.
**R1:** Our houses are very close to each other, so we don’t need to call anyone. We handle things ourselves. Since our houses are nearby, we can just go to each other without needing to call anyone. If a child falls sick, we immediately go to the doctor.
**Interviewer:** Where do you go?
**R1:** Anywhere, wherever a doctor is available.
**Interviewer:** How do you go?
**R1:** I go on a bike with my husband, usually around 8 or 9 PM when he returns from work.
**Interviewer:** Do workers visit your house too?
**R1:** No.
**R3:** Whatever work needs to be done or meals need to be cooked, I quickly finish it. Since it’s winter, we go to bed early with the children because they might catch a cold. Kids usually follow their mother around, so I cook, then sit in the room with them. If I get bored, our family is big—there’s my sister-in-law, father-in-law, and mother-in-law. We sit and talk.
**Interviewer:** Do you ask them about health matters?
**R3:** Yes, my mother-in-law knows, so I ask her.
**R5:** Usually, Aga Khan workers come to us, so it’s not an issue. But if something needs to be shared house-to-house, my mother-in-law and my aunt go and inform people. Since we live together, my mother-in-law tells her daughters, and we also inform our relatives and acquaintances in the neighborhood, so the message spreads quickly.
**Interviewer:** So, you don’t use mobile phones or anything like that?
**R5:** No, no. Mostly, we don’t use mobile phones. We find out by visiting each other’s houses.
**Interviewer:** How frequently do Aga Khan workers come?
**R5:** Recently, they came after a long time. They came that day to inform us about an upcoming camp and that we should attend.
**R2:** My sister lives nearby, so if there’s any news, she informs me.

**Interviewer:** Since lady health workers visit your homes every two to three months, apart from that, there are now many other ways to spread information, like playing a video on TV when people are watching, similar to advertisements. Or we could use mobile phones through YouTube channels or something similar. Or we could organize camps. Do you think house-to-house visits are still the best method, or do you think any of these other methods would work well for you?
**R3:** I think mobile communication would be best. Mobile phones provide the most convenience. If we could communicate through mobile, that would be great.
**Interviewer:** Do you use a mobile phone? What do you use—WhatsApp, messages, or calls?
**R3:** I mostly use Wi-Fi.
**Interviewer:** So, what should we use to contact you? Should we send a message?
**R3:** YouTube works, or just send a message.
**Interviewer:** And do you prefer videos, voice messages, or calls?
**R3:** Calls would be better.
**Interviewer:** Do you answer your phone at any time, or do you have specific hours when you’re available?
**R3:** You can contact me at any time. My phone is on 24 hours a day.

**Interviewer:** Who else uses a mobile phone in your home?
**R1:** I use it too. I use it more often. You can call me anytime—I stay at home, and my mobile is always with me.
**Interviewer:** Is calling a good method, or do you prefer video, WhatsApp, or messages?
**R1:** Calling is better because you get instant information. With messages, you don’t always know when they were sent. Calls are more reliable because you can directly talk, while with messages, you might not even know when they were sent.

**Interviewer:** Do you have a mobile phone? Can we send messages to you through it?
**R5:** Yes, you can. But I prefer WhatsApp because I use it more frequently.
**Interviewer:** Should we send a video or a voice message?
**R5:** A video would be better. It will give clear information, but it would also be better if there’s an address provided.

**Interviewer:** Who has a mobile phone in your house?
**R4:** My husband.
**Interviewer:** Is it okay if we send a message to his phone?
**R4:** Yes, the Aga Khan people contact him on his phone as well.
**Interviewer:** Does your husband inform you when there’s a call?
**R4:** Yes, he will let me know if there’s a call from the hospital.
**Interviewer:** If we send a video, will you be able to watch it on his phone?
**R4:** Yes, yes, I can watch it.

**Interviewer:** Who has a TV at home? Who watches it?
(Two people said yes)

**Interviewer:** Okay, tell me, what do you like to watch on TV?
**R6:** I like watching movies and dramas.
**Interviewer:** On which channel?
**R6:** I don’t watch on cable; we have a dish.
**Interviewer:** Apart from dramas, anything else?
**R6:** I listen to songs, watch films—that’s about it.
**Interviewer:** Do you think TV is a good way to convey health messages, like through ads or videos?
**R6:** No.
**Interviewer:** What time do you usually watch TV?
**R6:** At night, around 10 o’clock.
**Interviewer:** If we run an ad at that time, would you understand it?
**R6:** No, I wouldn’t understand it. How would I get it on TV? We don’t have cable.
**Interviewer:** So, what method do you think would be better?
**R6:** I don’t use a mobile either. It would be better if someone came to our house and explained it.
**R1:** We have a TV at home too, but I don’t watch TV much—I use my mobile more. I watch TikTok, movies, or dramas on my phone. Sometimes, I also check WhatsApp if there’s something important.
**Interviewer:** So, do you watch TV at night or not at all?
**R1:** Sometimes I sit down for 20 minutes at night, but not for long.
**Interviewer:** Do you think TV is a good way to deliver a message?
**R1:** You get the message on the phone, but not on TV. On mobile, if something comes on WhatsApp, you know right away.

**Interviewer:** If you need to receive a health message or go to a doctor, what kind of difficulties do you face? For example, if you’re using a phone and it’s out of charge or there’s no electricity. Or if you don’t have a phone at home and a child is sick, how do you send a message to someone or get help?
**R1:** We wait until evening. We wait for my husband to come home from work, and then we go to the doctor. He’s the one who takes us—we don’t go alone.
**Interviewer:** Is there no one else you can inform or call for help?
**R1:** No. My in-laws are at home. We ask my father-in-law, and he takes us, or sometimes my brother-in-law.
**R7:** I go to the doctor myself.
**Interviewer:** Do you face any difficulties in getting there?
**R7:** Sometimes I walk, and sometimes I take a vehicle.
**Interviewer:** Do you think it would be easier if someone gave you advice over the phone?
**R7:** Her grandmother’s house is far away.

**Interviewer:** Apart from this, when lady health workers come to your homes, do you all understand Urdu?
(Collectively responded yes)

**Interviewer:** How often does the electricity come and go in your homes?
**R6:** There are a lot of power outages.
**Interviewer:** How many times a day, and for how long?
**R5:** It goes out four times a day for one and a half hours each time. It starts at 10:30 in the morning and ends at 10:30 at night, and after that, it doesn’t go out again.
**Interviewer:** Do you face any problems because of power outages?
**R5:** Yes. Firstly, the Wi-Fi stops working, and secondly, sometimes we run out of water, and we can’t turn on the water pump. Other tasks like ironing also get disrupted, so it’s very troublesome.

**Interviewer:** At what times do you use mobile phones?
**R5:** I use it during my free time, mostly in the morning. After breakfast, once all the chores are done, I use it.

**Interviewer:** Apart from this, are there any cultural or social norms in your community? Like in some places, men make the main decisions—for example, when to go to the doctor. You mentioned earlier that you wait until evening for your husband. What kind of issues do you face because of such norms?
**R6:** My husband comes back from work, and then I go with him on the bike. I don’t know where the doctor’s clinic is. I came from Punjab, so I’m not familiar with the area.
**Interviewer:** Who makes decisions in your household?
**R6:** My husband makes the decisions. He informs the doctor about everything.
**R1:** Yes, he comes back from work—he works on daily wages. Once he earns his wages, we can go to the doctor.
**Interviewer:** Regarding household chores, do you make the decisions, or does your husband?
**R1:** My husband makes the decisions.
**R2:** In our house, my father-in-law makes the decisions.
**R3:** My husband makes the decisions.
**Interviewer:** Do you also participate in decision-making with him, or is it only him?
**R3:** Only he makes the decisions, like when to go to the doctor.
**Interviewer:** My point is, if you disagree with your husband’s decision, do you still follow his decision, or do you act according to your own choice?
**R6:** I follow my husband’s decisions. First, I have to listen to my father-in-law, then my husband.
**R5:** Both of us make decisions together. If the child’s condition is more serious, I can go with my mother-in-law or sister-in-law—my husband has allowed that. Otherwise, if he comes home, we decide together where to go. Unlike others who say their husbands solely make decisions, that’s not the case for us. We make decisions together after consulting each other.

**Interviewer:** What kind of work do your husbands do?
**R6:** My husband works with utensils.
**R1:** Utensils.
**R4:** Company.
**R5:** Company.
**R2:** My husband drives a truck and transports goods from the port.
**R3:** Company.
**R7:** Company.

**Interviewer:** In your opinion, do community leaders, such as lady health workers, mosque imams, or school teachers, play any role in your community regarding health? Do people listen to them? Is there anyone influential in the community?
**Collectively:** “There isn’t anyone like that.”

**Interviewer:** Does the mosque ever make announcements about camps?
**R5:** There aren’t any announcements in the mosque, but, as I mentioned, we find out from other women. When Aga Khan staff come, they inform us that a camp is being set up.
**Interviewer:** When do you usually meet with other women?
**R5:** Just whenever there’s free time, if someone hears about something, they immediately come over to inform others. Or, if my mother-in-law hears about it, she goes to tell her sister. In the evening, when women step out of their homes, they talk to each other.
**Interviewer:** Do you think announcements made in the mosque would be a good way to inform people, and would the message reach you?
**Collectively:** Yes.

**Interviewer:** If we set up camps, would you all come?
**R6:** If our husbands allow it, we’ll come; otherwise, we won’t.
**Interviewer:** Usually, do you get permission to attend camps? For example, today you came here.
**R6:** My husband went to work today, so I asked my mother-in-law before coming.
**Interviewer:** Besides getting permission, do you think these camps are helpful? Have you ever attended such camps in your community?
**R6:** No, we’ve never seen any camps.
**Collectively:** No.
**Interviewer:** If there’s a camp, will you come? And how likely is it that others around you will attend? How many will get permission?
**R6:** If we decide to come here, they’ll likely come too. It depends on whether we get permission at home—if we do, we’ll come.
**Interviewer:** Do you think this is a good way to spread the message?
**Participants:** Send a message.
**Interviewer:** To whom?
**R3:** To my husband.
**Interviewer:** Does he have a smartphone?
**R3:** Yes.
**Interviewer:** But he’s not home from morning till evening, so he’ll tell you at night?
**R3:** He informs my mother by calling her, and her house is nearby.
**Interviewer:** Besides that, is there any other way?
**R3:** Go door to door and inform people.

**Interviewer:** Which of these methods is the best way?
**R6:** Coming to the house and telling us.
**Interviewer:** What if a video is shown at the house?
**R6:** Whatever the method, just come to the house, and we’ll understand.
**R1:** Like how they came earlier to explain the issue, we understood right away.
**R3:** Yes, it’s better to come to the house. Bring it directly to us.
**R2:** Whenever they come to call us, I come right away.
**Interviewer:** So, you think they need to come to your house, or else you won’t know?
**R2:** No.
**Interviewer:** Any other way?
**R2:** Just call directly and tell us the issue; the conversation is over. A simple call is enough to explain.
**Interviewer:** Do you have your own phone?
**R2:** No, it’s my husband’s phone.
**Interviewer:** What do you think is the best method?
**R5:** I think going door to door and explaining is the best method.
**Interviewer:** Why is that? Does it make things easier to understand?
**R5:** Yes, definitely, we understand it better, and trust is built, which is the most important. Calls can sometimes be fake, so it’s better for them to come and talk in person.
**Interviewer:** What about you?
**R8:** Going door to door and telling us.
**R7:** Send a message.
**Interviewer:** How should the message be sent?
**R7:** My daughter has a phone, so it can be sent through hers.
**Interviewer:** Does your daughter inform you?
**R7:** Yes, she lives with us.
**Interviewer:** Can we call her at any time?
**R7:** Yes.

**Interviewer:** What information would you like to know? Is it related to children’s health or women’s health? What kind of information do you want us to provide?
**R6:** Just get medicine for my child. She has a cold, cough, and ear pain. I’ve tried many medicines, but she’s still not feeling better.
**Interviewer:** Anything else?
**(Inaudible)**
**Interviewer:** Do polio workers visit your home? How often do they come?
**R6:** Yes. They come every month, every two weeks.
**Interviewer:** Besides the polio workers, does anyone else come?
**Collectively:** No.
**Interviewer:** Is there anything they don’t talk about but you would like to be informed about?
**Nothing.**

**Interviewer:** Besides doctors, do you all consult your in-laws before going to the doctor?
**Collectively:** We go directly to the doctor.
**R8:** I go with my mother.
**Interviewer:** Does your mother give advice first, or do you go directly to the doctor?
**R8:** We go directly to the doctor.

**Interviewer:** Thank you.
