# Supplementary material for: Bridging the health literacy gap: Women and community stakeholders’ perspectives on maternal and child health communication in peri-urban Karachi, Pakistan
Source: PLOS Digit Health. 2026 Aug 27;5(8):e0001632. doi: 10.1371/journal.pdig.0001632 (PMC13521370; doi:10.1371/journal.pdig.0001632)
Supplement: Supplementary file 4 [file pdig.0001632.s004.docx]

**KII 1**

**Interviewer:** What is your age?
**Respondent:** I am sixty-six years old.

**Interviewer:** Okay. What is the highest level of education you have completed?
**Respondent:** I have done my MS (Master's degree).

**Interviewer:** What is your current role in employment?
**Respondent:** I am the president of the “Mihigiri Samaji Sangat”, part of the Sindh Coastal Organization.

**Interviewer:** What is your current role in the community?
**Respondent:** My residence is within this community, in this coastal area, Rehri Goth. My role is, first and foremost, being a fisherman myself. Beyond that, I listen to and understand the issues of the fishing community and act as a bridge between relevant institutions or individuals and the person facing the problem. When decisions need to be made, I ensure they are made with mutual consultation, and I play a role in those decisions.

**Interviewer:** How long have you been involved in community service?
**Respondent:** I’ve been involved since my student life. After I completed my matriculation and went to college, then Karachi University, and graduated, I applied for a job. At that time, my grandfather knew I was on the path to becoming an officer. He stopped me, saying, “You are the first person from the fishermen community to attend university and graduate. If you become an officer and take up the same work as others, who will do social work for this community? The purpose of educating you was for you to stay here and work for the development of this community, focusing on education and other essential matters.” So, after returning from university, I started fishing and then formally began working for my community.

**Interviewer:** Approximately how long has it been since you started this work?
**Respondent:** I was born here, but I’ve been actively involved in social work for about forty years now.

**Interviewer:** Let’s move on to some key questions. What are your thoughts on the health of the people in this community?
**Respondent:** Regarding health, my perspective is that in our country, after every twelve kilometers, the language, dialect, and thinking of a community changes. Even their culture has subtle differences. Correspondingly, every individual has some knowledge or awareness about their health, but modern research and advancements don’t always reach them. People largely follow traditional practices handed down from their ancestors. Then, when health professionals or others connected to health reach out to the community, people often adopt their suggestions, not necessarily out of awareness but more as a matter of belief.

**Interviewer:** If we specifically talk about Rehri Goth, do people here follow the same cultural practices?
**Respondent:** Most people follow the same practices. However, changes are occurring over time. These cultural practices and traditions are tied to the community’s economy and livelihood. Wherever people find work or the nature of their work changes, they adapt their culture and traditions accordingly.

**Interviewer:** If we talk about this area, specifically regarding the health of mothers and children, could you provide an example of something related to health that regularly occurs here and is influenced by the cultural practices you mentioned?
**Respondent:** Look, we’ve done a lot of work on maternal and child health here. About thirty years ago, when I was working with Dr. Ghaffar Billoo’s team, we started social work here in Rehri Goth, focusing on maternal and child health. At that time, they were already working on these issues but faced challenges in connecting with the community because of cultural barriers.

**Interviewer:** Such as?
**Respondent:** For example, fishermen here traditionally do not wear slippers or shoes. Economically, they need to be ready to go into the water at any moment, so footwear doesn’t suit their work. For fishing, they are accustomed to staying barefoot. They also viewed anyone who wore slippers or shoes, or dressed in pants and shirts, as outsiders or people who weren’t part of their trade. Since their work revolves around tides—low tide, high tide—they don’t follow a set schedule. They go fishing whenever the tides allow, whether during the day or night. Mentally, they are always prepared to go to the sea or board a boat at a moment’s notice.

A captain, or *nakuda*, can call his crew, the *khilasis* (helpers), to the boat at any time. His orders are final. Even if the fisherman’s child or wife is sick, he must leave them behind and follow the *nakuda’s* command. Only after reaching the boat can he share his problem with the *nakuda*, who may then grant him permission to return.

**Interviewer:** Who is the *nakuda*?
**Respondent:** The *nakuda* is the captain of the boat.

**Interviewer:** So, what you’re saying is that when you were working earlier, the community had problems because, as you mentioned, their practices were such that they didn’t wear shoes or pants and shirts. So, do people who come dressed like that, or wear shoes, the community keeps their distance from them?
**Respondent:** Yes. Back then, they believed that anyone who wore pants and shirts or dressed differently was more educated and not part of their world.

**Interviewer:** And what about now? Is this still the case?
**Respondent:** There has been significant change. The community’s perspective has evolved. Now, people wearing pants and shirts are not seen as outsiders based solely on their attire. The newer generation, especially women and children working in industrial sectors outside of fishing, wear clothing suited to their work environment, like pants and shirts, for ease while operating machinery. Now the community accepts that this type of clothing has nothing to do with education or awareness. However, the older generation, people my age or older, still holds on to the old mindset that equates attire with status or education.

**Interviewer:** Let’s talk about the present. Regarding health, what kind of issues do you think are prevalent now? What challenges exist?
**Respondent:** Look, in terms of health, particularly regarding mothers and children, 30 to 40 years ago, mothers tried to fulfill their children’s nutritional needs themselves. For instance, they believed breastfeeding was essential for their child. Then television arrived, and with it, advertisements. When mothers saw this new world on TV, they shifted from breastfeeding to formula milk.

When this shift happened, their children began falling sick more frequently, leading them to visit doctors. These mothers couldn’t understand why their older children born through normal deliveries and exclusively breastfed were healthier compared to their younger ones. After television arrived, especially after 1974, they started seeing ads promoting foods like packaged milk, cereals, and other processed items, which they thought might be better for their children. However, six months to a year later, health problems started appearing in their children.

**Interviewer:** So does every household here have a TV?
**Respondent:** Almost every household used to have a TV. People watch TV less now, but I’m talking about the time from the 1970s to the 1990s.

**Interviewer:** But currently, does everyone here own a TV?
**Respondent:** Yes, TVs are present.

**Interviewer:** Do people watch them? And what channels are most popular?
**Respondent:** Mostly, they watch Sindhi channels and Indian dramas. Pakistani Sindhi channels are also popular.

**Interviewer:** Any specific names?
**Respondent:** KTN, Sindh TV, these are the most-watched channels. Women over 30, especially working women, prefer drama channels. Indian dramas on Indian channels and Pakistani dramas, especially on *Hum TV*, are particularly popular.

**Interviewer:** Is there a specific time when people, especially women, watch TV more frequently?
**Respondent:** Women can be divided into three categories. One is housewives who do housework. They usually have time between 10 AM to 12 PM and then from 2 PM to 6 PM if they wish to watch TV. However, the majority of women watch TV at night.

**Interviewer:** Is there any issue with electricity here?
**Respondent:** Load-shedding is an issue. During power outages, if women’s TV watching gets disrupted, they often turn to their mobile phones to stream TV channels.

**Interviewer:** So we’ve talked about women, but what about men? What is their trend?

**Respondent:** Men watch less TV. You see, for about 17 to 18 days in a month, they don’t go near a TV because they’re out fishing. When they’re at home, they spend the remaining 12 to 14 days with their families. But for those 18 to 20 days, they’re out fishing. In the fishing areas, there’s no TV, so they use mobile phones instead.

**Interviewer:** And what do they watch on mobile phones?

**Respondent:** Mostly, people watch TikTok. They watch TikTok and Indian movies. And usually, the captain of the boat, the one who has some responsibility, stays connected with news channels. The captain feels like the head of the family in a way. So, those who feel a sense of responsibility towards others try to stay informed, whether through news channels or updates about the weather.

**Interviewer:** So if this captain is out fishing, how does he access the news? What medium does he use to stay updated?

**Respondent:** Through mobile phones.

**Interviewer:** Is there any specific app they use?

**Respondent:** These days, most news channels have their presence on Facebook, so they access it there.

**Interviewer:** So, for men in the fishing community, the captain is someone they listen to, and he conveys the news to them. Would they follow what he says? If we wanted to send a message about maternal and child health to the men, who would be the key person or channel to identify, in your opinion?

**Respondent:** Let me share an experience with you. Back in the ‘90s, when we wanted to work on education in our community, we started “Miahigiri Samaji Sangsat”. We identified 11 people who could read and write—people who used to read newspapers or send letters to radio stations during music request programs. Through a survey, we found these people who were connected to the communication mediums available at the time.

We discovered 11 such individuals and worked with them to improve education and health awareness in the community. When we talked to them about education, they told us, "Sir, most people are illiterate, so going door-to-door will be difficult."

I suggested an idea that worked well for us. I told them to start by chalking messages on walls. So, we began. We targeted all the mosques in the area and wrote messages on the walls outside. For instance, we wrote in Sindhi:

- *“Educate your daughters, and you will earn respect.”*
- *“Education is a precious ornament for your daughters.”*

We tried to align with the community's psyche. When we wrote "education," we made sure to add “(Quran Shareef)” in brackets. We knew that those who read it would connect with the religious aspect. While our intent behind "education" was general, including the Quran in brackets helped address those who might resist education for cultural or religious reasons.

At that time, there were seven mosques in Rehri Goth. Apart from that, we targeted alleyways and main roads where people frequently passed. On the main roads, we wrote messages in Sindhi for the local community and in Urdu for outsiders.

For outsiders, we wrote messages like:

- *“Welcome to Rehri Goth. We hope that when you talk to your hosts, you will also discuss the importance of educating their daughters.”*

**Interviewer:** So, in your opinion, apart from chalking, do you have any other suggestions?

**Respondent:** No, I've prolonged my story . When we did the chalking, it led to something interesting. The religious people, especially the imams of the mosques, started addressing us in their Friday sermons. So, we stationed one of our workers outside every mosque to stand there after the sermon, in case anyone had questions.

We had written the names of our team members under the chalked messages as well. During the sermon, the imams spoke for an entire hour and a half about education. They said things like, *"Look at these young men who want to corrupt our daughters. They're promoting education, which is not good. They want girls to go outside, study, and ruin our honor and dignity."* They approached it from that angle.

But our goal was achieved because when the imams talked about it, our message reached every individual in the community. Afterward, people came to us and asked, *"What wrong have you done that the imam is arguing about it in his sermon?"*

We then explained to them, *"All we wrote on the wall was, ‘Educate your daughters and teach them the Quran.’"* This made things easier for us. Within a single day, we had connected with the entire community. Everyone said, *"Oh, this is a good thing you’ve written."*

From within the community, we found people who told us, *"My daughter is already learning the Quran,"* or *"My daughter has studied up to fifth grade."* So, we found a team from within the community, those who were already aligned with our cause. These individuals came forward during our survey, and we realized there were educated women in our village as well.

This expanded our own team and made our work easier. Back then, the purpose of our work was to encourage religious people to value non-religious education alongside religious teachings. Our message naturally reached the people through the imams’ sermons.

**Interviewer:** What about now?

**Respondent:** Even now, in this community, there are three figures that people trust and listen to. These are the mosque imam, the school teacher, and the family doctor. People trust all three of them.

**Interviewer:** How many mosques are there in this area approximately?

**Respondent:** Now, there are 11 mosques.

**Interviewer:** And school teachers?

**Respondent:** There are about 15 schools within Rehri Goth.

**Interviewer:** Are they spread across all neighborhoods?

**Respondent:** Yes, almost every neighborhood has one.

**Interviewer:** So, is there a teacher in every neighborhood whose advice is respected?

**Respondent:** Well, another thing is that there are decision-making processes within the community. By that, I mean there are small councils. In every fishing community, there are groups of five to six people who address internal disputes and conflicts weekly. It’s like a small court, and decisions are made within a day or two. For example, disputes between husbands and wives, siblings, property conflicts, business issues, or even disagreements about fishing zones, like "this is my area." In every neighborhood, there are two or three people who listen to complaints and resolve them themselves. If they can't resolve it, it goes to a larger council, such as the "Mahigiri Samaji Sangat," which is an organization dealing with such matters. Beyond that, there’s a jury within our community that handles issues. They convene and listen to these matters.

**Interviewer:** Does this council have a specific name?

**Respondent:** It’s called the *Raad* (unsure of pronunciation). The *Raad* involves everyone in the community—men and women. Among them, the respected individuals listen to the issues, consider witnesses and the situation, and then make a decision.

**Interviewer:** Do they only address issues, or do they also disseminate messages related to education?

**Respondent:** It’s more about how you convince them. The focus is on satisfying them. Once they’re convinced, they relay the message to the relevant sector or people.

**Interviewer:** Another thing—there are 11 mosques in the community, as you mentioned. Do the imams have any influence? How much do they prioritize matters related to maternal and child health?

**Respondent**: Look, the first thing is, it’s possible that you might disagree with me on this matter, but in our country, the people who have received religious education are still being taught the same things that were taught 14 or 15 years ago. Their curriculum remains the same. The current needs are not included in their curriculum. So, their priority is that, first of all, a woman is meant for the home. If she steps outside the home, in their mindset, it’s as if she is not a good woman. If she goes out without a burqa, according to their mindset, she is not considered good. Even if you try to convince them, there is still a difference. However, in this community, there is a difference. In this fisherman community, from the beginning until now, the decision-making power has always been with the woman. Especially in the fisherman community, the decision-making power in the household lies with the woman.

**Interviewer:** Why is that the case?

**Respondent:** The reason is simple: as a fisherman, I have to go fishing. My wife stays back to manage the home. Whatever I earn, I don’t keep it with me—I entrust it to my wife. She’s responsible for the home, the children, and even my elderly parents. So, decisions about the children and household ultimately lie with her.

**Interviewer:** Yet, we often encounter women here who hesitate to act on advice that goes against cultural norms, such as opting for hospital deliveries. They often say, "I’ll consult my husband first," or "I need his permission." This is especially challenging because fishermen are often away at sea. What’s your perspective on this?

**Respondent**: Look, since the advancement of medical science, before that deliveries were taking place, and they used to be done by dais (traditional birth attendants). The maternal and child mortality ratio during those deliveries was very low. One reason for this was that both the mother and child had access to a plentiful amount of nutrition. For example, the woman, being from the fisherman community, had fish twice a day, every 24 hours, which was necessary for her. When the mother ate fish, it was obvious that the protein in the fish would be absorbed into her body and then transferred to the child, making both healthy.

Secondly, women here, from the beginning to the end, would go to the boat with their brother or husband to fish. They would work, and a person who works remains physically fit and healthy. Even if a woman did not work directly, she would still accompany them on the boat, staying on the islands for a month to six months. While there, she would continue to work—when the boat would return with the fish, it was the women who cleaned, dried, and processed the fish. They would also go into the jungle to collect firewood and prepare food. This physical activity provided them with a lot of exercise.

**Interviewer:** So, does this not happen anymore?

**Respondent:** No, this doesn’t happen anymore. Secondly, when medical science advanced, earlier, if a woman or a man fell ill, they were treated through traditional remedies, such as family-taught cures or concoctions like herbal teas for coughs and fevers. But as medical science progressed, unfortunately, those who had proper health education, whom we call doctors, couldn’t reach this community. Due to the lack of transport, doctors found it too far to visit.

So, people from nearby informal settlements who would go into the city to see proper doctors ended up interacting with dispensers working under those doctors. These dispensers realized that people from these areas were coming to them. The dispensers then set up their own clinics in the community. As a result, the people, instead of going to proper doctors or hospitals (to avoid transportation or time wastage), thought these dispensers were actual doctors and started relying on them.

For the past 40 years, these dispensers have been dealing with the community. However, over the past 20 years, a new issue arose: some dispensers didn’t even have basic qualifications. For example, one dispenser who claims to be a doctor used to work as a cowherd, taking care of buffaloes. He became a dispenser and set up a clinic. Once he earned enough money, he raised buffaloes and hired a cowherd to look after them. This cowherd would give injections to the buffaloes, sometimes unnecessarily.

Eventually, this cowherd started administering injections to people too. The dispenser-turned-doctor told him, “If you can inject a buffalo, you can inject people for fever.” That’s how the cowherd began giving injections. Now, the cowherd has set up his own clinic here, right next to us, and 50% of the population goes to him. He knows how to make people dependent on injections.

If someone goes to an MBBS doctor, they’ll receive a proper prescription tailored to their condition. But this cowherd-turned-dispenser tells patients, “This is a painkiller. Let me give you two or three injections and put you on a drip.” He uses this mindset to make people believe in his treatment. The drip is given under the false impression that it is highly nutritious. Sometimes, he adds a little multivitamin to change the color of the drip, and the slow drip that runs for an hour convinces people they’re gaining strength from it.

**Interviewer:** I’ll interrupt you briefly since we’re short on time. I’m trying to understand why they trust him so much. Why do they rely on him more than a proper doctor?

**Respondent:** One reason is that he gives them high doses of medicine. If someone is in pain, instead of checking their weight or physical condition and prescribing a suitable tablet or syrup, he immediately administers a Dexa injection. Dexa causes dependency. When the patient receives Dexa and a painkiller, instead of the prescribed dose for example if he needs 100cc dose, he gives them 200cc or even 300cc.

**Interviewer:** So, they get quick relief from his treatment?

**Respondent:** Exactly. They feel relief quickly and think, *“This educated expert doctor is amazing. One injection, and I’m fine!”* He instills this mindset, saying, *“See how skilled I am? You came in with fever and pain, and with just one injection, you’re cured. That’s the difference between me and others.”*

**Interviewer:**
Okay, so you talked about *dais* (traditional birth attendants). Are *dais* still widely consulted here, and do most pregnant women go to them?

**Respondent:**
Yes, absolutely, they still go to *dais*. This is because communication with them is easier, and secondly, *dais* also provide home services—they go to the women’s homes. Once a woman is sure she’s pregnant, it becomes convenient for her to reach out to the *dai*, even late at night, like at 1 or 2 a.m. The *dai* facilitates her and gives basic guidance, such as dietary advice like, “Eat this, use that.” This convenience makes it easier for the women.

If the *dai* belongs to the same neighborhood, financial matters usually don’t play a role, which makes it even more comfortable for them. However, *dais* have also developed their own networks. For example, if a *dai* doesn’t charge for her services, she might charge for preparing herbal remedies or medicines, like, “I’m making this tonic or herbal mixture for you,” or, “I’m bringing medicines based on recommendations from a certain lady doctor. Pay me for that.” This way, she earns her profit.

Another aspect is that, in this fisherman community, there are many private clinics. However, very few have qualified lady doctors; most rely on midwives. These midwives work in collaboration with the *dais*. They have a commission-based system where the *dais* refer patients to these midwives or doctors.

So, the usual pattern is that the woman first goes to the *dai*. After that, the *dai* tries to direct her to the midwife or doctor she’s affiliated with.

**Interviewer:**
How actively does this community participate in health-related programs or activities?

**Respondent:**
Participation depends on their available time. For instance, if you try to engage people during their fishing hours, they’ll say, “We don’t have time; we have to fish.” But if you approach them during their free time, they’ll attend.

Another thing about this community is that they are not used to waiting—they don’t like standing in lines, whether it’s men or women. They find it frustrating.

Also, if you organize a meeting or event, they won’t pay attention to the organizers or facilitators until the main speaker arrives. When the speaker shows up, people start gathering, thinking, “Now the person who will address us is here.” Then, more people join.

When the topic resonates with their mindset and needs, their participation exceeds expectations. However, if they don’t understand the message—no matter how good the speaker is—they’ll leave after just a couple of minutes.

**Interviewer:**
What do you suggest for improving communication and delivering messages effectively?

**Respondent**: Look, my advice is that if you are talking about health, you will need to categorize women into three groups. The first group is those who are in college or school, meaning the teenagers, the young girls. These are the upcoming women, and their category needs to be separated. After that, there are the women who are currently working, particularly in the industrial sector. Their lifestyle and culture have changed a lot. Their mindset has undergone many changes as well. The third group is those women who are housewives, who stay at home. These three groups need to be separated, and then their needs should be addressed accordingly.

**Interviewer:**
For example, you mentioned housewives watch a lot of TV and dramas. What about working women—what are their priorities?

**Respondent:**
The factories where these women work don’t take proper care of labor conditions. These women are overburdened—they are made to work double shifts.

When they return home, they’re so exhausted that they don’t even realize where they’ve left their bag or purse. They just throw it aside, lie down, and rest. They don’t pay attention to proper meals or self-care. Their primary focus is ensuring they wake up on time for work the next morning.

**Interviewer:**
But they must do something for entertainment as well, right?

**Respondent:**
They have one day, Sunday, which is their day off. Secondly, those who reach the supervisor rank have less free time, so they use social media.

**Interviewer:**
What do they use on social media?

**Respondent:**
On social media, it’s mostly TikTok and WhatsApp. These two apps are used the most.

**Interviewer:**
And what about school-going girls?

**Respondent:**
For school-going girls, they have two main activities. One is their proper school or college, and after that, when they come home, they have tuition time. We also try to form groups at different places where, once or twice a week, we conduct counseling sessions and spend time with them.

**Interviewer:**
So do these school and college girls also have mobile phones?

**Respondent:**
Yes, absolutely, all of them do.

**Interviewer:**
And these are the smart, touchscreen ones?

**Respondent:**
Yes.

**Interviewer:**
Is there a particular network that is more commonly used here?

**Respondent:**
There are two main networks: Zong and Jazz.

**Interviewer:**
And is the internet accessed through devices?

**Respondent:**
There are internet devices too, but it’s mostly on mobile phones.

**Interviewer:**
And what about the boys? Do they also use mobile phones?

**Respondent:**
Boys use mobile phones even more than girls. Especially TikTok and WhatsApp—these are very common. Now we have worked on introducing our educated youth to YouTube and Facebook, where they create vlogs related to this community and their skills.

**Interviewer:**
Who is making these vlogs? Are they from your community?

**Respondent:**
Yes, they are from our community.

**Interviewer:**
So, through these vlogs, information can also be shared, and it’s being done by community members themselves.

**Respondent:**
Yes, it’s being done by community members themselves.

**Interviewer:**
In your opinion, as community leaders or influencers, like yourself, what role do you play in all this?

**Respondent:**
Our aim is to work on improving health, education, and women’s empowerment in our community. This is what we focus on. The result of this effort is that the project you are part of today is also a result of our work. Bringing AKU here and utilizing this entire building and structure for this purpose is part of our organization’s project, “Mahigiri Samaji Sangat.” When AKU first came here, the situation was ____ (unclear). For three months, we conducted a survey in this community, and not a single person was willing to meet with them. Eventually, when they were about to leave, they met our GS. He told them, "Don’t leave yet; meet with our president once, and then make your decision." A meeting was scheduled with him the next day, and three days later, a community-wide meeting was held. Dr. ___ was invited, briefed, and on the fifth day, the community joined forces with them.

**Interviewer**: So, if we want to work in the community and deliver a message, do we need to connect with the influencers in the community? And in the fisher community, as you mentioned, with leaders in every small neighborhood, there is a team that solves problems. You also mentioned the mosques, we should connect with them. Is there anything else you would like to add?

**Respondent**: Another important thing is that here, especially, women have their own small businesses. They cook food and sell it at home. They are a means of connection, a model for other women. So, meeting with them, outside of their working hours, is important because during their working time, they are very busy and won’t be able to spare time for you if you go to meet them. So, you should meet them during their extra time, when they have more availability, and you can satisfy them and communicate the message through them. These women meet many other women daily, stay in contact with them, and that is also a way to spread the message.

**Interviewer**: So, apart from those who cook, are there others who engage in cultural work, like sewing clothes and other such activities?

**Respondent**: Yes, there are two or three women in this community who are very popular. They play an important role. One of them is Madam Ruqqayya, who has been a teacher for forty years and a social activist. Another is Madam Rahima, also a teacher and a social activist. She was one of the founding members of “Mahigiri Samaji Sangat.” These women, who were active before the 90s, had some basic literacy skills at home, and they received opportunities to progress. When this organization started, it acted as a bridge for these women, allowing them to step outside their homes. These women played a major role, as the organization gave them the opportunity to work and even go as far as university, something their parents agreed to only because the organization made it possible. These women have played a significant role in this transformation.

**Interviewer**: Do these women use WhatsApp or are there any groups?

**Respondent**: Yes, they have groups.

**Interviewer**: So, does only the educated class use them, or do all women use them?

**Respondent**: There are three or four groups. Our organization, “Mahigiri Civil Society,” has about 100 to 150 members, including men, women, and children. We also have groups like the “Health Committee,” and there are many others. There are about seven or eight groups on WhatsApp.

**Interviewer**: Does your community also have a Facebook page that is seen by people in your community?

**Respondent**: Yes, on Facebook, “Shamiran” is very popular these days. He makes vlogs and stories. He is young person, a chemical engineer and a fisherman, from this community. The youth really looks up to this content, and even the regular fishermen try to imitate him. People like his work a lot, and they try to catch fish like him. There are a few people like this doing such work.

**Interviewer**: Is this also on Facebook?

**Respondent**: Yes, it's on Facebook, Twitter, WhatsApp, YouTube, and Instagram. It's everywhere.

**Interviewer**: Thank you so much. Is there anything else you would like to share, any advice?

**Respondent**: My advice is this: for the workers in the field, there are a few things I've noticed. To work in the community, it is essential for those who are somewhat educated to understand that they should dress well, wear appropriate clothes, and their appearance should match the facilities available. But the key thing is language. Language is deeply connected to your personality and how you introduce yourself. I tell people, for example, if I’m thirsty, I would say, “Amma, give me a glass of water.” But who is this “Amma”? Can anyone explain who “Amma” is?

**Interviewer**: The mother.

**Respondent**: Yes, the mother. But practically, the one who is my father’s wife is the one who gave birth to me. So, before I was born, she was just my father’s wife. After I was born, she became my mother. If I give the same status to another woman and say, “My father’s wife, give me a glass of water,” the words change, right? The choice of words reflects your personality, your introduction. This is often lacking when people work in the field. They have a shortage of understanding of this. Another thing is that the way they dress should align with the community's mindset. If someone doesn't blend in or tries to act superior, they may not be accepted.

**Thank you.**

**KII 2**

**Interviewer**: How old are you?

**Respondent**: I am 54.

**Interviewer**: What is the highest level of education you have completed?

**Respondent**: I completed my intermediate education.

**Interviewer**: What is your current occupation?

**Respondent**: I used to visit the community for motivation, but now I have stopped going because I am ill. (not sure……)

**Interviewer**: What kind of advice did you give?

**Respondent**: I advised them on which school to enroll their children in.

**Interviewer**: Anything else?

**Respondent**: Then they would ask how much it will cost. I would also give them ways to handle it, such as where the uniform can be found when admitting the child.

**Interviewer**: Which school?

**Respondent**: TCF school. They give half the fees. They do take some fees, but they charge less. For uniforms and other things, they charge a small amount.

**Interviewer**: So your role is to help children go to school, reduce the fees, and other such things, mainly for which women come to you. Besides this, do women come to you for any other reason?

**Respondent**: To get clothes sewn.

**Interviewer**: Anything else?

**Respondent**: For health-related issues. For example, if they have a health problem and don’t know where to go, I would visit their homes and tell them whether to go to Jinnah Sindh or Civil Hospital, or if there is any nearby government hospital. I would advise them to go there for treatment or suggest our hospital for delivery. If nothing works, I would give further advice.

**Interviewer**: Alright. How long have you been involved in community service?

**Respondent**: I started when I was about 19 years old.

**Interviewer**: In the same community?

**Respondent**: Yes, in this community. I wasn’t even married at that time.

**Interviewer**: Okay. Now let’s move to some important questions. What do you think about people’s health in the community?

**Respondent**: In this community, most children don’t get vaccinated or are not even aware of it. They often say that if they have a mild illness, they avoid vaccination. They say, “Just leave it, we are fine like this.” Then I have to explain to them. For example, if they feel something like a headache or stomach pain, I tell them it might be a health issue and advise them to go for a check-up. Like, a woman came to me the other day saying, “I have a burning sensation in my feet and some other problems.” I told her that it seems like she has diabetes because burning feet and weight loss are common symptoms. I then advised her to go to the hospital for a test; we have a small hospital here. They usually listen to me.

**Interviewer**: Okay, you mentioned that many women come to you for vaccination-related advice, and you have to explain things to them. Apart from that, what other things do you have to explain?

**Respondent**: How to clean the house.

**Interviewer**: So, how many people do you think maintain cleanliness in their homes, and how much room is there for improvement?

**Respondent**: About fifty percent (50%) of homes are clean, but I have to explain it well to a lot of people. It’s not like I just go to their homes and say, “Your house is dirty.” I have to meet them where they are. For example, when we visit their home and knock on the door, we explain the purpose of our visit. We talk to them briefly, sit with them. I tell them their house is nice, and if they do this and that, it will be even better. Many women become motivated and start cleaning their homes. The key is communication, sitting with women at their level, and not just standing there talking about this center or that center. You have to sit with them, be like them, and then they become a part of your efforts.

**Interviewer**: Okay, you’re right. My next question is: What role do community leaders play in spreading health messages in these informal settlements?

**Respondent**: Please repeat.

**Interviewer**: I mean, in this community, in informal settlements, when we have to spread health messages, as you said, you visit homes. What role do community leaders play? What do they do to ensure the message reaches everyone?

**Respondent**: Well… (not sure). But in my opinion, when the women of the community gather, like we used to do for small vaccination programs, we would give small gifts. This would attract more women from the neighborhood, and then we would tell them that those whose children have completed their vaccinations will receive these gifts. By giving and encouraging in this way, they start to follow through with the tasks.

**Interviewer**: So, where do you organize these programs?

**Respondent**: In the same community. If someone has a big yard, we invite twenty-five women and do it there.

**Interviewer**: So, do you think people follow the leader’s advice?

**Respondent**: It’s difficult to get them to listen, but some are wise and they set an example, and then others follow.

**Interviewer**: Okay, so what percent of people do you think follow the leader’s advice when things are explained this way?

**Respondent**: Right now, most of them follow. Like recently, a CHW (Community Health Worker), a polio worker, was having trouble getting vaccinations done in her area. Then they told me to go. I went because I was a teacher at that school fifteen years ago, and the children I taught had grown up, these were their children. The women there said, “Let our husbands come, and then you can talk to them and ask them.” When they arrived from the boats, they said, “She is our elder, she taught us, how can we not listen to her?” Then, all the children in that house, around six or seven, got vaccinated.

**Interviewer**: So you mean that community workers and leaders are listened to if people know them and trust that they won’t cause any harm?
**Respondent**: Yes, they do listen. And the most important thing is to become like them, only then they listen. You have to talk to them about their issues, their household problems, or ask about their children. When we do this, for example, we ask about their household issues or say, “How are your children? How are you doing?” they listen because they think, “She is like us.”

**Interviewer**: That makes sense. My next question is: What major challenges do social workers like you face when spreading health-related messages in the community?
**Respondent**: It’s difficult. They sometimes yell and abuse me. But later, they do agree. At first, they say, “Why are you here? What are you trying to make us do? Why do you insist on getting our children vaccinated?” Then I explain the benefits of things, that these are not just vaccines but essential for your children’s lives. Eventually, they agree.

**Interviewer**: So, your biggest issue is when they yell or abuse you?
**Respondent**: Yes, and they also influence others to refuse. When they get angry and abuse, they tell others not to listen to us or comply.

**Interviewer**: Apart from that, what other challenges do you face? Do you face difficulties traveling from one house to another?
**Respondent**: Yes, traveling is also difficult. For example, I quit because the area I was assigned was very large. My knees also started hurting. I was attacked last year during a polio campaign.

**Interviewer**: Can you tell me more about that incident?
**Respondent**: Yes. They refused to let their children take the polio drops. Even now, I hide to do my work without going there myself. Our supervisor used to go and do chalking (marking the houses). When I had just started working, this woman came and asked why I was working there. I said that I’d been working in the area for quite some time. She said, “You will not work here. I will beat you.” I told her, “I don’t want to fight; I don’t know how to fight.” Then she said, “I will beat you.” She picked up a brick and hit me on the head. Then she beat me up a lot, so much that I started bleeding. I was bleeding heavily. Later, the authorities conducted an investigation into the incident.

**Interviewer**: Were these common people?
**Respondent**: They are common people, but known for getting into fights with others. **Interviewer**: So the reason she attacked you was that she didn’t want the children to be given the polio drops?
**Respondent**: Yes, the reason was that in our area, there were two sisters who wanted to do this work. I told them to submit their CV, give an interview, and if they are more capable than me, they will hire them and remove me. But they said they didn’t have certificates or education but still wanted to do the job. Then she attacked me.

**Interviewer**: So, do you work for TCF (The Citizens Foundation) ?
**Respondent**: No, I work for UNICEF. Previously, we were with WHO. Since this year, WHO has been handling our project.

**Interviewer**: Alright. You mentioned transportation issues and physical altercations. Besides that, do you think the lack of smartphones creates hurdles? For example, if smartphones were widely available, you could send messages to everyone. Do you feel that smartphones are less common in the community, or are they easily available?
**Respondent**: Smartphones are very important these days. In this community, even young children have smartphones.

**Interviewer**: So, the lack of smartphones isn’t really a problem for delivering messages? Can we use this medium to convey messages?
**Respondent**: Not everyone has one. In those cases, we have to go to them in person.

**Interviewer**: In your opinion, how many people have smartphones?
**Respondent**: Around 50-60%. The rest just have basic phones.

**Interviewer**: I see. So, to some extent, messages can be delivered through smartphones. Besides that, what methods have you found most effective for delivering health messages in informal settlements?
**Respondent**: The most effective method is to go to them personally and deliver the message, while being like them. The biggest benefit is to engage with them in person. It’s not about just mobilizing or making announcements. The person who directly interacts with the community and maintains that connection is the most effective.

**Interviewer**: Besides this, can health campaigns also be effective?
**Respondent**: Yes, those can be helpful too. We can convey a lot through health campaigns and small programs.

**Interviewer**: If campaigns are conducted in different areas of the community, do people attend?
**Respondent**: Yes, absolutely, they do.

**Interviewer**: And if I ask about social media platforms like Facebook, Instagram, Twitter, or YouTube, which ones do you think are more commonly used in these areas?
**Respondent**: Mostly, people use YouTube or TikTok. Very few are on Facebook or other platforms.

**Interviewer**: Do people watch health-related messages or channels on these platforms?
**Respondent**: No, I don’t think so. Maybe only around 20% do. I’m one of them—I keep watching things related to health.

**Interviewer**: Do you use TikTok?
**Respondent**: No.

**Interviewer**: If I upload a video on YouTube, do you think people in these areas will watch it or not?
**Respondent**: They will watch it. For example, my son has become quite well-known in the area for raising awareness about mangroves—planting them, preventing deforestation, etc.

**Interviewer**: Does he have a YouTube channel?
**Respondent**: Yes, he’s active on Facebook, and he’s also on YouTube.

**Interviewer**: So, he uploads videos there?
**Respondent**: Yes.

**Interviewer**: Is there any popular local channel here that the whole community watches? Or what’s the name of your son’s channel?
**Respondent**: Shahmeran.

**Interviewer**: So, if we post something there, people might watch it, depending on his followers?
**Respondent**: Yes, even young kids watch it. TikTok and similar platforms are also popular, and that’s why they use cell phones.

**Interviewer**: What kind of content does your son upload?
**Respondent**: He uploads his own stories, shares information about his friends, or creates stories related to mangroves. He’s a chemical engineer.

**Interviewer**: Oh, so he works as well?
**Respondent**: Yes.

**Interviewer**: I see. Apart from that, are there any channels in your community that help spread health messages or ones that people trust? For example, any TV channels?
**Respondent**: Yes, we can collaborate with cable providers and have them play health-related videos. They do broadcast them.

**Interviewer**: Which local channel do you use?
**Respondent**: It’s a Sindhi channel; most people watch that.

**Interviewer**: Is there a specific time slot?
**Respondent**: No, it’s 24/7 for those with a connection.

**Interviewer**: If we post something on the channel, which time slot do you think would be most effective?
**Respondent**: In the evening, around 7 or 8 PM, or even until 9 PM.

**Interviewer**: So, if we use that channel, people will listen?
**Respondent**: Yes.

**Interviewer**: Do people listen to the radio here?
**Respondent**: Not really, because now it’s mostly about mobile phones. I’m not sure if they even use radios anymore.

**Interviewer**: I see. Do you know the people who run the Sindhi channel?
**Respondent**: Yes.

**Interviewer**: My next question is about participation in health-related programs and activities in your community. If there’s a health campaign or a leader gives a talk, how many people receive the message, and how many actually attend?
**Respondent**: I can’t say for sure, but around 30 to 40 percent might show up.

**Interviewer**: And if we post health-related content on the channel and 100 people watch it, how many do you think will act on it?
**Respondent**: Very few.

**Interviewer**: Is it because you think that unless you visit people in their homes and have face-to-face conversations, they won’t understand?
**Respondent**: Exactly. That’s what I meant earlier. You have to personally go to their homes and deliver the message.

**Interviewer**: You mentioned that you organize health-related programs. Do people participate in them? Do you offer any incentives? Why do they attend?
**Respondent**: They understand a little bit, which is why they come. When they gain information, they share it with others in nearby homes, telling them why they went and what was discussed at the program.

**Interviewer**: How do you spread the word about these programs?
**Respondent**: The same way I mentioned before. For our community, you have to go door-to-door and inform them. That’s the most effective method, in my experience.

**Interviewer**: When you hold health programs, do educated people attend more compared to those who are less informed or not very educated?
**Respondent**: Both types of people attend, even those who are completely uneducated. They also come if they understand the importance of it.

**Interviewer**: And what about those who are somewhat in the middle?
**Respondent**: They also come, but no group comes at 100%.

**Interviewer**: Alright. Does your community have any specific cultural or social norms that affect how health information is shared and understood? For example, do men’s opinions dominate? If you try to educate women, do they say the men’s words will be followed, and you end up needing to educate men instead?
**Respondent**: Yes, this exists. It’s in every neighborhood, not just this one.

**Interviewer**: So, does it mean men’s dominance is more prevalent?
**Respondent**: I don’t think so. Men often leave for 10 to 15 days, and the women manage everything at home. So, I’d say there’s mostly equality. Even in decision-making, women are included.

**Interviewer**: We’ve heard that in families where men go fishing, women handle more responsibilities at home, while in families where men work other jobs, they take on more household management. Is that true?
**Respondent**: Not in this community. Except for two or three neighborhoods, most areas have equality. Like in this neighborhood, men tend to dominate.

**Interviewer**: Got it. If we were to spread messages via mobile phones, how many households have phones? Do women and men have separate phones?
**Respondent**: I believe every household has a mobile phone.

**Interviewer**: And in how many households do both men and women have separate phones?
**Respondent**: Around 60% of households have separate phones for both.

**Interviewer**: So, if we send a message through mobile phones, there shouldn’t be an issue since most households have separate devices?
**Respondent**: The condition is that they actually check it.

**Interviewer**: Do people have internet access?
**Respondent**: Yes, absolutely. The internet works via cable, and I think people pay around 2000 rupees for it. Most have it installed.

**Interviewer**: Alright. What about WhatsApp? Do people use WhatsApp?
**Respondent**: Mostly, they use WhatsApp. After that comes Facebook, and then other platforms like YouTube or TikTok.

**Interviewer**: If we send a message on WhatsApp, do you think people will pay attention to it?
**Respondent**: The uneducated ones might not, but I’ve seen some girls who are completely uneducated but still know how to use mobile phones.

**Interviewer**: Okay, so in your community, do people prefer messages sent on the phone or on WhatsApp?

**Respondent**: I personally prefer WhatsApp. I even check my husband’s WhatsApp messages.

**Interviewer**: And in your community, do people use WhatsApp more, or do they prefer regular texting through SIM?

**Respondent**: They mostly use regular texting through SIM.

**Interviewer**: Which networks are most commonly used, like Jazz, Zong, etc.?

**Respondent**: Jazz is used the most here, followed by Zong. People try to match their network with their relatives for easier communication.

**Interviewer**: Alright. So, if we send messages on numbers connected to Jazz or other networks, will everyone in the community see them?

**Respondent**: No, not the entire community will see them.

**Interviewer**: In your opinion, what additional resources are needed to improve health information access in your community? What do you think can make message dissemination better?

**Respondent**: Resources like laptops or tablets. Then we can invite the community, show them health-related movies, and even the illiterate ones can watch. Pamphlets could also help.

**Interviewer**: But what challenges do you face with these things?

**Respondent**: Keeping these resources with us and organizing small activities—how many women can we gather? Plus, we also need these things readily available.

**Interviewer**: So, you mean the cost of acquiring these things and managing them is a challenge—like tablets or laptops?

**Respondent**: Yes, their cost and the expenses. Most women say they can't afford it because expenses are already high with food, groceries, and hospital visits.

**Interviewer**: You mentioned tablets earlier. Do you have tablets available at the places where you work?

**Respondent**: No.

**Interviewer**: If you did, would you use them for health campaigns?

**Respondent**: Yes.

**Interviewer**: Is there anything else you'd like to share about health information that we haven’t asked yet?

**Respondent**: Role plays could be helpful. We could gather women and show them health-related issues through small dramas.

**Interviewer**: Should we perform first and then let them participate?

**Respondent**: Yes, exactly.

**Interviewer**: Is there anything else you'd like to add?

**Respondent**: Hmm… What else? (Unclear response)

**Interviewer**: Regarding health information for mothers and children, in your opinion, which topics should be prioritized?

**Respondent**: Regarding health, we’ve taught them to keep their homes clean, get their children vaccinated, and visit the doctor even for minor illnesses. If they can’t afford a private doctor, they should go to government hospitals where there are no fees. These are the messages we can deliver. The most important is to drink clean water—boil it if you can, and if not, use alum (potash alum) in clay pots to purify it. It kills germs. We’ve implemented this practice.

**Interviewer**: That’s great. Thank you so much!

**KII 3**

**Interviewer:** What is your age?
**Respondent:** 25.

**Interviewer:** What is the highest level of education you have completed?
**Respondent:** I have a Bachelor’s degree in Arts and have done some short courses.

**Interviewer:** What is your current job designation?
**Respondent:** I work as a vaccinator.

**Interviewer:** Where?
**Respondent:** At DMC Cattle Colony.

**Interviewer:** For how long?
**Respondent:** It's been almost 3 years.

**Interviewer:** What is your main role there?
**Respondent:** My main role is vaccination. I vaccinate people.

**Interviewer:** Do you also provide education about the importance of vaccination? Does that form part of your role?
**Respondent:** My role is as a vaccinator. I vaccinate and inform people about the effects related to vaccination.

**Interviewer:** How long have you been involved in community service?
**Respondent:** I’ve been in service for 6 years. Before this, I was in UC-5 Nazimabad.

**Interviewer:** Were you also a vaccinator there?
**Respondent:** Yes, I was.

**Interviewer:** In your opinion, what do people in the community think about health?
**Respondent:** Regarding health, people should be given services at their doorstep, like what your Vital team is already doing. However, there are still some areas that are being missed. In such areas where the Vital project wasn’t active, people often demanded services. For example, when I went to vaccinate in remote areas, their demands usually revolved around having healthcare facilities, for addressing cases like measles, scabies, etc. These problems are more common in areas with a lot of trash. They usually ask for medicines to address these issues.

**Interviewer:** So, you think we need to visit homes and provide information for them to listen to us?
**Respondent:** Yes, exactly.

**Interviewer:** How do you think this community perceives health? How important do they think it is?
**Respondent:** When it comes to health, the majority of the community here is not well-educated. They don’t use social media much, so sending messages through it won’t be effective. Perhaps 50% of the community might use it, but the other 50% don’t. So, the best approach is to visit door-to-door and facilitate them directly. We need to raise health awareness so they understand its importance.

**Interviewer:** How much health awareness already exists in this community?
**Respondent:** It’s very low, not significant at all. This community largely comprises laborers who have sidelined their health concerns because they are financially struggling. They don’t prioritize health. When we go to them and explain the importance of vaccinations or children’s illnesses, they demand facilities to address these. Otherwise, they don’t really focus on it much.

**Interviewer:** Can you explain the role of community leaders in spreading health messages in these settlements?
**Respondent:** It can be done through door-to-door visits.

**Interviewer:** Besides that?
**Respondent:** Announcements can be made, or a session can be conducted in the area. Setting up camps and similar activities could also help.

**Interviewer:** Can community leaders take on this role in these areas?
**Respondent:** What do you mean?

**Interviewer:** For example, since you are already involved in the community, could you and other such leaders work together to deliver these messages?
**Respondent:** People could be gathered at camps through announcements. That might work.

**Interviewer:** Where would you like announcements to be made?
**Respondent:** Announcements could be street by street or door to door.

**Interviewer:** What key challenges do you face when spreading health messages in the community?
**Respondent:** The main challenge is that, for example, when children develop a fever after vaccination, the parents are already upset. When we go for vaccination, it’s not like everyone refuses; they don’t outright refuse, but they ask for medicines like Panadol to manage the fever. Many people don’t even understand the importance of vaccination. We explain that it’s beneficial and protects against diseases, and only then do they agree. Otherwise, 30% to 40% don’t get their children vaccinated.

**Interviewer:** What specific issues cause people to refuse?
**Respondent:** Refusal is a big issue. There’s no systematic method in place to convince them about the importance of vaccination. However, we try to convince them ourselves when we visit, and we’re often successful. Convincing them and addressing their demand for medicines are the major challenges.

**Interviewer:** Does transportation also pose a problem?
**Respondent:** Not in all areas, but in some areas, it is an issue.

**Interviewer:** How many people in the community have smartphones?
**Respondent:** Around 50% to 70% of people have smartphones.

**Interviewer:** With internet access?
**Respondent:** That’s hard to say, but it’s likely that 50% to 70% of those who have smartphones use the internet.

**Interviewer:** What methods have you found effective in spreading health messages in informal settlements?
**Respondent:** Door-to-door visits. That’s the most effective.

**Interviewer:** Apart from that?
**Respondent:** Announcements, like those made in mosques. That also plays an important role, but it’s not as effective as door-to-door visits.

**Interviewer:** You mentioned announcements in mosques. At what times and how frequently should they be made? How many people attend?
**Respondent:** Many people come to the mosque. For example, if you’re setting up a camp in the morning, you can coordinate with the mosque authorities and have them make an announcement during that time. They will announce it for you.

**Interviewer:** Besides these, do you have any other methods in mind?
**Respondent:** Messages can be sent, 50-70% of people are already using phones. However, in my opinion, the best method is still door-to-door visits.

**Interviewer:** Which applications are most commonly used on their phones? YouTube, Twitter, TikTok, etc.?
**Respondent:** WhatsApp.

**Interviewer:** On WhatsApp, do people prefer written messages or voice notes?
**Respondent:** Mostly voice notes and videos.

**Interviewer:** What language do they use?
**Respondent:** Predominantly Sindhi, as this is mostly a Sindhi-speaking community.

**Interviewer:** Do they understand Urdu?
**Respondent:** Yes, 90-95% understand Urdu. While Sindhi is more common, there are other mixed communities here too, but the majority speak Sindhi or Urdu.

**Interviewer:** How common are TVs in households here?
**Respondent:** TVs are quite rare because mobiles have become more prevalent.

**Interviewer:** So, if we were to consider broadcasting an ad on TV, do you think it would be effective?
**Respondent:** Not as effective as using platforms like YouTube or WhatsApp. Social media on mobile phones would be much more beneficial.

**Interviewer:** Are there any specific channels or platforms used here to disseminate health messages?
**Respondent:** I’m not aware of any specific channels. But, as I mentioned, door-to-door visits can create some awareness. Messages can be communicated effectively this way, but there are no dedicated channels for this purpose.

**Interviewer:** Do people listen to the radio here?
**Respondent:** Perhaps 10% do.

**Interviewer:** How active is your community in participating in health-related programs and activities?
**Respondent:** Around 50-70% of the community participates—not everyone, but a significant portion does.

**Interviewer:** Are there any cultural or societal norms in your community that influence the understanding or dissemination of health messages? For instance, like mobile ownership or the authority of men in households?
**Respondent:** Yes, men’s opinions hold more weight. For example, when we go for vaccinations, women often say, *“Ask my husband first, then I’ll proceed.”*

**Interviewer:** Do all household members have their own mobile phones, or is it just one person?
**Respondent:** Usually, it’s just the men who have mobile phones.

**Interviewer:** Could this be a challenge? For instance, if we send messages, they’ll reach the men more often than the women.
**Respondent:** Yes, it will be less effective for women. Only those women who have access to a phone will receive the messages.

**Interviewer:** In these camps and activities, do men participate more, or do women also attend?
**Respondent:** Both men and women participate. When we go door-to-door or set up camps, we facilitate both, and women do attend as well.

**Interviewer:** To reach women, would it be necessary to involve men as intermediaries?
**Respondent:** Exactly. Men can then convince and bring their wives.

**Interviewer:** In your opinion, what additional resources or methods would be helpful in spreading health messages?
**Respondent:** Setting up camps and making door-to-door announcements would be the best approach. Also, involving local influencers from the area could be very effective, especially if it’s related to health. It would yield a good response.

**Interviewer:** Are there separate influencers for different communities here?
**Respondent:** No, mostly the influencers here are tied to political parties or are religious figures, like maulanas. People follow them, so their involvement could attract people to participate in such initiatives.

**Interviewer:** Are you referring to political party influencers?
**Respondent:** Yes, both political and religious influencers. If they are involved, it could improve participation in camps and make it easier to mobilize the community.

**Interviewer:** What about using posters, like putting them outside houses?
**Respondent:** That’s also a good idea, but only educated people will read and respond to them.

**Interviewer:** Do you want to share any additional thoughts about spreading health information?
**Respondent:** …

**Interviewer:** Or do you think there’s a pressing need for something in this area?
**Respondent:** There isn’t a lack of anything specific.

**Interviewer:** For example, can you talk about cleanliness?
**Respondent:** Cleanliness is an issue here, and there are many challenges. Aside from that, going door-to-door and creating awareness about health can be impactful. Facilitating the community by providing medicines or similar support would be very effective.

**Interviewer:** You mean offering incentives?
**Respondent:** Yes, exactly. People here want facilities. For instance, they face financial challenges and often request medicines or medical support for their children. For those in remote areas, traveling to nearby healthcare facilities is a significant issue. They would prefer door-to-door services or local facilities. Like how Aga Khan Hospital is already doing some outreach, they want more of these services, especially for pregnant women, as that’s a common concern. Their main priority is getting medical facilities, medicines, or doctors who can attend to them directly.

**Interviewer:** According to you, how many people prioritize cleanliness?
**Respondent:** I’d estimate that 30-40% of people here care about cleanliness and maintain it. However, 60% are indifferent, focusing on their work and personal matters instead.

**Interviewer:** Would you like to share anything else?
**Respondent:** That’s all.

**Interviewer:** Thank you.

**KII 4**

**Interviewer:** What is your age?
**Respondent:** 23.

**Interviewer:** What is the highest level of education you have completed?
**Respondent:** Graduation, BA.

**Interviewer:** What is your current employment position?
**Respondent:** Social coordinator.

**Interviewer:** Which organization?
**Respondent:** It’s an NGO, “Pakistan Fisher Folk Forum.”

**Interviewer:** What is your role in the community?
**Respondent:** I belong to the local community of Ibrahim Hyderi (IH). Specifically, around 80–85% of the community here is involved in fishing. My ancestors were also associated with fishing, and I am part of this community as well.

**Interviewer:** What exactly is your role as a social coordinator?
**Respondent:** As a social coordinator, I’d like to highlight that with the support of Pakistan Fisher Folk Forum, we’ve set up skill development centers for the community, particularly in IH. These centers provide opportunities to learn various skills like beautician training, stitching, and computer courses. Initially, we mobilized students, targeting them to join these centers after school hours to learn skills. Later, we also mobilized uneducated individuals to participate and acquire skills to earn a livelihood. We have many success stories of people who completed courses here, such as someone who got a job at a salon, another who opened a home-based parlor, or someone who set up their stitching business.

**Interviewer:** How long have you been working as a social coordinator?
**Respondent:** I have been working in this field for around 5 years.

**Interviewer:** In your opinion, what is the community’s perspective on health?
**Respondent:** When it comes to health facilities, government hospitals here are not in good condition. Either the doctors are unavailable, or medicines are missing. There are also many doctors who have private clinics and tend to prioritize those. I would request Aga Khan to establish a clinic in this area. They have zakat funds and could set up a system where the community pays a minimal amount to access their quality services. Alongside, they should also provide laboratory services, as there is no lab facility in any nearby area.

**Interviewer:** What do you think about the health awareness and practices of people in this community?
**Respondent:** People do consider health important since everyone knows that without good health, they would fall sick. Some improvements have been observed. However, challenges persist—for example, stagnant water after rainfall breeds mosquitoes, causing problems. While sanitation has significantly improved—new roads have been constructed, and garbage collection vehicles are now operational—the issue of rainwater drainage still remains unresolved.

**Interviewer:** How much do the residents prioritize their health?
**Respondent:** It varies. Some people, despite being financially stable, neglect their health, while others with limited financial means take better care of themselves. Overall, it’s a mix. For instance, filtered water is rarely used here. People mostly rely on tap water. Additionally, food sold on pushcarts and roadside stalls is commonly consumed, and only a few people buy items from proper markets.

**Interviewer**: What role do you think community leaders play in delivering health messages in informal settlements?
**Respondent**: In what way? What exactly do you want us to communicate to them?

**Interviewer**: As a social worker, or other similar workers, what role do community leaders have? Do people listen to them?
**Respondent**: For this, outreach sessions should be organized within the community. In these sessions, we should demonstrate everything practically and use documentaries to teach them lessons. For instance, in this area, both government and private schools exist, but hygiene is not emphasized in any of them. If you visit the government schools in this area, you'll see children with long nails and no knowledge of how to brush their teeth or wash their hands. Classrooms are often dirty. In government schools, we selected five students from each class and formed a group of 20 children as a "child club." We conducted sessions with them, showed them documentaries, and trained them. Now, they act as class leaders, responsible for monitoring hygiene, such as checking nails and maintaining classroom cleanliness. This approach can also be implemented with adults.

**Interviewer**: What about doing the same with adults?
**Respondent**: Yes, we can do similar sessions with adults. Just as we take steps with children, we can arrange sessions for adults as well.

**Interviewer**: What challenges do you face in delivering health messages to your community?
**Respondent**: Firstly, this community is wary of new people. When they see unfamiliar faces, they become suspicious. It's crucial to have someone from the local community to establish trust. Once they feel confident that the efforts are genuinely for their benefit, they start to cooperate. Secondly, they often ask questions stemming from fear, like during the coronavirus outbreak or regarding polio vaccines. It’s important to have a local person to help convince them.

**Interviewer**: Anything else? Are there rules in place? Do men’s opinions hold more weight?
**Respondent**: As you mentioned, women often follow what the men say. In many cases, it’s necessary to first gain the consent of the head of the household or the community leader. They are the ones who make decisions and influence others.

**Interviewer**: In your estimation, how many people in this community have mobile phones?
**Respondent**: In my opinion, almost all of the new generation—around 90 to 95%—own mobile phones. The older generation, those from before 2010, may not use them as much. They might have mobile phones, but most use keypad phones. The newer generation mainly uses smartphones.

**Interviewer**: What is the best way to deliver messages to this community?
**Respondent**: If you want to deliver messages to this community, especially to adults, outreach sessions should be organized in each area.

**Interviewer**: Some people may attend, and some may not?
**Respondent**: That could happen, but these areas are divided into neighborhoods, which we call mohallas. For example, IH has 52 mohallas and RG has around 17 to 18. Each mohalla has a leader or person in charge. These leaders hold influence over the community. It’s essential to convince them first. Once they are convinced, they bring the community members to attend the outreach sessions.

**Interviewer**: What are the most effective ways to deliver health messages in informal settlements? You mentioned outreach sessions earlier. What else?
**Respondent**: Yes, we can also use documentaries to convey messages. However, similar to outreach sessions, we would need to show these documentaries door-to-door in each neighborhood. Alternatively, we could convince social media influencers or social workers to share our documentaries on their pages. However, in older communities, while children are using mobile phones, the adults mostly don’t. Their focus is on fishing as their main occupation, and many are not literate. So, documentaries may not reach them effectively through social media. For them, outreach sessions would work better, while documentaries would suit the younger generation.

**Interviewer**: Who are these social media influencers?
**Respondent**: For instance, in the NGO sector, there are people on Facebook who work for the community. Examples include Waqas Karani from IH (Ibrahim Hyderi) and other individuals from IH.

**Interviewer**: Do they create vlogs or videos?
**Respondent**: Yes, they manage informational pages, like IH’s pages “Hyderi ki Behtari” and “Ibrahim Hyderi,” which the community follows. When there’s any activity at IH, they inform the community through these pages. Similarly, we have our page, “Pakistan Fisherfolk Forum.” Whenever we organize an activity, we post it there, and the community members watch it.

**Interviewer**: How many people watch these? Do younger children watch them more?
**Respondent**: Yes, younger generations watch them. Specifically, people born after 2001 or even 1995 would likely watch them. However, older individuals, such as elders, might not.

**Interviewer**: What percentage of women have phones?
**Respondent**: Common women generally don’t have phones, but young girls might. Overall, about 20-30% of women might have phones.

**Interviewer**: Facebook is used more frequently. What other platforms are used?
**Respondent**: Yes, YouTube is also used, but not as much. YouTube provides access to global content, so not all relevant videos reach the community. Facebook, on the other hand, allows for location-specific posts.

**Interviewer**: Is TikTok used as well?
**Respondent**: Yes, TikTok is used, primarily for entertainment purposes.

**Interviewer**: Are there any specific TV or radio channels that your community watches?
**Respondent**: I don’t think radio is popular anymore. As for TV channels, I personally watch ARY, but I’m not sure what others prefer.

**Interviewer**: Do people watch TV in your area?
**Respondent**: Yes, they do. However, most households have satellite dishes, and they watch Indian channels. They are not much interested in Pakistani channels.

**Interviewer**: What percentage of households have TVs?
**Respondent**: Around 70-80% of households have TVs. However, most of the local community involved in fishing spends their time at JT (possibly "jetty"). If you organize a session there, you’d find many people, but mostly men.

**Interviewer**: How actively does your community participate in health-related programs and activities?
**Respondent**: They aren’t very aware. Most are not literate enough to understand Urdu or English pamphlets or wall writings. If someone explains it to them, they might say, “Let us do our work,” without taking offense. However, if we make them understand in their own way and show them the benefits, 50-60% might participate.

**Interviewer**: Does your community follow any specific cultural or social norms, such as male dominance?
**Respondent**: Yes, women require permission from their parents or husbands to leave the house.

**Interviewer**: So, they’ll only participate if the men approve?
**Respondent**: Yes. That’s why we often arrange separate sessions for men and women. This approach is more acceptable.

**Interviewer**: Do households have touchscreen mobile phones?
**Respondent**: Yes, almost every household has them.

**Interviewer**: Are there usually more than one in a household?
**Respondent**: Yes, 50-60% of households have more than one phone. Even if parents don’t use them, their children definitely do.

**Interviewer:** What additional resources do you think are needed to deliver health messages effectively?

**Respondent:** In our community, if someone gets better after receiving treatment or medication from a certain place, they’ll keep going back there for life; they won’t consider going to another hospital. Here, Aga Khan has been doing a lot of work, particularly for children, but their focus is limited to children. They should extend their work to other age groups as well. There’s no 24-hour service available here. The clinics we have lack qualified doctors—most are compounders who set up their own clinics. The condition of government hospitals is not good. If Aga Khan opens clinics or laboratories here, it will build their reputation and trust within the community.

**Interviewer:** Anything else? What do you think about Lady Health Workers (LHWs) who go door-to-door?

**Respondent:** Regarding this, I believe they are doing good work going door-to-door. I remember a case in our neighborhood—a poor family had a girl with a hole in her heart. They didn’t have the financial means to get her treated at a good hospital. Aga Khan had a project running at that time, under which her operation and treatment were done at Aga Khan. That family is very grateful to Aga Khan. People here trust Aga Khan a lot. I think if you organize future sessions in these communities, people won’t refuse to attend. The trust has already been built.

**Interviewer:** Is there anything else you’d like to share?

**Respondent:** Regarding health, I’d like to say that during these sessions, you should also inform people about how viruses spread, how diseases are transmitted, and how they can protect themselves. They should also be educated about what kinds of food to consume, what to avoid from outside, and the type of water they should drink.

**Interviewer:** Thank you.

**KII 5**

**Interviewer**: How old are you?
**Respondent**: I am 42.

**Interviewer**: What is the highest level of education you have completed?
**Respondent**: I have completed matriculation in science.

**Interviewer**: What is your current employment status?
**Respondent**: I initially started working with Pakistan Fisher Forum as a social mobilizer. After that, I worked on a project (name unclear) and also with Indus Hospital under IRD in Ibrahim Hyderi, Rehri Goth, and Ali Akbar Shah as a field worker. Later, I worked on a UNICEF project under "IH" (name unclear), which involved house-to-house teaching for three years. Currently, I am unemployed.

**Interviewer**: How long have you been working in this field?
**Respondent**: I started this work around 2008.

**Interviewer**: What was your primary role in these projects related to the community?
**Respondent**: I worked as a social mobilizer. We formed NGOs in neighborhoods, both male and female groups, gathered them, and provided them with training. This training involved addressing community issues, learning how to resolve them, identifying where to seek help, and selecting capable individuals—mainly women—who could engage, speak up, or dedicate time. We organized these individuals into neighborhood-based groups.

**Interviewer**: How did you organize these groups? Did you go door-to-door?
**Respondent**: Yes, initially, we visited each neighborhood. First, we collaborated with resource persons in the area who identified individuals capable of contributing. Based on their suggestions, we gathered these individuals at a central location and conducted training sessions. We explained the concept of NGOs, how they function, and what role they could play.

**Interviewer**: What do people in your community think about health?
**Respondent**: When it comes to health, people lack knowledge and awareness. There isn’t enough education, and people are often hesitant or fearful. For instance, as a health worker, I faced significant challenges during the polio campaign. Many families refused to let their children get vaccinated, fearing unknown consequences. We had to work tirelessly to address their concerns.

Even in my family, my mother once pleaded with me to leave the job after a crowd protested and accused me of harming their children. It required constant efforts, including engaging resource persons such as religious leaders, particularly mosque imams, as well as schoolteachers, to help disseminate the message. Teachers, for example, could instill small lessons in children, who would then convey these messages to their mothers.

Gradually, we managed to cover a lot of ground, including routine immunizations and polio vaccinations. Today, thankfully, there are fewer challenges. However, recently, some workers distributing deworming tablets for children approached me. After COVID-19, people have become more fearful, suspecting that vaccines or medicines might introduce harmful viruses into their children’s bodies.

This fear has made it difficult for health workers to distribute even simple medications like deworming tablets. Some workers are afraid and even leave their jobs because of the resistance and criticism they face. When I worked, I would sit with people and reassure them by showing that I had given the same medicine to my own children. Unfortunately, there is still a lack of knowledge in the community, which makes such work challenging.

**Interviewer:** How did you interact directly with the women?
**Respondent:** In our areas, like Ibrahim Hyderi and Rehri Goth, women are involved in specific activities. For instance, they set up small stalls or prepare things at home to sell, like potatoes or chips, in the evenings. Many women from the neighborhood gather at these spots, discussing various topics. If you share a message there, it will spread among all the women in that community. They talk about what happens in their homes, so news travels quickly, whether it's true or not.

**Interviewer:** How many women are involved in such work?
**Respondent:** A lot. They go out in the morning and evening. After finishing their work, they gather at these spots for discussions. These gatherings are usually in the alleyways, and men don’t typically go there. It’s mostly women. The gatherings usually occur from 4 PM to 9 PM in the evening and from 8 AM to 10 AM in the morning.

**Interviewer:** So your workers approach them during these gatherings? Did it help?
**Respondent:** Yes.

**Interviewer:** What other challenges do you face in reaching the community, besides a lack of awareness?
**Respondent:** One major issue is the rising cost of living. Many women have to work, leaving their children with their mothers-in-law or mothers, who don’t always understand our messages. We need to find someone in the household, male or female, who is sensible and can grasp what we are saying. We convince them first, and through them, we reach the rest of the family. It’s particularly challenging with educated young women who work in factories or offices. They leave their children with elders who don’t understand much, making it difficult to get our message across to them.

**Interviewer:** How do you resolve this issue?
**Respondent:** We have to figure out their availability. We identify when they usually come home and directly talk to them, making sure the message sinks in so they can share it with their families.

**Interviewer:** What time do they usually return home?
**Respondent:** It depends on their jobs. For example, people working with Aga Khan Hospital come back around 3 PM, others return from companies at 5 PM or 6 PM. Sundays are a day off for most, so we take that opportunity as well.

**Interviewer:** Do these women successfully convince others?
**Respondent:** Yes, they manage to convince others, and it has benefited us.

**Interviewer:** Which languages are spoken in this community?
**Respondent:** Sindhi, Urdu, and Balochi are the main languages. Everyone generally understands them.

**Interviewer:** So you have to communicate in all these languages?
**Respondent:** Mostly Urdu and Sindhi. The Sindhi community is predominant, but the Baloch understand Urdu.

**Interviewer:** What other major issues do you face?
**Respondent:** Another issue is when we go, some women are cooking, or some are washing clothes. I’ve seen this a lot, and I’ve also done it myself when I was working in the IRD. I was assigned to the water pump area. There, many girls would refuse, they would just refuse outright. My supervisor then asked me to find out the reason for the refusals. One woman said she was washing clothes and didn’t have time. She left. I told her, "You’re washing clothes, just do it where you are standing, I’ll do my work here." So we have to sit with them, even if they are busy. Because it's our responsibility to convince them. Whenever they give us time, we have to sit with them, no matter what condition they are in. So the main reason for refusals here is usually that they’re busy with housework.

**Interviewer:** Besides door-to-door visits, have you tried other methods?
**Respondent:** Yes, we would look for a bigger gathering, like in the IH area, we would gather everyone. We’d go to one house, or meet in the yard, send vehicles, go door-to-door, and tell everyone to come as this house has a sensible woman or man.We’d formally invite them and then gather everyone at one place—whether it was my house, my friend’s house, or someone else’s house. We’d gather them and then deliver the message to the whole community. In this way, even if one person learned something from there, they would spread it to others, making it easier for us.

**Interviewer:** Have you ever used a smartphone?
**Respondent:** We have phones, but it’s not possible. You can’t communicate effectively over the phone, and it’s difficult to make them understand. Also, everyone is busy with their work, so we haven’t used phones for this purpose.

**Interviewer:** Do you think messages can be spread through phones, like via Facebook, WhatsApp, or Instagram?
**Respondent:** Most of the educated youth keep it to themselves. It doesn’t reach the mothers or older people in the family. They’ve read it, seen it, and then discarded it. So, if it reaches an educated person, they can do something with it, but nowadays, the younger generation mostly uses it, and even they don’t share important things at home.

**Interviewer:** How many people or households have smartphones here?
**Respondent:** Almost everyone has one, because even the kids play on their phones. Even the women working as maids in homes have phones.

**Interviewer:** So, do they also use Facebook, YouTube, etc.?
**Respondent:** Yes, but there’s also an issue with reading. If everyone isn't educated, then how will they read? That’s another problem.

**Interviewer:** According to you, how many people in this community are educated and using mobile phones or smartphones?
**Respondent:** Not many, but the uneducated ones are using voice messages for communication, and watching videos these days. This is mostly what’s happening now.

**Interviewer:** Do you think making a video or something like that would help?
**Respondent:** We did something similar with the Fisher Forum. For communities that didn’t understand anything, we did a theatre performance. We were given training on how to highlight the issue. Since theatre is also an entertainment medium, children, adults, and elderly people all enjoy watching it. Through this, using the resources we had, we tried to explain the issue. It was easier, and it’s a simple way to make people understand. They all watch it with interest, and they understand it as well.

**Interviewer:** So, did you do role plays in the theatre?
**Respondent:** Yes, we did everything ourselves.

**Interviewer:** And who was your target audience?
**Respondent:** We announced it, like in Rehri Goth. We said, "The Fisher Forum group is coming to present a theatre show." We didn’t specify exactly what the issue was, but everyone came for the entertainment, and then during the play, they understood. We even had to take some harsh feedback!

**Interviewer:** How did you convey the message that this was happening?
**Respondent:** We would go and formally invite people. Our resource person would tell them that the Fisher Forum is organizing a program, and they would then tell someone in the area, and that person would spread the word. The committees we set up also helped. There’s a secretary in each committee who is responsible for their neighborhood. They would make sure to let people know. This way, it didn’t become too much of a burden. The committees were divided by neighborhoods, so one person would cover one neighborhood, and another person would cover another. This way, it’s done easily.

**Interviewer:** How many neighborhoods are there in IH?
**Respondent:** I think there are over 52.

**Interviewer:** So, you don’t think it’s possible to communicate all of this through the phone?
**Respondent:** It’s not as effective through the phone. Everyone has a phone, but to make sure the message reaches the right person in the neighborhood, we need to identify who the right person is to spread the message. So, we have to do that too; it’s important to know who will ensure the message gets to everyone.

**Interviewer:** What other methods do you use?
**Respondent:** Through theatre, door-to-door visits, and when we go there, we have to convince people that doing these things will not harm the children but will benefit them. It’s very difficult to convince them, but it has to be done. Once the person doing the convincing agrees, others follow suit.

**Interviewer:** What about the health of mothers, besides children?
**Respondent:** The situation for mothers is similar. One major issue is that in IH, there is no specialized care. There is Aga Khan hospital and a Sindh government hospital. But when cases become complicated, they refer them to Jinnah or Civil Hospital. People avoid going due to fear of being turned away. Those who can afford it go to private clinics from the start, while the rest still rely on local midwives.

**Interviewer:** So, what problems are there at the Sindh government hospital?
**Respondent:** They don’t have enough resources. Things have improved somewhat, but if someone needs a C-section or has excessive bleeding, those cases are referred to Jinnah. Also, everyone knows they prescribe injections that are bought from outside, which many can’t afford, especially the daily wage workers in IH. It’s very difficult for them, both in terms of healthcare and education.

**Interviewer:** So, reaching these hospitals is also a problem, transportation-wise?
**Respondent:** Yes, absolutely. And there is no ambulance service at Sindh government hospitals. They have to hire a car at their own expense. No matter how much the fare is, they have to manage it on their own.

**Interviewer:** And how many people do you think go to Sindh government or Jinnah hospitals?
**Respondent:** Most people I know go with me, because going to Jinnah is not easy. People get pushed around, told to go here and there, and sometimes you need someone from the area who can speak up, someone who can argue if necessary. Sometimes, you even have to fight with the doctors to get things done. I go there with my whole family. No matter how sick someone is, they wait for me to go and take them. Sometimes, I even have to scold the doctors, but only then do they take action. Aga Khan’s center works during the day, but not at night. What if a pregnant woman faces a complication at night? Where would she go then?

**Interviewer:** Besides that, are there TVs in the houses?
**Respondent:** Yes, everyone has a TV. They all have a dish. The cable system has been shut down, but everyone has a dish now.

**Interviewer:** Which channels?
**Respondent:** Mostly Indian channels, there are no Pakistani channels. News channels are on too, and if an ad comes on…

**Interviewer:** Which news channels?
**Respondent:** Geo, ARY, Samaa. These are available in every house.

**Interviewer:** So, if an ad comes on, will they watch it?
**Respondent:** Yes, in this way.

**Interviewer:** What time do people watch TV the most?
**Respondent:** Whenever they’re free, it’s on all day. Many people watch it after work when they’re free.

**Interviewer:** Any specific time?
**Respondent:** In the evening. The working people come home at that time, and then they watch for an hour or two, and then they get tired and go to sleep.

**Interviewer:** What percentage of people attend theatre or similar activities?
**Respondent:** The entire community participates. Instead of just giving them written material, it’s better to read it out to them. Reading can bore people; not everyone understands every word. There might be terms that they won’t comprehend. So, this method is more effective. Posters and pamphlets are another option, but theatre has been particularly effective for us. It was much easier for people to understand.

**Interviewer:** People likely can’t read posters, right?
**Respondent:** Through pictures. You put up posters, and whoever sees them will know, for example, that this vaccine is good and not harmful. Gradually, they become convinced.

**Interviewer:** Are there any cultural or social norms in this community that affect the spread of health messages?
**Respondent:** Not many, really. If you see someone who seems knowledgeable or aware, we convince them, and they join in. Otherwise, resource persons help us a lot with this.

**Interviewer:** Is male dominance prevalent here?
**Respondent:** It’s everywhere, not just here.

**Interviewer:** So, you have to convince the men too?
**Respondent:** Yes, we have to. Some women even say, “Talk to our men.” So, we have to do that too.

**Interviewer:** What additional resources do you think are needed to spread health messages further in this community?
**Respondent:** Resources? There’s almost nothing. For example, we had a boat accident recently. Daily wagers rely on their work regardless of storms or government announcements. They need to work to feed their children. Because of this, they end up taking risks. Many people were injured in that accident—who was there to help them? We had to call news channels and ambulances from the city because there’s none in our area. Even then, because of traffic delays, those who could have survived also passed away. In the case of pregnancies or emergencies, people have to hire private transport, which not everyone can afford. Sindh Government has a 24-hour ambulance, but it often doesn’t have a driver available. The main issue here is the lack of 24/7 transport facilities, especially during emergencies.

**Interviewer:** You mentioned children use Facebook and YouTube. Do they spread the message further?
**Respondent:** They keep it to themselves.

**Interviewer:** What can be done to improve this?
**Respondent:** Since everyone watches TV at home, running ads repeatedly or providing information during program breaks can help. People will eventually see it, and it will start to make an impact.

**Interviewer:** How much time do you think this would take?
**Respondent:** It depends on people’s understanding and knowledge. Some people grasp things immediately, while others never do.

**Interviewer:** Is there anything else you’d like to share regarding health information?
**Respondent:** Health issues vary by region, but areas like IH and RG are highly underprivileged in today’s world. They lack the facilities that are commonly available elsewhere. For example, if a child gets injured at school, there isn’t even a first aid box on-site. No school, whether government or private, has a doctor or immediate medical support available to treat a child promptly. The same is true for those who go out to sea—there are no provisions for immediate medical care in case of an accident on the boat. People don’t know how to handle emergencies, nor do they have first aid kits.

**Interviewer:** So, we could spread messages about first aid. Are there any other topics?
**Respondent:** Regarding mothers and children, we have traditional birth attendants (dais) here, and the community places a lot of trust in them. It’s important to hold meetings with them, informing them that while they can continue their practices, they must also provide certain services or communicate essential information to the community. For example, alongside their blessings and care, they need to encourage mothers to use medical support like vaccinations during pregnancy. People listen to them in this regard.

**Interviewer:** Do you visit the homes of the dais?
**Respondent:** No, we conduct a proper survey to determine how many dais are in the area. A survey is essential. We then collect their contact information and organize meetings at a specific time and place, where we gather all of them and convince them. They, in turn, educate their communities.

**Interviewer:** Do you think visiting homes, conducting theatre performances, or other in-person methods are more effective than using phones?
**Respondent:** People avoid phones or anything involving reading or writing. If you distribute pamphlets to homes saying, “Please read this,” how many people will actually read them? Those who do will keep the information to themselves because no one has time. However, it’s easier to organize a specific time and place and spread the word that a program is happening at a particular location where everyone is welcome to attend.

**Interviewer:** Thank you.

**KII 6**

**Interviewer:** How old are you?
**Respondent:** 22.

**Interviewer:** How much education have you completed?
**Respondent:** I have completed Intermediate (12th grade).

**Interviewer:** What is your current job position?
**Respondent:** I am a supervisor for polio vaccination, and my salary is 30,000.

**Interviewer:** Which center do you work at, and what are your responsibilities?
**Respondent:** Our center is located at Sindh Government Hospital in Hyderi. We have a small setup there where all workers and supervisors gather, and the vaccines are made available. Our primary job is to distribute vaccines to the workers, who then take them into the community.

**Interviewer:** How long have you been doing this?
**Respondent:** It has been six years.

**Interviewer:** Do you interact directly with the community?
**Respondent:** Yes, absolutely. We have built a relationship with the community members. If I or one of my workers doesn’t visit, they ask about us. They know my name and the worker’s name and inquire why we didn’t come. It has become like a bond with them.

**Interviewer:** How often do you visit?
**Respondent:** We visit every one and a half months. Previously, our project required us to be in the field every month. Back then, I was in the field the entire month. Now, the project has changed. It used to be CBV, but now it’s SMT. So, we go every one and a half months, sometimes even after a month, and cover the entire community.

**Interviewer:** Do you administer vaccines other than polio?
**Respondent:** Yes, we also administer TCV vaccines and recently conducted a deworming campaign.

**Interviewer:** In your opinion, what is the community’s attitude towards health?
**Respondent:** Everyone has their own perspective—some are negative, and some are positive. There are people who are extremely negative and refuse to give polio drops. We approach such refusers directly, but we don’t immediately bring up the vaccine topic. We start with greetings and inquire about their well-being. Gradually, as their mood improves, we address the topic and ask why they didn’t give their child the drops. We ask them to share their reasons. If they share their concerns, we explain to them why their worries are unfounded. We work to dispel their doubts and misconceptions.

**Interviewer:** What’s the general attitude towards health in the community?
**Respondent:** Very little concern! They don’t take it seriously. For example, the Bengali community doesn’t take health seriously. They let their children play in unsanitary conditions without any concern, yet they have issues with giving polio drops. We explain to them that if they’re okay with their children playing in the dirt, won’t that make them sick? Why would the vaccine make them sick? If we intended to harm their children, we could add harmful substances to the medicines available in medical stores—there’s no need for workers to go door-to-door to administer vaccines. After such counseling, they usually agree.

**Interviewer:** In your opinion, how many children in the community have been vaccinated?
**Respondent:** This month, I had only one child left, and we covered them too. I had 1,450 children assigned, and all of them have been vaccinated.

**Interviewer:** In these informal settlements, if a health-related message needs to be conveyed, what role do community leaders like yourself play?
**Respondent:** You can involve influencers, counselors, and Lady Health Workers (LHWs) as well. Since they interact with the community daily, their words carry more weight. Someone who appears suddenly won’t be as effective, and people might not listen to them.

**Interviewer:** So, do you think they play a significant role in the community?
**Respondent:** Yes, absolutely. If even chronic refusers don’t agree, we take counselors or LHWs along. We introduce them to the refusers, and they counsel them about why vaccinating their children is important.

**Interviewer:** Who are these influencers?
**Respondent:** They are people like me, whom you’ve chosen for this role, and others who are influential in the area, such as community elders or respected individuals. For example, in fishing communities, people listen to their leaders.

**Interviewer:** While trying to convey health messages effectively, what challenges do you face in the community?
**Respondent:** The main issue is with the refusers—they tend to become hyper-aggressive. If they become excessively hostile, we sometimes have to involve the police for our safety. They say things like, “Why do you come to give these drops? We don’t want them. They are our children; we’ll decide for them. Who are you to tell us?” Pressuring them too much makes them even more aggressive. That’s why we try to talk to them calmly and avoid provoking them.

**Interviewer:** Are refusals more common among women or men?
**Respondent:** Women. Even if the men refuse, a woman can still ensure the child is vaccinated. She can say she didn’t vaccinate but still get the child vaccinated secretly. It’s not like husbands have time to come home from work and check if the polio worker came or not.

**Interviewer:** Why do women refuse? Are they influenced by household elders like in-laws?
**Respondent:** Yes, sometimes they say the husband has forbidden it, or the in-laws don’t allow it. Occasionally, they’ll say their brother-in-law is a religious scholar or Hafiz who has issued a fatwa against it. These are the kinds of challenges we face. But we directly address these refusers, collect their phone numbers, and call them to discuss the issue.

**Interviewer:** Have you asked them why they refuse?
**Respondent:** They claim it causes infertility or that it drives children away from religion. Sometimes they’re demanding and say things like, “If you’re giving these drops, provide us with rations too.” These demands come up. If their child is sick, we make medicines available to them as well.

**Interviewer:** Are there any other difficulties in reaching them?
**Respondent:** No, not really. As I said, our work requires us to reach them, and we always do.

**Interviewer:** You mentioned collecting phone numbers. Does it ever happen that women don’t answer the phone, or that they don’t have access to one?
**Respondent:** Yes, that happens. Sometimes they give their number but don’t answer the call. In those cases, we go directly to their homes. Or if they do answer, they might say, “My brother-in-law is home; you can come now.” Then we go with our teams.

**Interviewer:** What percentage of women answer their phones?
**Respondent:** Only about 10% answer. Most don’t.

**Interviewer:** Do people in this community have phones?
**Respondent:** Yes, they do, but they don’t share their numbers. These days, even children have mobile phones, and women also have them. They have smartphones, Android phones.

**Interviewer:** So, they don’t share their numbers?
**Respondent:** Yes, because they don’t trust everyone enough to give out their numbers.

**Interviewer:** How do you resolve this issue?
**Respondent:** We identify the whole area, marking which street has a child who is a refuser and noting landmarks where we can reach quickly. For example, if there’s a shop or a tuck shop nearby, we use those as references to reach the household quickly.

**Interviewer:** In these informal settlements, what do you think are the best ways to communicate health messages?
**Respondent:** We put up posters. We also appear on the news and share information in our groups. We have our own WhatsApp group, and we post statuses there. My friends are part of the group, and some of them are refusers. When they see these messages, they sometimes agree with us.

**Interviewer:** What kind of messages do you forward?
**Respondent:** We forward videos, like announcements that our campaign will run from certain dates. We also upload videos showing the harm done to children who didn’t receive vaccines.

**Interviewer:** Who speaks in these videos?
**Respondent:** These are videos from areas like Afghanistan where there are more refusers. We forward those videos, but we don’t create them ourselves.

**Interviewer:** Do you think these videos are effective?
**Respondent:** They are very effective. For example, one of my friends who was a refuser shared the video with her family, who were also refusers, and they started to agree.

**Interviewer:** Are these videos in Urdu?
**Respondent:** Yes, they are in Urdu.

**Interviewer:** In what language do you put up posters? Where do you place them?
**Respondent:** We use Urdu. We place posters at the corners of houses, and we also put up banners if we have them.

**Interviewer:** Are these helpful?
**Respondent:** Yes, they inform people that the vaccination team will be coming on certain dates. The posters also initiate discussions about the pros and cons of vaccinations. We start this process a week before the campaign begins.

**Interviewer:** Is the community educated?
**Respondent:** Most of them are educated. They can read Urdu.

**Interviewer:** You mentioned news on TV. Do you arrange for news segments on TV?
**Respondent:** No, the campaign owners arrange that. Media people like Hum TV and Samaa air the announcements. People who watch TV get the message. These days, though, most people watch on their mobile phones, which is why we also put up banners to reach those who don’t watch the news.

**Interviewer:** What time do they air the news?
**Respondent:** Usually two to three days before the campaign starts, in the evening. Community members often tell us that they saw it on the news.

**Interviewer:** Among all these methods, which do you think is the most effective?
**Respondent:** Door-to-door communication.

**Interviewer:** Apart from that?
**Respondent:** Other methods are less effective. If someone sees it on their mobile, they know about it. But for those who don’t, we have to go door-to-door to explain.

**Interviewer:** When you go door-to-door, do you ask if someone has seen the message on their mobile and shared it with others?
**Respondent:** Yes, we do. If they don’t fully understand our explanation, we show them the video directly. This helps, and they often agree after watching it.

**Interviewer:** Apart from this, are there other health campaigns conducted here?
**Respondent:** No. We conduct activities like mother sessions at home, or corner sessions. Our community mobilizers sometimes gather two or three people in a hotel and start their counseling there. For mother sessions, we go to a house, invite a few women, arrange tea, snacks, or refreshments on our own, and then start the counseling. We take everyone’s opinions—why they should or shouldn’t vaccinate their children, things like that. Refusers also attend these sessions, and many of them agree afterward. We used to hold more mother sessions before, but now we do them less frequently.

**Interviewer:** Do you think holding fewer sessions has reduced mothers’ interest in these campaigns?
**Respondent:** Yes, it has reduced their interest. However, our social mobilizers still go door-to-door every month. They hold discussions and conduct mother sessions.

**Interviewer:** What other methods do you use to deliver health messages?
**Respondent:** These are the main methods. If you set up a camp, you can also make announcements. We make announcements in mosques, such as telling people where the vaccination camp is located so they can go there. This is effective because people come after hearing the announcements.

**Interviewer:** Do religious leaders support these efforts?
**Respondent:** Just like with the general population, some support us while others refuse. The same is true for religious leaders.

**Interviewer:** Are there any local TV channels in this community?
**Respondent:** No, there aren’t any.

**Interviewer:** Do people here listen to the radio?
**Respondent:** No.

**Interviewer:** Nowadays, platforms like TikTok and YouTube are popular. Do people in your community make videos on these platforms?
**Respondent:** We can upload on TikTok, but no one from our community makes such videos.

**Interviewer:** What do you think about using TikTok, YouTube, WhatsApp, or Facebook to deliver messages?
**Respondent:** It’s an excellent idea because most people use these platforms.

**Interviewer:** Should the messages be in video format or text?
**Respondent:** Video.

**Interviewer:** Are there any pages or groups on Facebook for this community?
**Respondent:** Not really, because this is a Bengali community. They do use Facebook but don’t create pages or groups.

**Interviewer:** Do women in the community also use these platforms?
**Respondent:** Yes, they do. In our community, even if people skip meals, they are on their phones. Women spend a lot of time on TikTok. They also have WiFi at home.

**Interviewer:** How active is the community in participating in health-related activities?
**Respondent:** They try to participate as much as possible because it benefits them. For example, if we offer lunch or refreshments, a large number of people show up.

**Interviewer:** What if lunch isn’t provided?
**Respondent:** Then fewer people come—about 50% or 40%.

**Interviewer:** Are there any cultural or social norms in your community that affect how health messages are delivered, such as male dominance?
**Respondent:** It exists but very minimally. It’s not a major issue.

**Interviewer:** What about issues with mobile phones? For example, if there’s only one phone in the house, and women say they can’t receive the messages?
**Respondent:** Such issues do happen. In those cases, we give them our own phones so they can watch the videos. If they don’t watch them at that moment, we tell them to watch later. However, the understanding we can achieve through face-to-face interactions can’t be replicated through mobile phones. Face-to-face, we can understand their mindset, and they can understand ours.

**Interviewer:** What resources are needed to improve health awareness in your community?
**Respondent:** Like I mentioned, mother sessions are very effective. If we conduct them back-to-back, people start understanding. But if we create long gaps between sessions, they don’t understand at all. For instance, if we visit daily and engage in conversations, it works.

**Interviewer:** Are there any other resources needed?
**Respondent:** No.

**Interviewer:** Is there anything else you’d like to add? What are the best approaches?
**Respondent:** The best approach is to have good behavior, especially before going out into the field. When you meet people, don’t immediately start talking about the topic you came to discuss. First, ask them how they are doing, assess their mood and mindset, and ensure they are not upset. Then, talk about the purpose of your visit. If you immediately jump in and start accusing them, for example, “Why didn’t you vaccinate your child?” it won’t work. Also, send someone who speaks their language. Since most people in the community are Bengali, they will understand a Bengali speaker better.

**Interviewer:** What are the key topics that should be addressed?
**Respondent:** We should focus on cleanliness, inform them about common illnesses affecting children, and educate them on things that cause harm. For example, they should boil or filter water before drinking and not let children play in the dirt.

**Interviewer:** Thank you!

**KII 7**

**Interviewer:** Hello, Respondent. How old are you?
**Respondent:** I’m 27 years old.

**Interviewer:** What is your educational background?
**Respondent:** I have a Bachelor’s degree in Arts.

**Interviewer:** What is your role in the community?
**Respondent:** I am a teacher at an NGO school. I’ve worked with IRC on a project called Roshan Rastay. It focuses on “Right to Play,” where we engage children in activities. Our main project is Roshan Rastay.

**Interviewer:** Can you explain a bit about who you teach and what you do?
**Respondent:** Officers have set up a campus in my home, just like an organization working for welfare and development. For example, in government schools, there aren’t enough resources like notebooks, books, pencils, and other things children need. We provide those, along with school bags and toys.

**Interviewer:** So, you mainly teach children? Of what age group?
**Respondent:** Yes, I teach children aged 9 to 16 years.

**Interviewer:** How long have you been doing this work?
**Respondent:** I’ve been doing this since 2013.

**Interviewer:** Do you teach both boys and girls?
**Respondent:** Yes, I teach both boys and girls.

**Interviewer:** Do you teach at home, or is there a separate center?
**Respondent:** It’s my home, but they’ve turned it into a center. I have two rooms—one is used as a school, and the other is my living space.

**Interviewer:** How many children do you teach?
**Respondent:** I teach 31 children. They are divided into packages: Package A is for kindergarten and Grade 1; Package B is for Grades 2 and 3; Package C is for Grades 4 and 5. The project is completed in two years, and the children receive results. After that, they can continue studying in Grade 6.

**Interviewer:** Do the students continue studying afterward?
**Respondent:** Yes. Previously, many girls wouldn’t study further, but now, thank God, they do. For example, after the IRC project was completed, 4–5 girls enrolled in secondary school.

**Interviewer:** What is your perspective on the community’s attitude towards health?
**Respondent:** Aga Khan does excellent work in health. Previously, families had many children, and mothers would become weak due to a lack of spacing between pregnancies. Now they are promoting spacing. They also teach about feeding undernourished children—some mothers don’t know how to breastfeed properly due to a lack of education. When there’s education, there’s awareness. They show videos, provide information about malnourished children, how to breastfeed, and what to feed the child.

**Interviewer:** In general, how does the community care about health? How important do they consider it?
**Respondent:** Earlier, they didn’t pay much attention. But now, since children are getting educated and organizations like Aga Khan and others are raising awareness, there’s been improvement. However, some still lack awareness and don’t understand how harmful it can be to neglect health. For example, some parents refuse to vaccinate their children, thinking it’s harmful, but they don’t realize vaccines protect against serious diseases like measles. If vaccinated, the disease won’t have a severe impact; if not, it could even take the child’s life.

**Interviewer:** In your opinion, what percentage of people still lack awareness?
**Respondent:** Thank God, almost everyone gets vaccinated now—this is 100%. However, around 5% of people still don’t value education. They think their daughters don’t need to study or leave the house because it could harm them.

**Interviewer:** What are the best methods to deliver health-related messages in informal settlements?
**Respondent:** For instance, arranging a meeting where people are invited and informed, or going door-to-door to explain.

**Interviewer:** How important is the role of community leaders in this? Do people listen to them?
**Respondent:** Yes, they listen. About 90% of the people do.

**Interviewer:** While trying to effectively convey health messages in your community, what challenges do you face or could face?
**Respondent:** For example, the way people interpret information differs. It can be difficult to make them understand. However, if explained properly and they comprehend, there won’t be any major issues.
Another issue is livelihood. People often don’t have the resources to provide the best food for their children. Fishermen or laborers, for instance, are limited to just basic sustenance and cannot afford quality nutrition that would benefit their children’s health.

**Interviewer:** Are there any other difficulties? Do you face challenges in reaching these people, or are there any social norms that pose barriers?
**Respondent:** No.

**Interviewer:** Could mobile phones be used to reach out to people?
**Respondent:** Yes, this is an issue. For example, women in households often don’t use mobile phones; men mostly do. Since I teach, I have my own phone and permission to use it. Similarly, there are others who have phones, especially those working jobs, and we can reach them through this. But changing their deep-rooted cultural norms and systems will take time. It cannot happen overnight.

**Interviewer:** If people are invited to a meeting or shown a video, how many do you think will attend?
**Respondent:** Since I work with NGOs and look after children, I take care of them either from my own funds or with donations. I don’t see any resistance.

**Interviewer:** If we ask the community to come and discuss health-related topics, what challenges might they face in attending?
**Respondent:** No challenges. They will come; it’s for their benefit.

**Interviewer:** Do you think they will attend?
**Respondent:** Yes, they will come!

**Interviewer:** What are the best methods to spread health-related messages in informal settlements?
**Respondent:** Holding a meeting. For example, gathering everyone in one place to discuss, share opinions, and agree on beneficial ideas.

**Interviewer:** Are there spaces available to hold such meetings?
**Respondent:** Yes, we have open fields.

**Interviewer:** Have you conducted such meetings?
**Respondent:** Yes, we once organized a session with my colleagues. It addressed issues like early marriages of young girls and domestic violence against women. We held a session for women to raise awareness and encourage action. It was conducted at the school I run.

**Interviewer:** How did you invite people?
**Respondent:** I personally went door-to-door to call them.

**Interviewer:** These days, with the prevalence of mobile phones, do you think they could be used for this purpose?
**Respondent:** For those who have mobile phones, contacting them through it would work, and they would come. But for those who don’t have mobile phones, the message would have to be delivered in person.

**Interviewer:** Have you ever tried sending messages through mobile phones? Do people answer the phone and come?
**Respondent:** Yes, they answer the phone and come. Approximately 75% of them come.

**Interviewer:** Do they have basic phones or smartphones?
**Respondent:** Those with some education or jobs often have smartphones. Most men usually have smartphones.

**Interviewer:** When you need to contact someone, what platform do you use?
**Respondent:** I use both WhatsApp and regular calls.

**Interviewer:** What do you think is the best platform to use for sending a message? For example, people create groups on WhatsApp—would such a platform work?
**Respondent:** Talking on a call is the better way to convey timings. WhatsApp is sometimes on and sometimes not. If there are two or three days for the task, WhatsApp is better. But if it’s for the same day, it’s best to talk over a call.

**Interviewer:** Are there any community platforms we could use, like radio or TV?
**Respondent:** No. Most people just watch movies on TV.

**Interviewer:** What we mean is, could we put an ad on TV channels?
**Respondent:** Yes, that would be an excellent idea. A lot of people watch TV, especially at night. During breaks, messages about women’s health and intervals can be shared.

**Interviewer:** Are there any local TV channels?
**Respondent:** No, it’s all the usual channels—Geo, Hum, etc., the ones everyone watches.

**Interviewer:** How involved are people in health-related activities in your community?
**Respondent:** 90% of people participate.

**Interviewer:** Do women also participate?
**Respondent:** Yes, women are more involved.

**Interviewer:** Are they aware that health is a significant aspect?
**Respondent:** Yes, especially in terms of children. How to take care of them, what to feed them, their sleep schedules—these are all things mothers know about. For example, when a child falls ill, sleep and eating schedules change, and the mother adjusts accordingly.

**Interviewer:** Are there any cultural or societal norms in your community that impact how health messages are delivered? For example, male dominance?
**Respondent:** It used to be that way, but not anymore. For example, regarding children’s vaccinations, it used to be an issue—family planning vaccines weren’t allowed. But now, people are more aware, and there’s less resistance.

**Interviewer:** How did people become aware?
**Respondent:** It’s because they travel outside, work in companies, and hear about others having two children. They listen to doctors’ interviews, where doctors might have only one or two kids. Now, with so much around—TV, mobiles, and men using smartphones for everything—they learn about what’s good for health. This has increased their knowledge, and now they don’t object as much. Education is also improving in our area. Earlier, education wasn’t a focus, and girls weren’t allowed to study. Only boys would go and study. Now, even girls are going to school.

**Interviewer:** What has contributed most to this change?
**Respondent:** Initially, when I started teaching children, they came enthusiastically for a month or two. Then they stopped coming because they felt studying was a burden—reading and writing. So we went door-to-door to bring the children back. When they took an interest and started learning, they began enjoying it themselves. Interest is key, no matter the task. If someone is interested, the work gets done easily. Without interest, the task doesn’t get completed. We also worked hard and showed interest in the work. We called the children, and if we had resigned, saying we wouldn’t teach anymore, it would have ended there. We also needed the job and were passionate about being teachers.

**Interviewer:** What resources does your community need to improve health awareness?
**Respondent:** There is definitely a need. For example, poor households have many needs. Their financial situations are not stable.

**Interviewer:** If you want to deliver a message, what resources would you require?
**Respondent:** You simply need to explain things to them. For instance, if it’s about health—whether it’s the child’s or the mother’s health—you need a way to convey the information effectively. You should explain the benefits, like how this action will have these advantages and these disadvantages. That would make the work more effective.

**Interviewer:** What specific things would you need? Like transport? Money?
**Respondent:** Those are universal necessities. For instance, people won’t come unless they have the means to travel—there should be money for fare, otherwise how will they come? You also need funds, a vehicle. Things like tablets or mobile phones are necessary to deliver messages quickly so people know where to go and at what time. Going to homes takes time, and it’s exhausting. With mobile phones, you don’t waste time. If you walk there, time is lost too.

**Interviewer:** Is there anything else you’d like to share?
**Respondent:** Yes, spacing between children is beneficial. Earlier, families had many children, which led to food shortages, and the children were weak. The mother also became weak from giving birth repeatedly. Her nutrition would be compromised, making her weaker.

**Interviewer:** What other messages can we deliver?
**Respondent:** Regarding spacing, when there are fewer children, like a gap of 3 or 4 years, the child’s and mother’s health improve. For example, the first child grows older, starts walking, and then the next child comes. Before, one child would still be in the mother’s arms while the next one was born. With spacing, a child grows up, becomes independent, and the next one arrives. This way, the child’s nutrition and care improve. Otherwise, health deteriorates, and illnesses increase. Both the mother’s and child’s health improve with proper spacing.

**Interviewer:** Thank you!
