# Supplementary material for: Bridging the health literacy gap: Women and community stakeholders’ perspectives on maternal and child health communication in peri-urban Karachi, Pakistan
Source: PLOS Digit Health. 2026 Aug 27;5(8):e0001632. doi: 10.1371/journal.pdig.0001632 (PMC13521370; doi:10.1371/journal.pdig.0001632)
Supplement: Supplementary file 5 [file pdig.0001632.s006.docx]

**Title of manuscript: Bridging the Health Literacy Gap: Women and Community Stakeholders’ Perspectives on Maternal and Child Health Communication in Peri-urban Karachi, Pakistan**

**Manuscript No:**

Dear Reviewers,

Thank you for your valuable comments on our manuscript. The comments have been addressed and highlighted in the revised manuscript and the responses have been included in the table below.

| **S. No** | **Journal Requirements** | **Authors Responses** |
| --- | --- | --- |
| 1. | In the online submission form, you indicated that “The datasets used during the current study are available from the corresponding author on reasonable request.”.  All PLOS journals now require all data underlying the findings described in their manuscript to be freely available to other researchers, either  1. In a public repository,  2. Within the manuscript itself, or  3. Uploaded as supplementary information. | All translated FGDs and IDIs have been added as a supplementary file. |
| 2. | Please ensure that your Ethics Statement is available in its entirety at the beginning of your Methods section, under a subheading 'Ethics Statement'. | Added at line 124-125 on page 6 of the manuscript under the methods section. |
| 3. | Please provide an Author Summary. This should appear in your manuscript between the Abstract (if applicable) and the Introduction, and should be 150–200 words long. The aim should be to make your findings accessible to a wide audience that includes both scientists and non-scientists | This has been added before introduction on Page 3. |
| **S. No** | **Comments from Reviewer 1** | **Authors Responses** |
| 1. | I had one comment: In the Abstract (Line 33) I think there is a misprint of duplicated (visits) word you need to delete it. | This has been corrected on line 33 in the abstract on Page 2. |
| **S. No** | **Comments from Reviewer 2** | **Authors Responses** |
| 2. | The introduction would benefit from a more focused problem statement. Currently, it moves broadly from health literacy to digital health without clearly articulating the specific gap this study addresses. | The 2^nd^ last paragraph of introduction has been rephrased to make it more problem focused (lines 103-116). The gap this study aims to address has also been articulated on lines 117 through 122on Page 3 and 4 of the manuscript. |
| 3. | The rationale for focusing on peri-urban Karachi should be sharpened. While contextual information is provided, it would help to explicitly state why peri-urban settings present distinct communication challenges compared to rural or urban areas | This has been addressed from lines 103 to 106 of 2^nd^ last paragraph of introduction on page 5. |
| 4. | The authors should more clearly articulate how this study contributes beyond confirming known barriers such as phone access, electricity, and gender norms. What conceptual, methodological, or practical gap does this work fill? | We have edited the introduction section from lines 103 to 122 on Page 3 and 4 to add relevant details to justify the need for this work. |
| 5. | The introduction could better engage with digital health and communication theory. Even a light framing (for example, around access, trust, or modality fit) would help anchor the study analytically. | We have added this information and referenced aspects of digital health in lines 75 to 77 on Page 4. |
| 6. | The authors should clarify how the four sites were selected and whether they differ in meaningful ways that might influence communication practices. | This has been clarified in the study design and setting subsection in material and methods from lines 134-138 on page 6-7. |
| 7. | More detail is needed on the rationale for the sample sizes beyond saturation. For example, how did the team assess saturation across both participant groups? | This has been addressed in the last paragraph of data collection subsection in materials and methods from 195-205 on page 9. |
| 8. | The process of approaching 220 women but enrolling only 55 requires more reflection. The authors should discuss potential selection bias and how non-participation may have shaped the findings. | We agree with this statement. This has been addressed in the last paragraph of discussion from 468-469 on page 21. |
| 9. | The stakeholder sample is small and heterogeneous. The authors should reflect on how this diversity affects interpretation and whether certain stakeholder voices dominate the findings. | The stakeholders were intentionally kept heterogoneous to account for diverse voices from these communities. This has been addressed in lines 154 to 158 on page 7 of the manuscript. |
| 10. | The interview guides are referenced but not summarized in the manuscript. Including a brief description of key domains would help readers assess alignment with study aims. | Thank you for the comment. This has been addressed in lines 162 to 173 of page 8 of the manuscript. |
| 11. | The role of the PHCs and CHWs in recruitment should be discussed more explicitly, including how their involvement may have influenced participants’ responses. | This has been addressed in lines 175 to 182 of data collection subsection in materials and methods on Page 8. It has also been added as a limitation in lines 480 to 484 on Page 22 of discussion. |
| 12. | The authors should clarify how inductive the analysis truly was, given that the final themes closely mirror the study objectives. | This has been addressed in the lines 214-218 of data analysis section on page 10. |
| 13. | More detail is needed on how disagreements in coding were resolved and how decisions were documented. | This has also been addressed in line 226-229 on page 10 of the data analysis section. |
| 14. | The transition from codes to themes could be better explained to enhance transparency. | This has been addressed in the lines 214-218 on page 10 of the manuscript under the section of data analysis. |
| 15. | The positionality discussion could be more explicitly linked to the analysis. For example, how did reflexivity shape interpretation or theme development? | This has been addressed in line 238 to 243 of the manuscript on page 11 of the manuscript. |
| 16. | Some findings are presented in a descriptive manner without sufficient analytical interpretation. The authors should more clearly explain why certain patterns matter. | We have added analytical depth to the entire results section from line 282 to 394 on page 14 to 18 of the manuscript. |
| 17. | The themes occasionally overlap, particularly between facilitators, barriers, and suggested solutions. Consider tightening thematic boundaries or explicitly acknowledging these intersections. | Has been addressed in line 278-281 on pages 13-14 of the manuscript. |
| 18. | Quotations are helpful but could be more strategically selected. In some sections, fewer but more analytically rich quotes would strengthen the narrative. | Thank you for this suggestion. The team feels that the most relevant quotes have been added. However, we added an additional quote in 354-355 of page 17 of the manuscript. |
| 19. | The authors may consider briefly comparing where women’s and stakeholders’ perspectives converge or diverge, rather than presenting them in parallel. | A paragraph has been at the end of results to address this from lines 389 to 394 on page 18. |
| 20. | The discussion largely reiterates results and prior studies. It would benefit from deeper synthesis and clearer articulation of contribution. | Thank you for pointing this out. We have tried to provide a deeper synthesis in the entire discussion section from lines 396 to 485 on pages 18 to 22 of the manuscript. |
| 21. | The authors should clarify how their findings extend or nuance existing knowledge, rather than simply aligning with it. | As mentioned above, we have tried to improve on key findings from the current work. This is reflected in the revised discussion section from lines 396 to 485 of page 18 to 22 of the manuscript. |
| 22. | Given the journal’s focus, the discussion should more explicitly address implications for digital health design, scalability, and equity. | Thank you for highlighting this. These are critical to scaling digital solutions, it was not the focus of the current work. However, we have implied this in the conclusion section of the manuscript. Please refer to page 22 of the manuscript. |
| 23. | The role of gender norms and household power dynamics could be more analytically developed, as these appear central to communication effectiveness. | We agree with the reviewer’s comment. However, this study did not assess gender and socio-cultural norms directly. Having said that, there are some nuances that reflect in the use of technology which have been discussed in the revised results and discussion section. We have also addressed the lack of influential individuals such as men in the limitation section of the discussion section from lines 474 to 478 on page 21 and 22. |
| 24. | Additional limitations should be considered, including potential social desirability bias, transferability, and the influence of institutional affiliation on participant responses. | Added under discussion from line 467 to 485 on page 21-22 of the manuscript. |
| 25. | The authors could also reflect on the limited inclusion of men, despite their acknowledged influence on health decision-making. | This has been added in lines 476 to 478 of last paragraph of discussion. |
| 26. | The conclusion could be strengthened by offering clearer next steps for research, policy, or intervention development. | We have revised the conclusion section to address this comment. Please review lines 487 to 498 on page 22 of the manuscript. |
| 27. | Consider explicitly stating what digital health practitioners or policymakers should take away from this study. | We have revised the conclusion section to address this comment. Please review lines 496 to 498 on page 22 of the manuscript. |
