# Supplementary material for: Bridging the health literacy gap: Women and community stakeholders’ perspectives on maternal and child health communication in peri-urban Karachi, Pakistan
Source: PLOS Digit Health. 2026 Aug 27;5(8):e0001632. doi: 10.1371/journal.pdig.0001632 (PMC13521370; doi:10.1371/journal.pdig.0001632)
Supplement: Peer review history

# Peer review: Decision Letter 0

**DOI:** 10.1371/journal.pdig.0001632.r001

**Authors:** Mengyu Wang

16 Apr 2026

PDIG-D-25-01023

Bridging the Health Literacy Gap: Women and Community Stakeholders’ Perspectives on Maternal and Child Health Communication in Peri-urban Karachi, Pakistan

PLOS Digital Health

Dear Dr. Hoodbhoy,

Thank you for submitting your manuscript to PLOS Digital Health. After careful consideration, we feel that it has merit but does not fully meet PLOS Digital Health's publication criteria as it currently stands. Therefore, we invite you to submit a revised version of the manuscript that addresses the points raised during the review process.

Please submit your revised manuscript by Jun 15 2026 11:59PM. If you will need more time than this to complete your revisions, please reply to this message or contact the journal office at digitalhealth@plos.org. When you're ready to submit your revision, log on to https://www.editorialmanager.com/pdig/ and select the 'Submissions Needing Revision' folder to locate your manuscript file.

Please include the following items when submitting your revised manuscript:

* A letter that responds to each point raised by the editor and reviewer(s). You should upload this letter as a separate file labeled 'Response to Reviewers'. This file does not need to include responses to any formatting updates and technical items listed in the 'Journal Requirements' section below.

* A marked-up copy of your manuscript that highlights changes made to the original version. You should upload this as a separate file labeled 'Revised Manuscript with Track Changes'.

* An unmarked version of your revised paper without tracked changes. You should upload this as a separate file labeled 'Manuscript'.

If you would like to make changes to your financial disclosure, competing interests statement, or data availability statement, please make these updates within the submission form at the time of resubmission. Guidelines for resubmitting your figure files are available below the reviewer comments at the end of this letter.

We look forward to receiving your revised manuscript.

Kind regards,

Mengyu Wang

Section Editor

PLOS Digital Health

Leo Anthony Celi

Editor-in-Chief

PLOS Digital Health

orcid.org/0000-0001-6712-6626

**Journal Requirements:**

1. In the online submission form, you indicated that “The datasets used during the current study are available from the corresponding author on reasonable request.”.

All PLOS journals now require all data underlying the findings described in their manuscript to be freely available to other researchers, either

1. In a public repository,

2. Within the manuscript itself, or

3. Uploaded as supplementary information.

This policy applies to all data except where public deposition would breach compliance with the protocol approved by your research ethics board. If your data cannot be made publicly available for ethical or legal reasons (e.g., public availability would compromise patient privacy), please explain your reasons by return email and your exemption request will be escalated to the editor for approval. Your exemption request will be handled independently and will not hold up the peer review process, but will need to be resolved should your manuscript be accepted for publication. One of the Editorial team will then be in touch if there are any issues.

2. Please ensure that your Ethics Statement is available in its entirety at the beginning of your Methods section, under a subheading 'Ethics Statement'. It must include:

i) The name(s) of the Institutional Review Board(s) or Ethics Committee(s)

ii) The approval number(s), or a statement that approval was granted by the named board(s)

iii) (for human participants or donors) - A statement that formal consent was obtained (must state whether verbal/written) OR the reason consent was not obtained (e.g., anonymity).

vi) (for child participants) - Please update your Ethics Statement with confirmation that written informed consent was obtained from the parent/guardian of each participant under 18 years of age.

3. Please provide an Author Summary. This should appear in your manuscript between the Abstract (if applicable) and the Introduction, and should be 150–200 words long. The aim should be to make your findings accessible to a wide audience that includes both scientists and non-scientists. Sample summaries can be found on our website under Submission Guidelines:

https://journals.plos.org/digitalhealth/s/submission-guidelines#loc-parts-of-a-submission

If the reviewer comments include a recommendation to cite specific previously published works, please review and evaluate these publications to determine whether they are relevant and should be cited. There is no requirement to cite these works unless the editor has indicated otherwise.

**Additional Editor Comments (if provided):**

[Note: HTML markup is below. Please do not edit.]

**Reviewers' Comments:**

Reviewer's Responses to Questions

**Comments to the Author**

1. Does this manuscript meet PLOS Digital Health’s publication criteria? Is the manuscript technically sound, and do the data support the conclusions? The manuscript must describe methodologically and ethically rigorous research with conclusions that are appropriately drawn based on the data presented.

Reviewer #1: Yes

Reviewer #2: Yes

**********

2. Has the statistical analysis been performed appropriately and rigorously?

Reviewer #1: N/A

Reviewer #2: N/A

**********

3. Have the authors made all data underlying the findings in their manuscript fully available (please refer to the Data Availability Statement at the start of the manuscript PDF file)?

The PLOS Data policy requires authors to make all data underlying the findings described in their manuscript fully available without restriction, with rare exception. The data should be provided as part of the manuscript or its supporting information, or deposited to a public repository. For example, in addition to summary statistics, the data points behind means, medians and variance measures should be available. If there are restrictions on publicly sharing data—e.g. participant privacy or use of data from a third party—those must be specified.

Reviewer #1: Yes

Reviewer #2: No

**********

4. Is the manuscript presented in an intelligible fashion and written in standard English?

PLOS Digital Health does not copyedit accepted manuscripts, so the language in submitted articles must be clear, correct, and unambiguous. Any typographical or grammatical errors should be corrected at revision, so please note any specific errors here.

Reviewer #1: Yes

Reviewer #2: Yes

**********

5. Review Comments to the Author

Please use the space provided to explain your answers to the questions above. You may also include additional comments for the author, including concerns about dual publication, research ethics, or publication ethics. (Please upload your review as an attachment if it exceeds 20,000 characters)

**Reviewer #1:**  Really it is an intersting work, and hope policymakers will benifit from it and increase the efforts to enhance the health literacy. So, that I suggest to add a clear call for policymakers and even tele-communication companies.

I had one comment: In the Abstract (Line 33) I think there is a misprint of duplicated (visits) word you need to delete it.

Best wishes

**Reviewer #2:**  Overview and General Comments

Thank you for the opportunity to review this manuscript examining women’s and community stakeholders’ perspectives on maternal and child health communication in peri-urban Karachi, Pakistan. The topic is timely and important, particularly given growing interest in digital health, persistent health literacy gaps in LMICs, and the need for context-specific communication strategies. The inclusion of both message recipients and disseminators is a notable strength, and the study draws on a well-established community surveillance platform, which enhances credibility.

That said, while the manuscript addresses an important problem and presents rich qualitative data, several areas require strengthening to improve clarity, methodological transparency, analytical depth, and contribution to the digital health and health communication literature. Many of the findings are intuitive and align with existing work, so the paper would benefit from clearer articulation of what is novel, how the analysis advances current understanding, and how the findings translate into actionable insights for digital health design and implementation.

Introduction

• The introduction provides a clear overview of health literacy, its relevance to maternal and child health, and the growing role of digital platforms.

• The authors appropriately situate the study within LMIC contexts and cite relevant global and regional literature.

• The introduction would benefit from a more focused problem statement. Currently, it moves broadly from health literacy to digital health without clearly articulating the specific gap this study addresses.

• The rationale for focusing on peri-urban Karachi should be sharpened. While contextual information is provided, it would help to explicitly state why peri-urban settings present distinct communication challenges compared to rural or urban areas.

• The authors should more clearly articulate how this study contributes beyond confirming known barriers such as phone access, electricity, and gender norms. What conceptual, methodological, or practical gap does this work fill?

• The introduction could better engage with digital health and communication theory. Even a light framing (for example, around access, trust, or modality fit) would help anchor the study analytically.

Methods

• The study setting is well described, and the integration with an existing surveillance system is a major strength.

• The use of FGDs and KIIs is appropriate for the research questions.

• The authors should clarify how the four sites were selected and whether they differ in meaningful ways that might influence communication practices.

• More detail is needed on the rationale for the sample sizes beyond saturation. For example, how did the team assess saturation across both participant groups?

• Eligibility criteria for women and stakeholders are clearly stated.

• The use of random selection for women from the surveillance database is a strength.

• The process of approaching 220 women but enrolling only 55 requires more reflection. The authors should discuss potential selection bias and how non-participation may have shaped the findings.

• The stakeholder sample is small and heterogeneous. The authors should reflect on how this diversity affects interpretation and whether certain stakeholder voices dominate the findings.

• Ethical procedures and consent processes are clearly described.

• The use of locally relevant languages and attention to translation rigor are commendable.

• The interview guides are referenced but not summarized in the manuscript. Including a brief description of key domains would help readers assess alignment with study aims.

• The role of the PHCs and CHWs in recruitment should be discussed more explicitly, including how their involvement may have influenced participants’ responses.

• The analytic approach is clearly named and appropriately applied.

• The use of multiple coders and consensus-building strengthens credibility.

• The authors should clarify how inductive the analysis truly was, given that the final themes closely mirror the study objectives.

• More detail is needed on how disagreements in coding were resolved and how decisions were documented.

• The transition from codes to themes could be better explained to enhance transparency.

• The inclusion of a positionality statement is a strong and appropriate choice.

• The authors thoughtfully acknowledge power dynamics and institutional affiliation.

• The positionality discussion could be more explicitly linked to the analysis. For example, how did reflexivity shape interpretation or theme development?

• The authors might consider briefly reflecting on how participants may have perceived the researchers and how this influenced data richness.

Results

• The themes are clearly organized and supported by participant quotations.

• The inclusion of both women’s and stakeholders’ perspectives adds depth.

• Some findings are presented in a descriptive manner without sufficient analytical interpretation. The authors should more clearly explain why certain patterns matter.

• The themes occasionally overlap, particularly between facilitators, barriers, and suggested solutions. Consider tightening thematic boundaries or explicitly acknowledging these intersections.

• Quotations are helpful but could be more strategically selected. In some sections, fewer but more analytically rich quotes would strengthen the narrative.

• The authors may consider briefly comparing where women’s and stakeholders’ perspectives converge or diverge, rather than presenting them in parallel.

Discussion

• The discussion effectively situates findings within existing LMIC and Pakistan-based literature.

• The argument for hybrid communication strategies is well supported.

• The discussion largely reiterates results and prior studies. It would benefit from deeper synthesis and clearer articulation of contribution.

• The authors should clarify how their findings extend or nuance existing knowledge, rather than simply aligning with it.

• Given the journal’s focus, the discussion should more explicitly address implications for digital health design, scalability, and equity.

• The role of gender norms and household power dynamics could be more analytically developed, as these appear central to communication effectiveness.

Limitations

• The authors appropriately acknowledge limitations related to sampling and generalizability.

• Additional limitations should be considered, including potential social desirability bias, transferability, and the influence of institutional affiliation on participant responses.

• The authors could also reflect on the limited inclusion of men, despite their acknowledged influence on health decision-making.

Conclusion

• The conclusion is concise and consistent with the findings.

• The emphasis on hybrid approaches is appropriate.

• The conclusion could be strengthened by offering clearer next steps for research, policy, or intervention development.

• Consider explicitly stating what digital health practitioners or policymakers should take away from this study.

**********

6. PLOS authors have the option to publish the peer review history of their article (what does this mean?). If published, this will include your full peer review and any attached files.

**Do you want your identity to be public for this peer review?**  If you choose “no”, your identity will remain anonymous but your review may still be made public.

For information about this choice, including consent withdrawal, please see our Privacy Policy.

Reviewer #1: No

Reviewer #2: No

**********

[NOTE: If reviewer comments were submitted as an attachment file, they will be attached to this email and accessible via the submission site. Please log into your account, locate the manuscript record, and check for the action link "View Attachments". If this link does not appear, there are no attachment files.]

**Figure resubmission:**

While revising your submission, we strongly recommend that you use PLOS’s NAAS tool (https://ngplosjournals.pagemajik.ai/artanalysis) to test your figure files. NAAS can convert your figure files to the TIFF file type and meet basic requirements (such as print size, resolution), or provide you with a report on issues that do not meet our requirements and that NAAS cannot fix.

After uploading your figures to PLOS’s NAAS tool - https://ngplosjournals.pagemajik.ai/artanalysis, NAAS will process the files provided and display the results in the "Uploaded Files" section of the page as the processing is complete. If the uploaded figures meet our requirements (or NAAS is able to fix the files to meet our requirements), the figure will be marked as "fixed" above. If NAAS is unable to fix the files, a red "failed" label will appear above. When NAAS has confirmed that the figure files meet our requirements, please download the file via the download option, and include these NAAS processed figure files when submitting your revised manuscript.

**Reproducibility:**

To enhance the reproducibility of your results, we recommend that authors of applicable studies deposit laboratory protocols in protocols.io, where a protocol can be assigned its own identifier (DOI) such that it can be cited independently in the future. Additionally, PLOS ONE offers an option to publish peer-reviewed clinical study protocols. Read more information on sharing protocols at https://plos.org/protocols?utm_medium=editorial-email&utm_source=authorletters&utm_campaign=protocols

# Author comment: Author response to Decision Letter 1

**DOI:** 10.1371/journal.pdig.0001632.r002

12 May 2026

Attachment Submitted filename: Response to Reviewers.docx

# Peer review: Decision Letter 1

**DOI:** 10.1371/journal.pdig.0001632.r003

**Authors:** Mengyu Wang

19 Jul 2026

Bridging the Health Literacy Gap: Women and Community Stakeholders’ Perspectives on Maternal and Child Health Communication in Peri-urban Karachi, Pakistan

PDIG-D-25-01023R1

Dear Dr. Hoodbhoy,

We are pleased to inform you that your manuscript 'Bridging the Health Literacy Gap: Women and Community Stakeholders’ Perspectives on Maternal and Child Health Communication in Peri-urban Karachi, Pakistan' has been provisionally accepted for publication in PLOS Digital Health.

Before your manuscript can be formally accepted you will need to complete some formatting changes, which you will receive in a follow-up email from a member of our team.

Please note that your manuscript will not be scheduled for publication until you have made the required changes, so a swift response is appreciated.

IMPORTANT: The editorial review process is now complete. PLOS will only permit corrections to spelling, formatting or significant scientific errors from this point onwards. Requests for major changes, or any which affect the scientific understanding of your work, will cause delays to the publication date of your manuscript.

If your institution or institutions have a press office, please notify them about your upcoming paper to help maximize its impact. If they'll be preparing press materials, please inform our press team as soon as possible -- no later than 48 hours after receiving the formal acceptance. Your manuscript will remain under strict press embargo until 2 pm Eastern Time on the date of publication. For more information, please contact digitalhealth@plos.org.

Thank you again for supporting Open Access publishing; we are looking forward to publishing your work in PLOS Digital Health.

Best regards,

Mengyu Wang, Ph.D.

Section Editor

PLOS Digital Health

***********************************************************

**Additional Editor Comments (if provided):**

**Reviewer Comments (if any, and for reference):**

Reviewer's Responses to Questions

**Comments to the Author**

1. If the authors have adequately addressed your comments raised in a previous round of review and you feel that this manuscript is now acceptable for publication, you may indicate that here to bypass the “Comments to the Author” section, enter your conflict of interest statement in the “Confidential to Editor” section, and submit your "Accept" recommendation.

Reviewer #2: All comments have been addressed

**********

2. Does this manuscript meet PLOS Digital Health’s publication criteria? Is the manuscript technically sound, and do the data support the conclusions? The manuscript must describe methodologically and ethically rigorous research with conclusions that are appropriately drawn based on the data presented.

Reviewer #2: Yes

**********

3. Has the statistical analysis been performed appropriately and rigorously?

Reviewer #2: N/A

**********

4. Have the authors made all data underlying the findings in their manuscript fully available (please refer to the Data Availability Statement at the start of the manuscript PDF file)?

The PLOS Data policy requires authors to make all data underlying the findings described in their manuscript fully available without restriction, with rare exception. The data should be provided as part of the manuscript or its supporting information, or deposited to a public repository. For example, in addition to summary statistics, the data points behind means, medians and variance measures should be available. If there are restrictions on publicly sharing data—e.g. participant privacy or use of data from a third party—those must be specified.

Reviewer #2: Yes

**********

5. Is the manuscript presented in an intelligible fashion and written in standard English?

PLOS Digital Health does not copyedit accepted manuscripts, so the language in submitted articles must be clear, correct, and unambiguous. Any typographical or grammatical errors should be corrected at revision, so please note any specific errors here.

Reviewer #2: Yes

**********

6. Review Comments to the Author

Please use the space provided to explain your answers to the questions above. You may also include additional comments for the author, including concerns about dual publication, research ethics, or publication ethics. (Please upload your review as an attachment if it exceeds 20,000 characters)

Reviewer #2: (No Response)

**********

7. PLOS authors have the option to publish the peer review history of their article (what does this mean?). If published, this will include your full peer review and any attached files.

**Do you want your identity to be public for this peer review?**  If you choose “no”, your identity will remain anonymous but your review may still be made public.

For information about this choice, including consent withdrawal, please see our Privacy Policy.

Reviewer #2: No

**********
